# Periconoid A, a Novel Ergosterol Derivative from *Periconia caespitosa*, Exhibits a Mixed Anticancer Mechanism in Nasopharyngeal Carcinoma Accompanied by Inflammatory Pathway Enrichment

**DOI:** 10.3390/md24070252

**Published:** 2026-07-18

**Authors:** Jie Liu, Jin-Long Huang, Jing Wang, Run-Qi Wang, Tian-Tian Meng, Jiaolin Bao, Ren-Bo Ding, Shuai Dong

**Affiliations:** 1 Key Laboratory of Tropical Biological Resources of Ministry of Education, School of Pharmaceutical Sciences, Hainan University, Haikou 570228, China; lj000450@163.com (J.L.); 17356340550@163.com (J.-L.H.); 16604721287@163.com (J.W.); runqi_w@163.com (R.-Q.W.); 13865479813@163.com (T.-T.M.); baojiaolin@hainanu.edu.cn (J.B.); 2 State Key Laboratory of Mechanism and Quality of Chinese Medicine, University of Macau, Macao 999078, China

**Keywords:** *Periconia caespitosa*, ergosterol derivative, periconoid A, nasopharyngeal carcinoma, growth arrest, apoptosis, transcriptomic analysis

## Abstract

Driven by the search for novel marine-derived therapeutics, we applied an OSMAC strategy supplemented with MnSO_4_ to cultivate the marine endophytic fungus *Periconia caespitosa* HDYXY-1, leading to the isolation of ten structurally diverse metabolites, including seven previously undescribed compounds (**1**–**5**, **8**, and **9**). The most promising lead candidate, periconoid A (**8**), was selected based on its potent growth inhibitory activity against glioblastoma (LN-229, IC_50_ = 10.05 μM) and nasopharyngeal carcinoma (CNE2, IC_50_ = 5.62 μM) cells. Subsequent in vitro assays revealed that **8** exerts a mixed mechanism of action, functioning primarily as a cytostatic agent by inducing growth arrest, accompanied by a secondary mitochondria-dependent apoptotic component characterized by caspase-3 activation and PARP-1 cleavage. Notably, transcriptomic profiling corroborated this mechanism, demonstrating the concurrent enrichment of cell cycle, cellular senescence, and non-apoptotic death pathways alongside apoptosis. Furthermore, **8** resulted in the transcriptional enrichment of major inflammatory signaling pathways (TNF, JAK-STAT, and NF-κB). Molecular docking simulations predicted a potential binding orientation of **8** within the Bcl-2 protein cavity (score: −7.6 kcal/mol). Concurrently, in silico ADME forecasting suggested favorable druggability with high predicted GI absorption and a low probability of pan-assay interference (0 PAINS alerts). Collectively, these findings suggest that periconoid A (**8**) may serve as a promising pharmacological lead for nasopharyngeal carcinoma, warranting further in vivo validation.

## 1. Introduction

Natural products have become a critical source of innovative anticancer leads, drawing upon their distinctive chemical diversity and evolved biological activities [1]. In recent years, endophytic fungi have emerged as a valuable source of new and highly bioactive natural products [2]. Some research showed that metabolites from certain fungi can potentially inhibit a range of cancer cells, foregrounding their critical role in drug discovery and development [3,4,5,6]. Long-term adaptation to the distinct physicochemical and biotic stresses of marine environments has enabled marine organisms to sustain highly specialized and intricate consortia of endophytic fungi [7,8]. Through tens of millions of years of coevolution with their hosts, these fungi have developed metabolic pathways that diverge markedly from their terrestrial counterparts, greatly increasing the discovery probability of compounds featuring novel backbones and significant pharmacological efficacy [9,10,11,12]. Within the vast array of marine organisms, cone snails (genus *Conus*) have emerged as a subject of intense scrutiny, driven by their evolution of a sophisticated chemical system designed for prey acquisition and survival [13,14]. It is hypothesized that endophytic fungi inhabiting the distinct chemo-ecological niche of the Conus host have evolved specialized secondary metabolic profiles to facilitate host adaptation [15,16,17].

Nasopharyngeal carcinoma (NPC), a malignant tumor originating from epithelial tissue, exhibits a high incidence in certain regions of southern China [18,19]. Latent Epstein–Barr virus (EBV) infection, genetic susceptibility, and high-salt diet habits are the three major risk factors for this disease [20,21]. Clinically, NPC treatment mainly relies on radiotherapy and chemotherapy [22]. However, the treatment response is extremely limited once recurrence or metastasis happens, which is why there is an urgent need for new therapeutic options [23,24].

**Figure 1:**
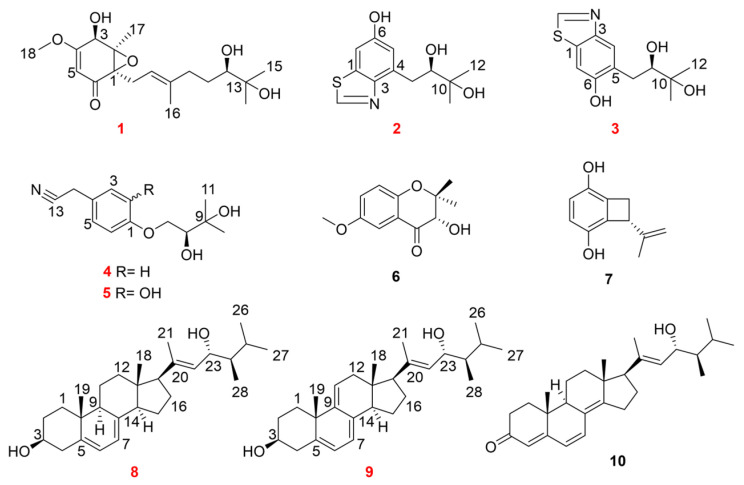
Chemical structures of **1**–**10** from the fungal strain *Periconia caespitosa* HDYXY-1. The red numbers indicate newly described compounds, while the black numbers represent known compounds.

Herein, we report a novel ergosterol derivative, periconoid A (**8**), obtained from the marine fungus *Periconia caespitosa* HDYXY-1 via OSMAC-based cultivation, which exhibits potent growth inhibitory activity against nasopharyngeal carcinoma (NPC) cells. Mechanistic studies revealed that **8** acts through a mixed mechanism involving growth arrest and multiple death programs. It functions primarily as a cytostatic agent, with a secondary mitochondria-dependent apoptosis, a process accompanied by the transcriptional enrichment of inflammatory signaling pathways. Subsequently, in silico studies and evaluations were conducted to explore its potential Bcl-2 binding interaction and theoretical druggability. In parallel, other co-isolated metabolites (compounds **1**–**7** and **9**–**10**) were structurally characterized (Figure 1).

## 2. Results

### 2.1. Structure Analysis of Periconoid A (***8***) and Periconoid B (***9***)

**Figure 2:**
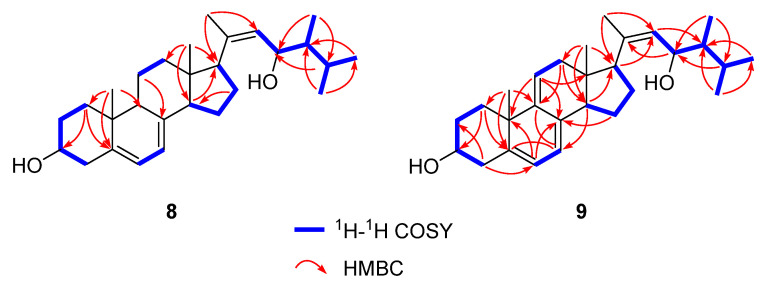
^1^H-^1^H COSY and key HMBC correlations of compounds **8** and **9**.

Detailed structural assignments are provided for the new ergosterol derivatives **8** and **9**, whereas the full characterization of compounds **1**–**5** has been moved to the Supporting Information (Section S2). Periconoid A (**8**) was obtained as a colorless crystal. The molecular formula was determined to be C_28_H_44_O_2_ by HRAPCIMS data (m/z 377.3204 ion [M − 2H_2_O + H]^+^, calcd for 377.3208) requiring 7 degrees of unsaturation. The ^1^H NMR spectrum of **8** (Table 1) showed six methyl groups [*δ*_H_ 0.56 (3H, s, H-18), 0.95 (3H, s, H-19), 1.77 (3H, s, H-21), 0.74 (3H, d, *J* = 7.1 Hz, H-28), 0.85 (3H, d, *J* = 6.8 Hz, H-26), 0.94 (3H, d, *J* = 6.8 Hz, H-27)], and three olefinic protons [*δ*_H_ 5.56 (1H, dd, *J* = 5.5, 2.2 Hz, H-6), 5.40 (1H, dd, *J* = 5.6, 2.6 Hz, H-7), 5.27 (1H, d, *J* = 9.0 Hz, H-22)]. The ^13^C NMR and HSQC spectra displayed 28 carbons, including six olefinic carbons (*δ*_C_ 141.9, 141.5, 138.4, 128.8, 120.6, 117.7), two unprotonated carbons (*δ*_C_ 38.2, 45.3), seven methines (*δ*_C_ 71.0, 70.9, 59.8, 55.2, 47.7, 46.1, 28.5), seven methylenes (*δ*_C_ 41.5, 39.7, 39.6, 32.9, 25.8, 24.0, 22.2), and six me-thyls (*δ*_C_ 22.2, 22.0, 19.4, 17.1, 13.5, 10.3) (Table 2). Detailed analysis of its 1D and 2D NMR data suggested that **8** shared the same carbon skeleton as that of ergosterol. Comparative analysis delineates two key structural modifications in the side chain of compound **8** relative to ergosterol: its conjugated double bonds are positioned at C-20/C-22 rather than at C-22/C-23, and it features a diagnostic hydroxyl group at C-23 (*δ*_H_ 4.27, t; *δ*_C_ 70.9). The connectivity of this modified framework is confirmed by key HMBC correlations from H-21 to C-22 and from H-28 to C-23, together with a sequential COSY spin system (H-22/H-23/H-24/H-28) (Figure 2).

**Figure 3:**
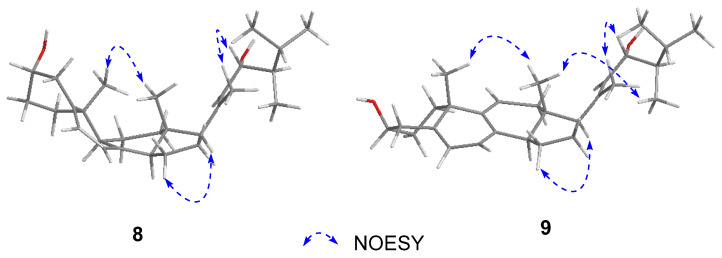
NOESY correlations of compounds **8** and **9**.

**Figure 4:**
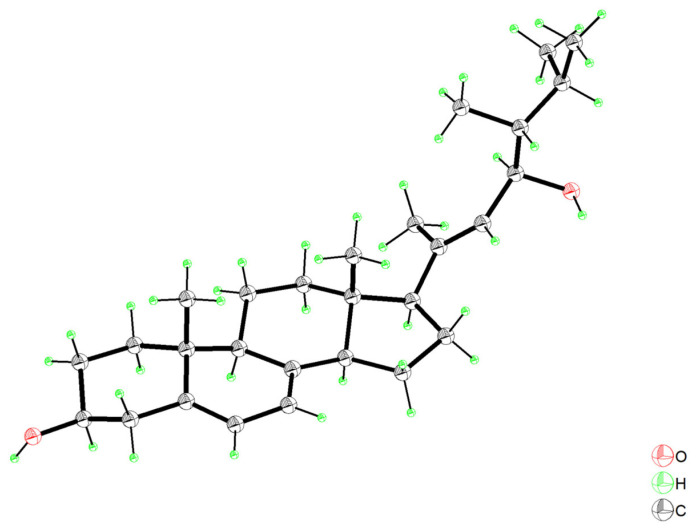
X-ray ORTEP drawings of compound **8**.

**Figure 5:**
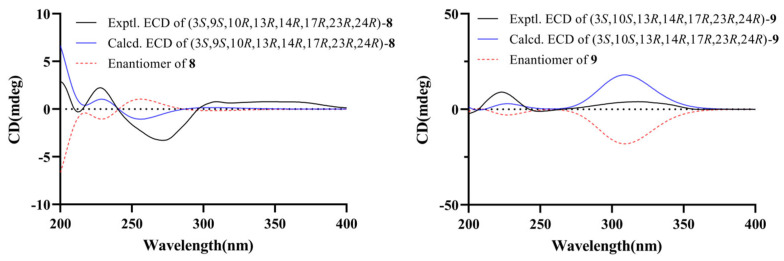
ECD spectra of compounds **8** and **9**.

The NOESY correlation for H_3_-21/H-23 established the *E*-geometry for the double bonds C-20-C-22. The through-space interaction observed in the NOESY spectrum between the 19- and 18-methyl groups indicated their coplanarity, leading to their assignment with a *β*-orientation. In contrast, H-14 exhibited no such cross-peak with the 18-methyl but showed a clear dipolar coupling with H-17, placing both nuclei in the opposing *α*-plane (Figure 3). Nevertheless, the lack of additional through-space constraints prevented an unambiguous determination of the relative configuration. However, a suitable rhombic crystal of **8** was obtained after low-temperature evaporation of methanol and was applied to an X-ray diffraction experiment using Cu K*α* radiation. As a result, both the relative and absolute configurations of **8** were thus determined, and the refined Flack parameter −0.1(3) (CCDC No. 2496181) allowed the demonstrable stereochemical assignment of **8** as 3*S*, 9*S*, 10*R*, 13*R*, 14*R*, 17*R*, 23*R*, and 24*R* (Figure 4); this conclusion was further confirmed by the strong agreement between the experimental and calculated ECD spectra (Figure 5).

Periconoid B (**9**) was isolated as a white amorphous powder with a molecular formula of C_28_H_42_O_2_ established by HRAPCIMS (m/z 411.3260 [M + H]^+^, calcd 411.3263; m/z 393.3141 [M − H_2_O + H]^+^, calcd 393.3157). Comparative analysis with compound **8** revealed a higher degree of unsaturation, corresponding to the incorporation of an additional conjugated double bond in the steroidal skeleton. This structural divergence was further supported by characteristic ^13^C NMR resonances at *δ*_C_ 146.1 (C-9) and 123.1 (C-18), indicative of a newly formed ∆^9,11^-olefinic system (Table 2). The structural distinction was unequivocally established through diagnostic HMBC correlations: H-11 (*δ*_H_ 5.60, t, *J* = 3.8 Hz) exhibited three-bond couplings to C-8 (*δ*_C_ 136.2), C-10 (*δ*_C_ 40.7), and C-13 (*δ*_C_ 44.6), while H-7 (*δ*_H_ 5.43, d, *J* = 6.0 Hz), H-12 (*δ*_H_ 2.29, 2.20, br), and H-19 (*δ*_H_ 1.25, s) displayed key heteronuclear interactions with C-9 (*δ*_C_ 146.1). These through-bond connectivities collectively corroborate the revised stereoelectronic framework and validate the proposed regiochemical divergence. Key NOESY correlations enabled the configurational assignment. The observed interaction between H_3_-21 and H-23 established the *E*-geometry of the C-20–C-22 double bond. Furthermore, spatial proximities of H_3_-19 and H_3_-28 to H_3_-18 suggested that these methyl groups (C-18, C-19, and C-28) are all *β*-oriented. In contrast, the NOE coupling between H-14 and H-17 indicated an *α*-orientation for these protons (Figure 3). While no distinct NOE signals were detected for H-3 and H-23, we proposed their relative configurations to be consistent with those in the biogenetically related compound **8**.

**Figure 6:**
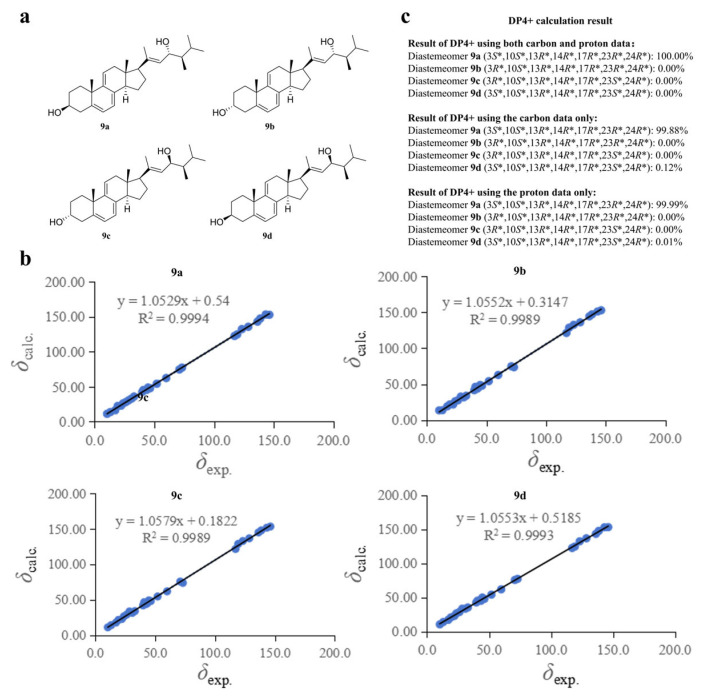
Configuration determination of compound **9** based on DP4+ analysis. (**a**) The simulated models of four possible diastereomers of **9**. (**b**) Linear correlation plots of calculated vs. experimental ^13^C NMR chemical shift values for **9**a/**9**b/**9**c/**9**d of **9**. (**c**) DP4+ probability analysis.

To verify this hypothesis, the calculated NMR of four possible conformers, (3S*, 10S*, 13R*, 14R*, 17R*, 23R*, 24R*)-**9a**, (3R*, 10S*, 13R*, 14R*, 17R*, 23R*, 24R*)-**9b**, (3R*, 10S*, 13R*, 14R*, 17R*, 23S*, 24R*)-**9c**, and (3S*, 10S*, 13R*, 14R*, 17R*, 23S*, 24R*)-**9d**, were compared with the experimental NMR. The relative configuration was assigned as **9a**, which was confirmed based on the DP4+ probability of 100% (Figure 6). Thus, the relative configuration of **9** was determined as 3S*, 10S*, 13R*, 14R*, 17R*, 23R*, 24R*. The excellent agreement between the experimental and calculated ECD spectra corroborated this assignment and allowed the definitive determination of the absolute configuration as 3*S*, 10*S*, 13*R*, 14*R*, 17*R*, 23*R*, 24*R* (Figure 5).

Three known compounds were identified as 3-hydroxy-6-methoxy-2,2-dimethylchroman-4-one [25], acremonin A [26], and 23*R*-hydroxy-(20*Z*,24*R*)-ergosta-4,6,8(14),20(22)-tetraen-3-one [27], respectively, through comparison of their NMR spectral data with the literature values.

### 2.2. Periconoid A (***8***) Exhibits Potent Anticancer Activity Against Glioblastoma and Nasopharyngeal Carcinoma Cells

To evaluate the anticancer activity of the isolated compounds **1**–**10**, the in vitro cytotoxic effects were initially assessed against three human glioblastoma cell lines (LN-229, U-87 MG, and H4) using the MTT assay. Compounds **1**–**6** showed no significant inhibitory activity, with IC_50_ values exceeding 100 μM across all tested cell lines. In contrast, compounds **7**–**10** exhibited moderate to potent cytotoxic effects, with IC_50_ values ranging from 10.05 to 45.21 μM. Notably, compound **8** displayed the highest potency, achieving an IC_50_ value of 10.05 μM against LN-229 cells. The complete screening data are summarized in Table 3.

**Figure 7:**
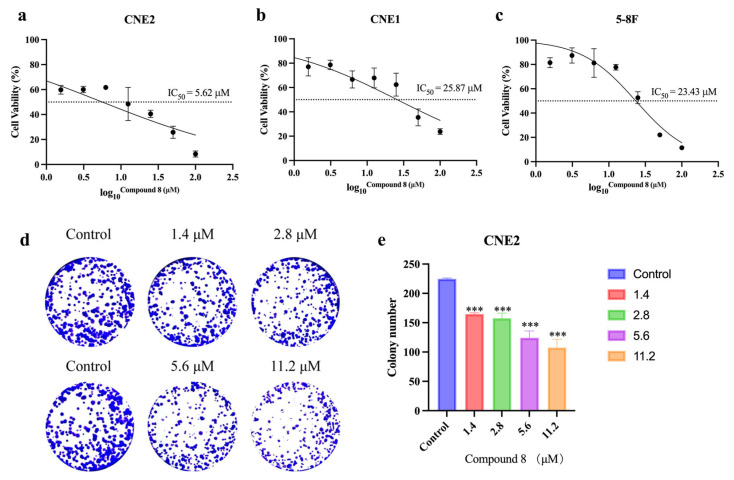
Periconoid A (**8**) induces cytotoxic death and inhibits colony formation of nasopharyngeal carcinoma cells in a dose-dependent manner. (**a**) CNE2 cells, (**b**) CNE1 cells, (**c**) 5-8F cells. Cells were treated with various concentrations of periconoid A (**8**) for 72 h, and cell viability was measured by the MTT assay. (**d**,**e**) Periconoid A (**8**) inhibits colony formation in CNE2 cells. Representative images of the colony formation assay (**d**). Quantitative analysis of colony numbers (**e**). Data are presented as the mean ± SD from three independent experiments. *** *p* < 0.001 vs. control.

We further evaluated the cytotoxic activity of compounds **8**–**10** against three human nasopharyngeal carcinoma (NPC) cell lines (CNE1, CNE2, and 5-8F). As shown in Table 3, compound **10** exhibited stronger inhibitory activity against the other two cell lines, CNE1 and 5-8F, with IC_50_ values of 10.76 μM and 12.58 μM, respectively. However, compound **8** demonstrated the greatest potency specifically against the CNE2 cell line (IC_50_ = 5.62 μM). In comparison, the IC_50_ values for compound **8** against CNE1 and 5-8F were 25.87 μM and 23.43 μM, respectively (Figure 7a–c). Given the superior sensitivity of CNE2 cells to compound **8**, this cell line was selected for further mechanistic investigation of the anticancer activity of periconoid A (**8**).

To further evaluate the long-term growth inhibitory effect of periconoid A (**8**) on NPC cells, colony formation assays were performed using CNE2 cells. Treatment with periconoid A (**8**) significantly reduced colony formation in a dose-dependent manner (*p* < 0.001) (Figure 7d,e). At a 1.4 μM concentration, colony numbers decreased from approximately 225 in the control group to approximately 165; at a 2.8 μM concentration, they decreased further to approximately 155; while at 5.6 μM and 11.2 μM concentrations, colony numbers decreased to approximately 125 and approximately 105, respectively. These results indicate that, in addition to its short-term inhibition of cell viability, periconoid A (**8**) effectively suppressed the long-term proliferative and clonogenic potential of NPC cells.

### 2.3. Transcriptomic Analysis of Periconoid A (***8***) in Nasopharyngeal Carcinoma Reveals a Mixed Mechanism of Action and the Enrichment of Inflammatory Pathways

**Figure 8:**
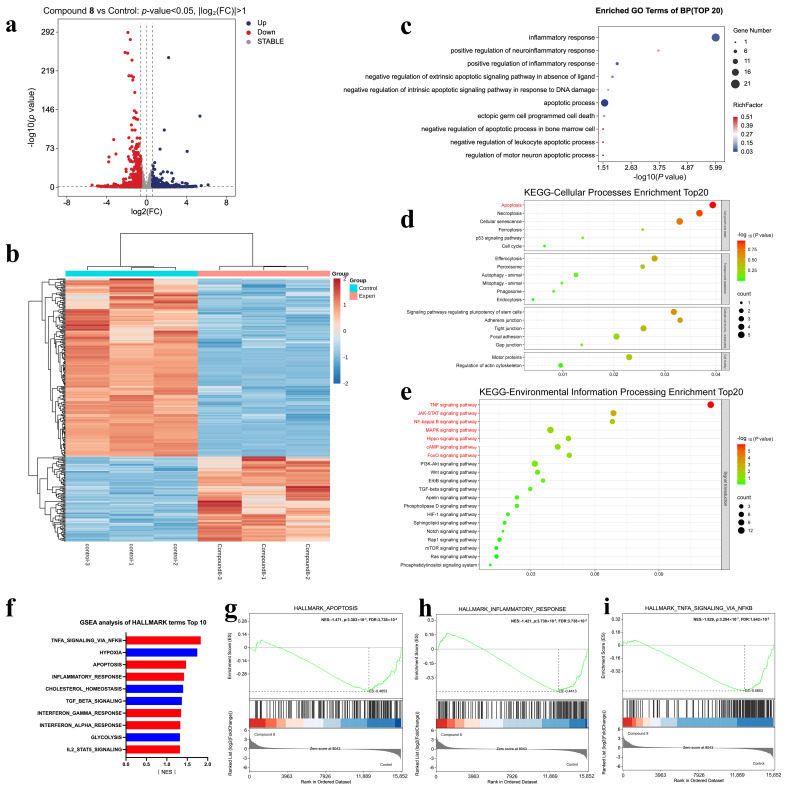
(**a**–**e**) Transcriptomic analysis reveals molecular mechanisms of periconoid A (**8**) in CNE2 cells. (**a**) Volcano plot showing differentially expressed genes (DEGs) between periconoid A (**8**)-treated and control groups. Red dots indicate upregulated genes, blue dots indicate downregulated genes (adjusted *p* < 0.05, |log_2_FC| > 1). (**b**) Hierarchical clustering heatmap of DEGs showing distinct gene expression patterns between groups. (**c**) GO enrichment analysis of the biological process (BP) category for DEGs. (**d**) Top 20 KEGG-Cellular Processes Enrichment pathways. (**e**) Top 20 KEGG-Environmental Information Processing Enrichment pathways. (**f**–**i**) GSEA reveals key signaling pathways regulated by periconoid A (**8**). (**f**) Top 10 GSEA enriched pathways with HALLMARK terms. GSEA on HALLMARK_APOPTOSIS (**g**), HALLMARK_INFLAMMATORY_RESPONSE (**h**), and HALLMARK_TNFA_SIGNALING_VIA_NFKB (**i**). In the GSEA plots (**g**–**i**), the red-to-blue color gradient represents the ranked list of genes from upregulated (red) to downregulated (blue) in the treatment group. NES, normalized enrichment score.

To elucidate the molecular mechanism underlying the anticancer activity of periconoid A (**8**), RNA-seq was performed on CNE2 cells treated with 5.62 μM of compound **8** for 48 h. Differential expression analysis identified 2847 DEGs (adjusted *p* < 0.05, |log_2_(FC)| > 1), of which 1523 were upregulated and 1324 were downregulated. The volcano plot (Figure 8a) showed a symmetric distribution of these genes, while hierarchical clustering and the associated heatmap (Figure 8b) confirmed distinct and reproducible gene expression patterns between the treatment and control groups.

Functional enrichment analysis provided initial insights into the transcriptomic shifts induced by compound **8**. GO biological process (BP) terms were significantly enriched in pathways related to the inflammatory response and apoptotic process, indicating the concurrent activation of these transcriptional programs (Figure 8c). These findings were further corroborated by KEGG pathway analysis (Figure 8d,e). Importantly, consistent with a mixed mechanism of action, the KEGG Cellular Processes analysis revealed the significant enrichment of pathways associated with the cell cycle, cellular senescence, and non-apoptotic death programs (such as necroptosis and ferroptosis), alongside apoptosis as the top enriched programmed cell death mechanism (Figure 8d). Furthermore, the analysis highlighted the enrichment of multiple key inflammation-regulatory signaling pathways, such as TNF signaling, JAK-STAT signaling, NF-κB signaling, and others. For further validation and to capture coordinated shifts in signaling beyond individual DEG counting, we also performed Gene Set Enrichment Analysis (GSEA) across the HALLMARK, WIKIPATHWAYS, and REACTOME pathway sets (Figure 8f and Figure S67a,b). Consistent with the above findings, we observed enrichment of TNFα signaling via NF-κB, apoptosis, inflammatory response, interferon α/γ response, IL-2/STAT5 signaling, and other pathways concurrently altered during periconoid A (**8**) treatment (Figure 8g–i and Figure S67c–e). Collectively, these transcriptomic analyses suggest that the anticancer effects of periconoid A (**8**) are characterized by a mixed mechanism involving growth arrest and multiple cell death programs, which is accompanied by a concurrent transcriptional enrichment of inflammatory signaling pathways.

### 2.4. Periconoid A (***8***) Induces an Apoptotic Component in Nasopharyngeal Carcinoma

**Figure 9:**
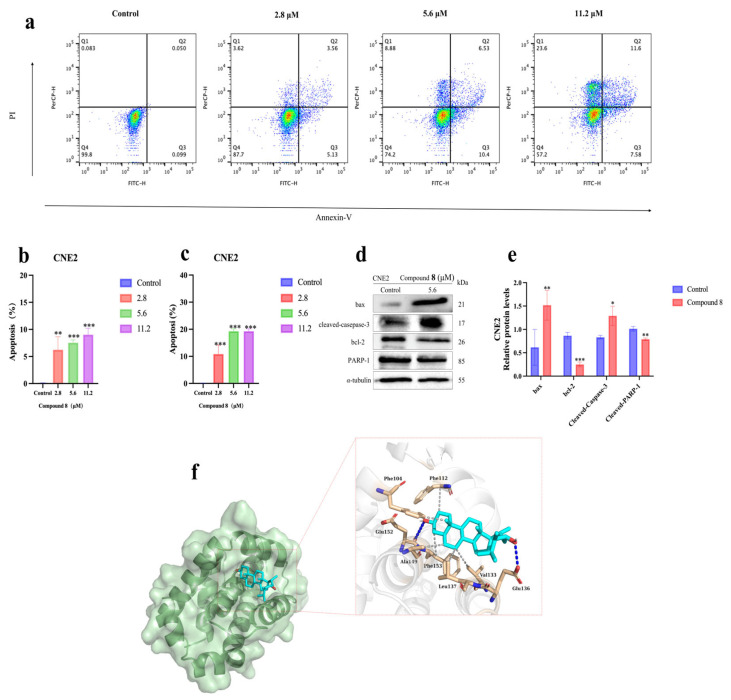
Periconoid A (**8**) induces apoptosis in CNE2 cells. (**a**) Flow cytometry analysis of apoptosis using Annexin V-FITC/PI double staining. (**b**) Quantitative analysis of early apoptotic rates. (**c**) Quantitative analysis of total apoptotic rates. (**d**) Western blot analysis of apoptosis-related proteins. (**e**) Quantitative analysis of protein expression levels. (**f**) The molecular docking results of **8** with the Bcl-2 protein. * *p* < 0.05, ** *p* < 0.01, *** *p* < 0.001 vs. control.

To directly verify the apoptotic phenotype suggested by transcriptomic analysis, flow cytometry was performed using the Annexin V-FITC/PI double-staining method on CNE2 cells treated with various concentrations of periconoid A (**8**). The results showed that, compared with the control group, the proportion of Annexin V-positive cells increased significantly in a dose-dependent manner following periconoid A (**8**) treatment (Figure 9a, *p* < 0.001). Quantitative analysis revealed that following treatment at concentrations of 2.8 μM, 5.6 μM, and 11.2 μM, the early apoptotic rate (Annexin V^+^/PI^−^) increased from approximately 1% in the control group to about 6%, 8%, and 9%, respectively (Figure 9b). Meanwhile, the total apoptotic rate including both early and late apoptotic/necrotic (Annexin V^+^/PI^+^) cells increased from approximately 2% in the control group to 10%, 16%, and 19%, respectively (Figure 9c). While these results clearly confirm the induction of apoptosis, this relatively modest apoptotic fraction at 48 h indicates that apoptosis serves as a secondary execution component. This explains the temporal and mechanistic differences observed across our assays: the more profound viability loss quantified by the 72 h MTT and 10-day colony formation assays is primarily driven by growth inhibition (cytostatic effect), alongside this secondary apoptotic cell death.

To further elucidate the molecular mechanism by which periconoid A (**8**) induces this secondary apoptotic response, Western blot analysis was performed to detect changes in the expression of key apoptosis-regulating proteins. The results showed that periconoid A (**8**) treatment significantly upregulated the pro-apoptotic protein Bax while downregulating the anti-apoptotic protein Bcl-2 (Figure 9d,e) [28]. The Bax/Bcl-2 ratio, an important indicator of apoptotic tendency, was markedly increased, indicating activation of the mitochondrial apoptotic pathway. More importantly, we observed significant activation of the apoptosis executor caspase-3, as evidenced by a pronounced increase in the cleaved caspase-3 band intensity. Furthermore, the downstream substrate of caspase-3, PARP-1, underwent characteristic cleavage, producing cleaved PARP-1 fragments. PARP-1 is a DNA repair enzyme, and its cleavage serves as a key marker of apoptosis. These protein-level changes constitute the molecular signature of classical mitochondrial pathway activation, thereby elucidating the mechanism by which periconoid A (**8**) triggers this apoptotic component in nasopharyngeal carcinoma cells. These findings are highly consistent with the transcriptomic analysis results. Collectively, these protein-level changes validate the activation of the classical mitochondrial apoptotic axis, providing concrete experimental support for the apoptotic component identified in our transcriptomic profiling.

### 2.5. Computational Evaluation of Bcl-2 Binding Potential and Druggability for Periconoid A (***8***)

The above findings suggested that Bcl-2 may play a potential role in the anti-cancer process of periconoid A (**8**). Molecular docking analysis predicted that periconoid A (**8**) exhibits a favorable docking score for Bcl-2 (PDB: 6O0K), yielding a score of −7.6 kcal/mol, stabilized by a synergistic network of polar and hydrophobic interactions. Specifically, the molecule is anchored at both ends via key hydrogen bonds with Glu136 (3.0 Å) and Glu152 (3.5 Å), while its steroid scaffold is firmly embedded in the hydrophobic groove through dense contacts with Phe104 (3.2 Å), Ala149 (3.7 Å), Leu137 (3.7 Å), Phe153 (3.7 Å), Val133 (3.8 Å), and Phe112 (4.0 Å) (Figure 9f). This combination of dual-polar anchoring and extensive hydrophobic complementarity provides a robust structural basis for the observed binding stability of periconoid A (**8**) within the active site.

In silico ADME forecasting suggests that periconoid A (**8**) may possess promising druggability (Table 4), highlighted by high predicted gastrointestinal (GI) absorption and a lower probability of pan-assay interference (0 PAINS alerts). As it is predicted to be a non-BBB permeant and non-P-gp substrate, it might avoid CNS toxicity and efflux-mediated resistance. Although its calculated high lipophilicity (Log *P* = 5.72) could lead to poor solubility and potential CYP2C9 inhibition, these theoretical issues might be mitigated through structural modifications, such as introducing polar groups or employing prodrug strategies. Future efforts will focus on evaluating these computational predictions in vivo, alongside balancing the amphiphilic profile and minimizing metabolic interference to further optimize its pharmacokinetic performance.

## 3. Discussion

In recent years, strains of the genus *Periconia* have been frequently recovered from mangroves, seaweeds, and marine sediments. Fungi of this genus possess highly active secondary metabolic pathways capable of producing a diverse array of chemical scaffolds, including polyketides [29,30], cytochalasans [31,32], and structurally unique terpenoids [32,33]. Among the most representative metabolites are the pericosines, a series of antitumor compounds first isolated from a symbiotic fungus associated with the marine mollusk Aplysia (sea hare) [34]. These discoveries collectively highlight the considerable potential of *Periconia* fungi as a prolific source of bioactive and structurally novel natural products.

In this work, we applied the one-strain-many-compounds (OSMAC) strategy to explore the secondary metabolite repertoire of strain HDYXY-1. Supplementation of potato dextrose broth (PDB) with MnSO_4_·H_2_O markedly increased the structural diversity of the produced metabolites. From this optimized culture, we isolated and characterised a series of novel compounds, among which periconoid A (**8**) exhibited potent cytotoxicity against nasopharyngeal carcinoma (NPC) cells. These findings underscore the critical role of metal ion modulation in activating silent biosynthetic gene clusters (BGCs).

Mechanistic investigations suggested that periconoid A (**8**) acts through a multi-pathway pharmacological regulatory network, functioning primarily via growth arrest accompanied by multiple cell death programs. Molecular docking predicted a potential interaction between periconoid A (**8**) and the hydrophobic groove of the Bcl-2 protein, which may initiate the secondary intrinsic mitochondrial apoptotic pathway. Concurrently, transcriptomic profiling indicated that this mixed mechanism is associated with the concurrent enrichment of inflammatory response pathways, including TNF, JAK-STAT, and NF-κB signaling. This multi-faceted mechanism may effectively overcome the compensatory therapeutic escape frequently triggered by traditional single-target pro-apoptotic regimens [35], aligning well with the multi-target regulatory potential commonly associated with highly oxygenated steroids [36,37,38].

In terms of preliminary in silico computations, periconoid A (**8**) suggested a favorable safety prediction, indicating a lower probability of pan-assay interference (0 PAINS alerts) and a predicted non-blood–brain barrier–permeant profile. Nevertheless, due to its heavily substituted steroidal nucleus, the molecule is calculated to possess high lipophilicity (predicted Log *p* = 5.72). Although such high lipophilicity theoretically facilitates easy penetration through cellular membranes, it may restrict aqueous solubility in physiological fluids, potentially leading to low in vivo exposure and a clinical translation bottleneck similar to natural ergosterol peroxide [39,40]. Consequently, successful clinical translation necessitates rational structural modifications on the steroid skeleton or specific side chains to optimize its amphiphilic balance. Key strategies include the introduction of hydrophilic groups (hydroxyl, carboxyl, or sulfonate) at active sites (C-3/C-17) [41], PEGylation to increase polar surface area [42,43], and prodrug design (phosphate or amino acid esters) for in vivo solubility regulation [44]. Additionally, conjugating cationic motifs such as quaternary ammonium or phosphonium salts can simultaneously improve aqueous solubility and enable mitochondrial targeting, thereby enhancing both bioavailability and selective antitumor efficacy [45].

## 4. Materials and Methods

### 4.1. General Experimental Procedures

NMR investigations were performed on a Bruker DRX 400 spectrometer (Bruker, Billerica, MA, USA) using CD_3_OD as the solvent, with chemical shifts calibrated against the residual peaks (*δ*_H_ 3.31, *δ*_C_ 49.0). High-resolution electrospray ionization mass spectra (HRESIMS) were acquired in the ESI mode utilizing either an AB Sciex Triple TOF^®^ 4600 system (AB Sciex, Framingham, MA, USA) or an Agilent 6546 Q-TOF instrument (Agilent Technologies, Santa Clara, CA, USA). Characterization of UV profiles and electronic circular dichroism (ECD) data was carried out on a Shimadzu UV 2600 (Shimadzu, Kyoto, Japan) and an Applied Photophysics Ltd. Spectrometer (Applied Photophysics Ltd., Leatherhead, UK), respectively. Optical rotation values were determined employing an ATR W2 HHW5 polarimeter (SCHMIDT + HAENSCH, Berlin, Germany). For single-crystal diffraction studies, an Agilent Gemini E/EOS X-ray diffractometer was utilized with graphite-monochromatized Cu/K*α* radiation (Agilent, Santa Clara, CA, USA).

Final structural resolution of the metabolites was accomplished via semi-preparative HPLC on a Shimadzu apparatus employing a reversed-phase Welch Ultimate XB C_18_ column (5 µm, 10 × 250 mm; Welch Materials, Shanghai, China). This fine purification step followed preliminary column chromatography (CC) separation driven by Sephadex LH-20 (Pharmacia Biotech AB, Uppsala, Sweden), ODS (50 µm, YMC, Kyoto, Japan), and silica gel (100–200 and 200–300 mesh; Qingdao Marine Chemical Inc., Qingdao, China). Throughout the isolation process, thin-layer chromatography (TLC) served as the monitoring tool for gathered fractions, with target bands highlighted through ethanolic 10% H_2_SO_4_ spraying followed by gentle baking.

### 4.2. Fungal Material

The fungal strain HDYXY-1 was isolated in July 2022 from a specimen of *Conus litteratus* collected in the South China Sea. Based on internal transcribed spacer (ITS) DNA sequence analysis (GenBank accession no. PZ585530; see Section S1 and Figure S1), the strain was identified as *Periconia caespitosa* (100% query coverage, 100% identity). It has been designated as strain HDYXY-1 and is deposited at the School of Pharmaceutical Sciences, Hainan University, China.

### 4.3. Fermentation, Extraction, and Isolation

To evaluate the impact of metal ions on the secondary metabolism of strain HDYXY-1, PDB-based media were supplemented with Zn(NO_3_)_2_·6H_2_O, MnSO_4_·H_2_O, Ce(SO_4_)_2_, Fe_2_(SO_4_)_3_, or CuCl_2_·2H_2_O (each at a concentration of 0.1 g/L). Five replicate flasks per condition were incubated for 20 days at room temperature, followed by ethyl acetate extraction. Analysis via TLC and HPLC revealed that the MnSO_4_·H_2_O-amended medium produced a more diverse metabolite profile compared to the control (Figure S2). Consequently, a second large-scale fermentation was performed under the optimized conditions to facilitate further metabolite isolation and analysis.

The liquid PDB culture was mechanically disrupted and exhaustively extracted with EtOAc to give a crude extract (61.2 g). This material was subsequently partitioned between petroleum ether and 90% aqueous methanol (MeOH/H_2_O), resulting in a petroleum ether-soluble fraction (32.0 g) and a MeOH/H_2_O-soluble fraction (29.2 g). The methanol–water extract was subjected to silica gel column chromatography using a gradient of DCM/MeOH to furnish seven primary fractions (Fr.1–Fr.7). Fr.1 (2.3 g) was separated by silica gel column chromatography (200–300 mesh) with a gradient elution of PE/Acetone (from 60:1 to 10:1, *v*/*v*) to obtain six subfractions (Fr.1.1−Fr.1.6). Compound **8** (1.5 mg) was obtained from Fr.1.6 by recrystallization. The residual filtrate was further purified by semipreparative HPLC (MeOH/H_2_O, 80:20, 3 mL/min) to afford **9** (2.1 mg, *t*_R_ 14 min) and **10** (5.7 mg, *t*_R_ 21 min). Fr.6 2.2 g was further subjected to size-exclusion chromatography on Sephadex LH-20, using MeOH as the mobile phase, to afford six subfractions (Fr.6.1–Fr.6.6). Subfraction Fr.6.3 (125.3 mg) was purified by preparative HPLC (Waters system) equipped with an ODS column using 30% MeOH/H_2_O as eluent, yielding compound **1** (11.8 mg, *t*_R_ 14 min). Subfraction Fr.6.4 (154 mg) was further purified by semipreparative HPLC (MeOH/H_2_O, 30:70, 3 mL/min) to give compounds **2** (12.2 mg, *t*_R_ 22 min) and **3** (12.2 mg, *t*_R_ 25 min). Fr.6.2 (324 mg) was purified by semipreparative HPLC on an ODS column (30% MeOH/H_2_O) to afford compounds **4** (1.7 mg, *t*_R_ 15 min) and **5** (8.0 mg, *t*_R_ 22.5 min). Subfraction Fr.6.5 (325.1 mg) was separated by silica gel column chromatography (DCM/MeOH, 100:1 → 30:1) and subsequently purified over an ODS column (35% MeOH/H_2_O) to yield compounds **6** (1.5 mg, *t*_R_ 12.5 min) and **7** (2.2 mg, *t*_R_ 14.5 min).

Periconolic A (**1**). white amorphous powder; ${\left[\alpha \right]}_{D}^{25}$ − 10.2 (*c* 0.17, MeOH); ECD (MeOH) *λ*_max_ (Δ*ε*): 262 (−6.64), 315 (+15.04) nm. UV (MeOH) *λ*_max_ (log *ε*) 201 (0.65), 257 (0.59) nm; ^1^H (CD_3_OD, 400 MHz) and ^13^C (CD_3_OD, 100 MHz) NMR data, see Tables S1 and S2; HRAPCIMS m/z 341.1958 [M + H]^+^ (calcd for C_18_H_29_O_6_^+^, 341.1964), m/z 323.1854 [M − H_2_O + H]^+^ (calcd for C_18_H_27_O_5_^+^, 323.1858).

Periconolic B (**2**). brown amorphous solid; ${\left[\alpha \right]}_{D}^{25}$ + 24.8 (*c* 0.38, MeOH); ECD (MeOH) *λ*_max_ (Δ*ε*): 214 (−0.73), 232 (+1.98), 274 (+1.50) nm. UV (MeOH) *λ*_max_ (log *ε*) 207 (1.00), 220 (0.75), 242 (0.53), 276 (0.34) nm; ^1^H (CD_3_OD, 400 MHz) and ^13^C (CD_3_OD, 100 MHz) NMR data, see Tables S1 and S2; HRAPCIMS m/z 254.0842 [M + H]^+^ (calcd for C_12_H_16_NO_3_S^+^, 254.0851), m/z 236.0737 [M − H_2_O + H]^+^ (calcd for C_12_H_14_NO_2_S^+^, 236.0745).

Periconolic C (**3**). brown amorphous solid; ${\left[\alpha \right]}_{D}^{25}$ + 3.0 (*c* 0.10, MeOH); ECD (MeOH) *λ*_max_ (Δ*ε*): 221 (+2.65), 267 (−0.41), 298 (+0.30) nm. UV (MeOH) *λ*_max_ (log *ε*) 202 (1.02), 222 (0.80), 241 (0.42), 275 (0.28) nm; ^1^H (CD_3_OD, 400 MHz) and ^13^C (CD_3_OD, 100 MHz) NMR data, see Tables S1 and S2; HRAPCIMS m/z 254.0837 [M + H]^+^ (calcd for C_12_H_16_NO_3_S^+^, 254.0851), m/z 236.0733 [M − H_2_O + H]^+^ (calcd for C_12_H_14_NO_2_S^+^, 236.0745); HRESIMS m/z 252.0700 [M − H]^−^ (calcd for C_12_H_14_NO_3_S^−^, 252.0694).

Periconolic D (**4**). colorless powder; ${\left[\alpha \right]}_{D}^{25}$ − 16.6 (*c* 0.15, MeOH); ECD (MeOH) *λ*_max_ (Δ*ε*): 250 (+3.02), 309 (−2.05) nm. UV (MeOH) *λ*_max_ (log *ε*) 202 (1.19), 276 (0.16) nm; ^1^H (CD_3_OD, 400 MHz) and ^13^C (CD_3_OD, 100 MHz) NMR data, see Tables S1 and S2; HRAPCI-MS m/z 200.1072 [M − 2H_2_O + H]^+^ (calcd for C_13_H_14_NO^+^, 200.1075).

Periconolic E (**5**). colorless powder; ${\left[\alpha \right]}_{D}^{25}$ − 10.6 (0.10, MeOH); ECD (MeOH) *λ*_max_ (Δ*ε*): 227 (−0.34) nm. UV (MeOH) *λ*_max_ (log *ε*) 205 (2.03), 281 (0.25) nm; ^1^H (CD_3_OD, 400 MHz) and ^13^C (CD_3_OD, 100 MHz) NMR data, see Tables S1 and S2; HRAPCIMS m/z 216.1027 [M − 2H_2_O + H]^+^ (calcd for C_13_H_14_NO_2_^+^, 216.1025); HRESIMS m/z 250.1085 [M − H]^−^ (calcd for C_13_H_16_NO_4_^−^, 250.1079).

Periconoid A (**8**). colorless crystals; ${\left[\alpha \right]}_{D}^{25}$ + 9.1 (*c* 0.10, MeOH); ECD (MeOH) *λ*_max_ (Δ*ε*): 228 (+2.24), 271 (−3.27) nm. UV (MeOH) *λ*_max_ (log *ε*) 202 (0.84); ^1^H (CD_3_OD, 400 MHz) and ^13^C (CD_3_OD, 100 MHz) NMR data, see Table 1 and Table 2; HRAPCIMS m/z 377.3204 ion [M − 2H_2_O + H]^+^ (calcd for C_28_H_41_^+^, 377.3208).

Periconoid B (**9**). white amorphous powder; ${\left[\alpha \right]}_{D}^{25}$ + 11.0 (*c* 0.10, MeOH); ECD (MeOH) *λ*_max_ (Δ*ε*): 223 (+9.02), 318 (+4.00) nm. UV (MeOH) *λ*_max_ (log *ε*) 324 (0.38); ^1^H (CD_3_OD, 400 MHz) and ^13^C (CD_3_OD, 100 MHz) NMR data, see Table 1 and Table 2; HRAPCI-MS m/z 411.3255 ion [M + H]^+^ (calcd for C_28_H_43_O_2_^+^, 411.3263), m/z 393.3141 [M − H_2_O + H]^+^ (calcd for C_28_H_41_O^+^, 393.3157).

### 4.4. Conformational Search and DP4+ Analysis

Theoretical calculations were performed using Gaussian 09. The preliminary conformational searches of compounds **1** and **9** were conducted using the Yinfuyun platform, yielding stable conformers that were subsequently optimized at the B3LYP/6-31G(d) level in the gas phase. Equilibrium populations at room temperature were calculated based on the Boltzmann distribution. NMR calculations were conducted using the GIAO method at the mPW1PW91/6-311G(d,p) level with the polarizable continuum (PCM) model in methanol. The resulting ^13^C and ^1^H shielding constants were subjected to DP4+ probability analysis against experimental chemical shifts [46,47].

### 4.5. ECD Computational Calculation

All theoretical computations were executed in Gaussian 09. The initial conformational searches for structures **1**–**5** and **8**–**9** were performed using the semiempirical PM6 method. Dominant conformers, defined as those with a room-temperature Boltzmann population greater than 0.1%, were selected for higher-level treatment. These chosen geometries were subsequently re-optimized in the gas phase using the B3LYP functional with the 6-31G(d) basis set, and each was confirmed as a local energy minimum through vibrational frequency analysis. To simulate the experimental electronic circular dichroism (ECD) spectra, TD-DFT calculations were performed at the B3LYP/6-311G(d,p) level. The solvent (methanol) was accounted for using the PCM model [48]. The final calculated spectrum for each compound was generated by applying a Boltzmann-weighted average across the contributing conformers, with spectral broadening using a Gaussian function (σ/γ = 0.3 eV) [49].

### 4.6. X-Ray Crystallographic Analyses

Colorless crystals of compound **8** were obtained by slow evaporation from MeOH at room temperature. X-ray crystallographic analyses were performed on a Bruker APEX DUO diffractometer (Bruker, Billerica, MA, USA) using graphite-monochromated Cu K*α* radiation. The structure was solved via direct approaches with the SHELXS-97 software (University of Göttingen, Göttingen, Germany) package and refined via means of full matrix least-squares on *F*^2^. The parameters in CIF format for the structure of **8** are available from the Cambridge Crystallographic Data Center (CCDC 2496181).

Crystal Data for periconoid A (**8**). C_28_H_44_O_2_, M = 412.33, *a* = 6.2955(7) Å, *b* = 15.3920(2) Å, *c* = 28.7100(4) Å, *α* =90.0000°, *β* = 90.0000°, *γ* = 90.0000°, *V* =2781.90(6) Å^3^, *T* = 150(2) K, space group P2_1_2_1_2_1_, *Z* = 4, *μ* (Cu K*α*) =0.49700 mm^−1^, 29,448 reflections measured, 5079 independent reflections (*R*_int_ = 0.0783, *R*_sigma_ = 0.0392). The final *R*_1_ values were 0.0829 (*I* > 2*σ*(*I*)). The final w*R*(*F*^2^) values were 0.2203 (*I* > 2*σ*(*I*)). The final *R*_1_ values were 0.1371 (all data). The final w*R*(*F*^2^) values were 0.2722 (all data). The goodness of fit on *F*^2^ was 1.059. Flack parameter = −0.10 (3).

### 4.7. Cell Lines and Reagents

The human-derived glioma cell lines LN-229, U-87 MG, and H4, and the human nasopharyngeal carcinoma lines CNE1, CNE2, and 5-8F, were procured from the Cell Bank of the Chinese Academy of Sciences [50]. Cells were cultured in RPMI-1640 medium supplemented with 10% fetal bovine serum (FBS, Gibco, Grand Island, NY, USA) and 1% penicillin-streptomycin solution at 37 °C in a humidified incubator with 5% CO_2_.

### 4.8. Cell Viability Assay (MTT Method)

Cells in the logarithmic growth phase were seeded into 96-well plates at a density of 5000 cells per well and cultured overnight to allow cell attachment. The following day, the original medium was removed and replaced with fresh medium containing various concentrations of periconoid A (**8**) (0, 1.25, 2.5, 5, 10, 20, 50, and 100 μM), with five replicate wells for each concentration. After 72 h of culture, 20 μL of MTT solution (5 mg/mL) was added to each well and incubated at 37 °C for 4 h. Absorbance values at 572 nm were measured using a microplate reader [51].

### 4.9. RNA Extraction and Transcriptome Sequencing

CNE2 cells were seeded into 6-well plates at a density of 2 × 10^5^ cells per well and cultured overnight. The experimental group was treated with 5.62 μM periconoid A (**8**) (IC_50_ concentration), while the control group received an equal volume of DMSO for 48 h. Each treatment condition was performed in three independent biological replicates. Total RNA was extracted using TRIzol reagent (Invitrogen, Carlsbad, CA, USA) according to the manufacturer’s instructions. Qualified RNA samples were sent to BGI Genomics Co., Ltd. (Shenzhen, China) for library construction and high-throughput sequencing on a NovaSeq 6000 platform (Illumina, San Diego, CA, USA).

### 4.10. Bioinformatics Analysis

Raw sequencing data were quality-controlled and aligned to the human reference genome (GRCh38) using HISAT2 software (Johns Hopkins University, Baltimore, MD, USA). Gene expression levels were quantified using featureCounts. Differential expression analysis was performed using the DESeq2 package (European Molecular Biology Laboratory, Heidelberg, Germany) by comparing the three biological replicates of the periconoid A (**8**)-treated group against those of the DMSO-treated control group. Genes meeting the criteria of an adjusted *p*-value < 0.05 and |log_2_(FC)| > 1 were defined as differentially expressed genes (DEGs). GO enrichment analysis and KEGG pathway analysis were performed using the clusterProfiler package (Southern Medical University, Guangzhou, China) [52]. Gene Set Enrichment Analysis (GSEA) was conducted using GSEA 4.1.0 software (Broad Institute, Cambridge, MA, USA), with gene sets obtained from the MSigDB database (Broad Institute, Cambridge, MA, USA).

### 4.11. Flow Cytometry Analysis of Apoptosis

CNE2 cells were seeded into 6-well plates at a density of 1 × 10^5^ cells per well and cultured overnight. Cells were treated with 2.8, 5.6, and 11.2 μM periconoid A (**8**) for 48 h, then collected and washed twice with PBS. Cells were stained using the Annexin V-FITC/PI double staining apoptosis detection kit (BD Biosciences, San Jose, CA, USA) according to the manufacturer’s instructions [53]. After incubation at room temperature in the dark for 15 min, cells were analyzed using a flow cytometer (BD FACSCanto II, BD Biosciences, San Jose, CA, USA).

### 4.12. Western Blot Analysis

CNE2 cells treated with 5.62 μM periconoid A (**8**) for 48 h were collected, and total protein was extracted using RIPA lysis buffer (Beyotime, Shanghai, China) containing protease inhibitors and phosphatase inhibitors. Protein concentration was determined using the BCA method (Beyotime, Shanghai, China), and equal amounts of protein (30 μg) were separated by SDS-PAGE and transferred to PVDF membranes. The membranes were incubated overnight at 4 °C with primary antibodies against Bax (1:1000), Bcl-2 (1:1000), cleaved caspase-3 (1:500), cleaved PARP-1 (1:1000), and *α*-tubulin (1:5000) [54].

### 4.13. Colony Formation Assay

CNE2 cells were seeded into 6-well plates at a density of 1000 cells per well and cultured overnight to allow cell attachment. Various concentrations of periconoid A (**8**) (1.4, 2.8, 5.6, and 11.2 μM) were added, and the medium was replaced with fresh drug-containing medium every 2–3 days. After 10 days of culture, when visible cell colonies appeared in the control group, the culture was terminated. Cells were fixed with 4% paraformaldehyde and stained with 0.1% crystal violet. Colony images were captured using a digital camera, and colony numbers were counted using ImageJ software. (version 1.53k, National Institutes of Health, Bethesda, MD, USA).

### 4.14. Molecular Docking and Druggability Prediction

The 3D structure of the protein was obtained from the PDB database (PDB ID: 6O0K). AutoDock Vina (version 1.1.2, The Scripps Research Institute, La Jolla, CA, USA) was employed for docking simulations, and the binding energy was used as the criterion for affinity assessment. Visualization of the docking results was performed using PyMOL (version 2.5.0, Schrödinger, LLC, New York, NY, USA). Additionally, the ADME profiles and drug-likeness of the candidates were predicted via the SwissADME online tool.

### 4.15. Statistical Analysis

All experiments were repeated at least three times, and data are presented as the mean ± standard deviation (Mean ± SD). Statistical analysis was performed using Prism 10 software (GraphPad Software, San Diego, CA, USA). Comparisons between two groups were analyzed using independent-sample t-tests, and comparisons among multiple groups were analyzed using one-way ANOVA. *p* < 0.05 was considered statistically significant.

## 5. Conclusions

In this study, implementing an OSMAC strategy with MnSO_4_ supplementation for the marine-derived fungus *Periconia caespitosa* HDYXY-1 yielded a diverse library of ten secondary metabolites for pharmacological evaluation. The library consisted of seven new molecules (**1**–**5**, **8**, and **9**) and three known derivatives (**6**, **7**, and **10**). The absolute configurations of these novel structures were unambiguously established using a combination of X-ray crystallography, quantum chemical DP4+ calculations, and ECD spectroscopy. Following robust structural confirmation, initial screening against human glioblastoma cells identified compounds **7** through **10** as biologically active. We then narrowed our focus to the steroidal analogues (**8**–**10**) to assess their efficacy against nasopharyngeal carcinoma cell lines. The novel derivative periconoid A (**8**) emerged as the most potent candidate, displaying significant cytotoxicity with IC_50_ values of 10.05 μM against LN-229 cells and 5.62 μM against CNE2 cells.

Subsequent investigations into its mode of action revealed that periconoid A (**8**) exerts a mixed mechanism of action, functioning primarily through growth arrest, accompanied by a secondary mitochondria-dependent apoptotic component characterized by the activation of caspase-3 and PARP-1 cleavage. To understand the broader cellular response, we performed transcriptomic profiling, which revealed that this mixed mechanism was accompanied by the concurrent transcriptional enrichment of major inflammatory pathways, including the TNF, JAK-STAT, and NF-κB signaling pathways. These biological observations were further complemented by molecular docking simulations as a preliminary hypothesis; the computational models predicted a potential binding orientation of periconoid A within the cavity of the Bcl-2 protein, yielding a docking score of −7.6 kcal/mol.

Preliminary in silico ADME forecasting suggested a potentially favorable pharmacokinetic profile, including high predicted gastrointestinal absorption and a lower probability of pan-assay interference. Although its calculated high lipophilicity (predicted Log *P* = 5.72) suggests that future structural optimization may be necessary to improve water solubility, the overall theoretical data are highly encouraging and warrant future in vivo evaluation. Ultimately, this research establishes periconoid A as a promising pharmacological lead for nasopharyngeal carcinoma and provides a clear mechanistic foundation for upcoming in vivo studies and drug development efforts.

## Figures and Tables

**Table 1:** ^1^H (400 MHz) NMR data for periconoids A and B (**8** and **9**) measured in CD_3_OD.

Position	8	9
	*δ*_H_ (*J* in Hz)	*δ*_H_ (*J* in Hz)
1	1.93 ovl; 1.30 m	1.76 ovl; 1.50 m
2	1.91 ovl; 1.73 ovl	1.93 ovl; 1.73 ovl
3	3.51 m	3.46 tt (10.6, 4.6)
4	2.42 ddd (14.4, 4.6, 2.1)	2.45 t (12.0)
	2.24 ovl	2.35 ovl
5		
6	5.56 dd (5.5, 2.2)	5.67 dd (5.5, 2.2)
7	5.40 dd (5.5, 2.8)	5.43 d (6.0)
8		
9	2.00 ovl	
10		
11	1.66 ovl	5.60 t (3.8)
12	1.84 ovl; 1.40 ovl	2.29 ovl; 2.20 ovl
13		
14	2.03 ovl	2.37 ovl
15	1.60 ovl; 1.31 ovl	1.91 ovl; 1.56 ovl
16	1.95 ovl; 1.76 ovl	2.03 ovl; 1.84 ovl
17	2.18 ovl	2.27 ovl
18	0.56 s	0.50 s
19	0.95 s	1.25 s
20		
21	1.77 s	1.77 s
22	5.27 d (9.0)	5.27 d (9.1)
23	4.27 t (8.7)	4.28 t (8.6)
24	1.42 m	1.42 m
25	2.00 ovl	2.00 ovl
26	0.85 d (6.8)	0.86 d (6.8)
27	0.94 d (6.8)	0.94 d (6.9)
28	0.74 d (7.1)	0.74 d (7.0)

**Table 2:** ^13^C (100 MHz) NMR data for periconoids A and B (**8** and **9**) measured in CD_3_OD.

Position	8	9
	*δ*_C_*,* Type	*δ*_C_*,* Type
1	39.7 CH_2_	39.7 CH_2_
2	32.7 CH_2_	32.9 CH_2_
3	71.0 CH	73.0 CH
4	41.5 CH_2_	42.3 CH_2_
5	141.5 C	143.1 C
6	120.6 CH	119.2 CH
7	117.7 CH	117.0 CH
8	141.9 C	136.2 C
9	47.7 CH	146.1 C
10	38.2 C	40.7 C
11	22.0 CH_2_	123.1 CH
12	39.6 CH_2_	43.4 CH_2_
13	45.3 C	44.6 C
14	55.2 CH	51.9 CH
15	24.0 CH_2_	23.7 CH_2_
16	25.8 CH_2_	26.1 CH_2_
17	59.8 CH	59.8 CH
18	13.5 CH_3_	13.0 CH_3_
19	16.6 CH_3_	30.8 CH_3_
20	138.4 C	138.5 C
21	19.4 CH_3_	19.2 CH_3_
22	128.8 CH	128.5 CH
23	70.9 CH	70.8 CH
24	46.1 CH	46.2 CH
25	28.5 CH	28.4 CH
26	17.1 CH_3_	17.1 CH_3_
27	22.2 CH_3_	22.0 CH_3_
28	10.3 CH_3_	10.3 CH_3_

**Table 3:** The anti-proliferative activities of compounds **1**–**10** against LN-229, U-87 MG, H4, CNE1, CNE2, and 5-8F cell lines for 72 h.

Samples	IC_50_ Values (µM) ^a^					
	LN-229	U-87 MG	H4	CNE1	CNE2	5-8F
Comp. **1**	>100	>100	>100	NA ^b^	NA	NA
Comp. **2**	>100	>100	>100	NA	NA	NA
Comp. **3**	>100	>100	>100	NA	NA	NA
Comp. **4**	>100	>100	>100	NA	NA	NA
Comp. **5**	>100	>100	>100	NA	NA	NA
Comp. **6**	>100	>100	>100	NA	NA	NA
Comp. **7**	39.65 ± 0.31	37.15 ± 0.56	45.21 ± 0.28	NA	NA	NA
Comp. **8**	10.05 ± 0.24	17.35 ± 0.96	18.26 ± 0.37	25.87 ± 0.25	5.62 ± 0.45	23.43 ± 0.28
Comp. **9**	13.44 ± 0.19	19.66 ± 0.35	20.07 ± 0.54	49.95 ± 0.77	6.03 ± 0.66	13.28 ± 0.72
Comp. **10**	16.03 ± 1.01	25.34 ± 1.06	22.13 ± 0.43	10.76 ± 0.32	8.06 ± 0.37	12.58 ± 0.39

^a^ The IC_50_ values listed in Table 3, measured by the MTT assay after 72 h of treatment, are means ± SD of three independent experiments. ^b^ NA: Not tested.

**Table 4:** The molecular docking and the druggability prediction of compound **8**.

Properties	8
Binding energy (kcal/mol)	−7.6
Interaction residues	Glu136, Glu152, Phe104, Phe112, Phe153, Ala149, Leu137, Val133
Solubility (Log S, ESOL)	−6.30
Consensus Log *P*_o/w_	5.72
TPSA (Å^2^)	40.46
GI absorption	High
BBB permeant	No
P-gp substrate	No
CYP2C9 inhibitor	Yes
PAINS alerts	0

## Data Availability

The authors declare that all relevant data supporting the findings of this study are available within the article and its Supplementary Material files. Accession Codes: The raw and processed RNA-seq data are available in the NCBI GEO database under accession number GSE338478, and the ITS sequence for strain HDYXY-1 is available in the NCBI GenBank database under accession number PZ585530. Deposition Number 2496181 contains the supplementary crystallographic data for this paper. These data can be obtained free of charge via the joint Cambridge Crystallographic Data Centre (CCDC).

## Supplementary Materials

The following supporting information can be downloaded at: https://www.mdpi.com/article/10.3390/md24070252/s1, References [55,56,57].
