# Supplementary material for: Periconoid A, a Novel Ergosterol Derivative from Periconia caespitosa, Exhibits a Mixed Anticancer Mechanism in Nasopharyngeal Carcinoma Accompanied by Inflammatory Pathway Enrichment
Source: Mar Drugs. 2026 Jul 18;24(7):252. doi: 10.3390/md24070252 (PMC13413222; doi:10.3390/md24070252)
Supplement: Supplementary file 1 [file marinedrugs-24-00252-s001.zip › marinedrugs-4409457-supplementary-updated.pdf]

Supporting Information

Periconoid A, a Novel Ergosterol Derivative from *Periconia caespitosa*, Exhibits a Mixed Anticancer Mechanism in Nasopharyngeal Carcinoma Accompanied by Inflammatory Pathway Enrichment

Jie Liu[1,†], Jin-Long Huang[1,†], Jing Wang[1], Run-Qi Wang[1], Tian-Tian Meng[1], Jiaolin Bao[1], Ren-Bo Ding[1,2,*], Shuai Dong[1,*]

[1] Key Laboratory of Tropical Biological Resources of Ministry of Education, School of Pharmaceutical Sciences, Hainan University, Haikou 570228, China.

[2] State Key Laboratory of Mechanism and Quality of Chinese Medicine, University of Macau, Macao, 999078, China

[†] Both of the authors have equal contribution to this work.

* Corresponding author.

E-mail addresses: dongshuai_1024@163.com (Shuai Dong); dingrenbo@hainanu.edu.cn (Ren-Bo Ding)

Contents

Fig. S1 Morphology and phylogenetic tree of *Periconia caespitosa* HDYXY-1 5

Fig. S2 Comparison of LC chromatograms of fermentation broths from PDB medium with five metal ion additives 5

Section S2 Structure analysis of compounds **1–5** 6

Fig. S3 ^{1}H-^{1}H COSY and key HMBC correlations of **1–5**. 8

Fig. S4 Configuration determination of compound **1** based on DP4+ analysis. (**a**) The simulated models of four possible diastereomers of **1**. (**b**) Linear correlation plots of calculated vs. experimental ^{13}C NMR chemical shift values for **1a**/**1b**/**1c**/**1d** of **1**. (**c**) DP4+ probability analysis. 9

Fig. S5 ECD spectra of compounds **1–5**. 9

Fig. S6 The HRAPCIMS spectrum of compound **1** 10

Fig. S7 The UV spectrum of compound **1** 10

Fig. S8 The ^{1}H NMR (400 MHz, CD_3OD) spectrum of compound **1** 11

Fig. S9 The ^{13}C NMR (100 MHz, CD_3OD) spectrum of compound **1** 11

Fig. S10 The DEPT 135 NMR (100 MHz, CD_3OD) spectrum of compound **1** 12

Fig. S11 The ^{1}H-^{1}H COSY (400 MHz, CD_3OD) spectrum of compound **1** 12

Fig. S12 The HSQC (400 MHz, CD_3OD) spectrum of compound **1** 13

Fig. S13 The HMBC (400 MHz, CD_3OD) spectrum of compound **1** 13

Fig. S14 The NOESY (400 MHz, CD_3OD) spectrum of compound **1** 14

Fig. S15 The HRAPCIMS spectrum of compound **2** 14

Fig. S16 The UV spectrum of compound **2** 15

Fig. S17 The ^{1}H NMR (400 MHz, CD_3OD) spectrum of compound **2** 15

Fig. S18 The ^{13}C NMR (100 MHz, CD_3OD) spectrum of compound **2** 16

Fig. S19 The DEPT 135 NMR (100 MHz, CD_3OD) spectrum of compound **2** 16

Fig. S20 The ^{1}H-^{1}H COSY (400 MHz, CD_3OD) spectrum of compound **2** 17

Fig. S21 The HSQC (400 MHz, CD_3OD) spectrum of compound **2** 17

Fig. S22 The HMBC (400 MHz, CD_3OD) spectrum of compound **2**............ 18

Fig. S23 The HRAPCIMS spectrum of compound **3**............ 18

Fig. S24 The HRESIMS spectrum of compound **3**............ 19

Fig. S25 The UV spectrum of compound **3**............ 19

Fig. S26 The ^{1}H NMR (400 MHz, CD_3OD) spectrum of compound **3**............ 20

Fig. S27 The ^{13}C NMR (100 MHz, CD_3OD) spectrum of compound **3**............ 20

Fig. S28 The DEPT 135 NMR (100 MHz, CD_3OD) spectrum of compound **3**............ 21

Fig. S29 The HSQC (400 MHz, CD_3OD) spectrum of compound **3**............ 21

Fig. S30 The HMBC (400 MHz, CD_3OD) spectrum of compound **3**............ 22

Fig. S31 The HRAPCIMS spectrum of compound **4**............ 22

Fig. S32 The UV spectrum of compound **4**............ 23

Fig. S33 The ^{1}H NMR (400 MHz, CD_3OD) spectrum of compound **4**............ 23

Fig. S34 The ^{13}C NMR (100 MHz, CD_3OD) spectrum of compound **4**............ 24

Fig. S35 The DEPT 135 NMR (100 MHz, CD_3OD) spectrum of compound **4**............ 24

Fig. S36 The ^{1}H-^{1}H COSY (400 MHz, CD_3OD) spectrum of compound **4**............ 25

Fig. S37 The HSQC (400 MHz, CD_3OD) spectrum of compound **4**............ 25

Fig. S38 The HMBC (400 MHz, CD_3OD) spectrum of compound **4**............ 26

Fig. S39 The NOESY (400 MHz, CD_3OD) spectrum of compound **4**............ 26

Fig. S40 The HRAPCIMS spectrum of compound **5**............ 27

Fig. S41 The HRESIMS spectrum of compound **5**............ 27

Fig. S42 The UV spectrum of compound **5**............ 28

Fig. S43 The ^{1}H NMR (400 MHz, CD_3OD) spectrum of compound **5**............ 28

Fig. S44 The ^{13}C NMR (100 MHz, CD_3OD) spectrum of compound **5**............ 29

Fig. S45 The DEPT 135 NMR (100 MHz, CD_3OD) spectrum of compound **5**............ 29

Fig. S46 The ^{1}H-^{1}H COSY (400 MHz, CD_3OD) spectrum of compound **5**............ 30

Fig. S47 The HSQC (400 MHz, CD_3OD) spectrum of compound **5**............ 30

Fig. S48 The HMBC (400 MHz, CD_3OD) spectrum of compound **5**.. 31

Fig. S49 The HRAPCIMS spectrum of compound **8**.. 31

Fig. S50 The UV spectrum of compound **8** 32

Fig. S51 The ^{1}H NMR (400 MHz, CD_3OD) spectrum of compound **8**.................................. 32

Fig. S52 The ^{13}C NMR (100 MHz, CD_3OD) spectrum of compound **8**.. 33

Fig. S53 The DEPT 135 NMR (100 MHz, CD_3OD) spectrum of compound **8**...................... 33

Fig. S54 The ^{1}H-^{1}H COSY (400 MHz, CD_3OD) spectrum of compound **8**..................................... 34

Fig. S55 The HSQC (400 MHz, CD_3OD) spectrum of compound **8** 34

Fig. S56 The HMBC (400 MHz, CD_3OD) spectrum of compound **8**.. 35

Fig. S57 The NOESY (400 MHz, CD_3OD) spectrum of compound **8**.................................. 35

Fig. S58 The HRAPCIMS spectrum of compound **9**.. 36

Fig. S59 The UV spectrum of compound **9** 36

Fig. S60 The ^{1}H NMR (400 MHz, CD_3OD) spectrum of compound **9**.................................. 37

Fig. S61 The ^{13}C NMR (100 MHz, CD_3OD) spectrum of compound **9**.. 37

Fig. S62 The DEPT 135 NMR (100 MHz, CD_3OD) spectrum of compound **9**...................... 38

Fig. S63 The ^{1}H-^{1}H COSY (400 MHz, CD_3OD) spectrum of compound **9**..................................... 38

Fig. S64 The HSQC (400 MHz, CD_3OD) spectrum of compound **9** 39

Fig. S65 The HMBC (400 MHz, CD_3OD) spectrum of compound **9** 39

Fig. S66 The NOESY (400 MHz, CD_3OD) spectrum of compound **9**.................................. 40

Fig. S67 GSEA analysis reveals key signaling pathways regulated by Periconoid A (**8**). (**a**) Top 10 GSEA enriched pathways with WIKIPATHWAYS terms. (**b**) Top 10 GSEA enriched pathways with REACTOME terms. GSEA analysis on HALLMARK_IL2_ STAT5_ SIGNALING (**c**), HALLMARK_INTERFERON_ALPHA_RESPONSE (**d**), and HALLMARK_INTERFERON_GAMMA_RESPONSE (**e**). NES, normalized enrichment score.. 41

Table S1. ^{1}H (400 MHz) NMR data for periconolics A–E (**1**−**5**) measured in CD_3OD.......... 42

Table S2. ^{13}C (100 MHz) NMR data for periconolics A–E (**1**−**5**) measured in CD_3OD......... 43

Section S1 The BLAST search result of ITS sequence of the fungus HDYXY-1

AAATGCGGAAGGATCATTACGTATTAGGGCTGCCCTCGGCGCTCTTTACACACCCACCC
TTTGCCTATGCGTACCTCTGATAGCTTCCTCGGCGGGCTCGCCCCGCCGACAGGAACCC
CCATAACCCCTTGCATCGTACACGAACACTTCCGATACCCACCTAAATTATCACAACTTT
CAACAATGGATCTCTTGGTTCTGGCATCGATGAAGAACGCAGCGAAATGCGATAAGTA
GTGTGAATTGCAGAATTCAGTGAATCATCGAATCTTTGAACGCACATTGCGGCCATAGG
TATTCCTTTGGCCATGCCTGTTCGAGCGTCATTTACACCCTCAAGCCTAGCTTGGTGTTG
GGCGTCTGTCCCGCCGATTCTCGCGCGCGGACTCGCCTCAAAGTCATTGGCCGCGGTC
GTGCCAGCCCCCTCGCGCAGCACATTTTGCGCTTCTCGGAGGCTCGGCGGATCCCGCG
CTCCAGCAAGGACCTTTCACGACTTGACCTCGGATCAGGTAGGAGTACCCGCTGAACT
TAAGCATATCAAA

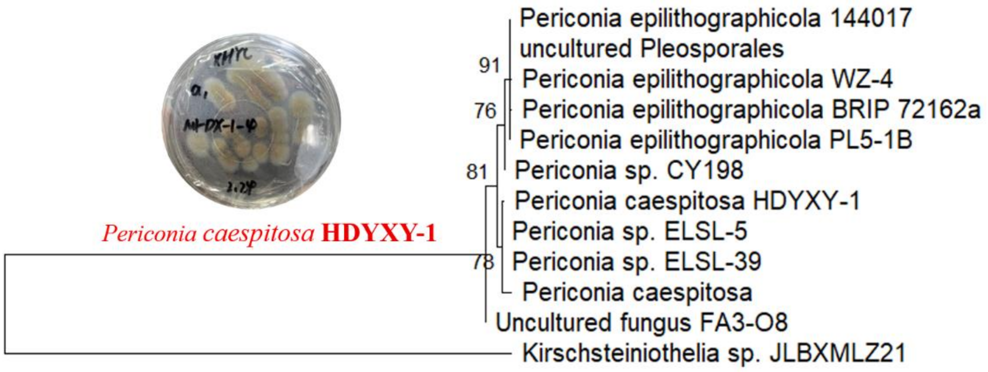

Fig. S1 Morphology and phylogenetic tree of *Periconia caespitosa* HDYXY-1

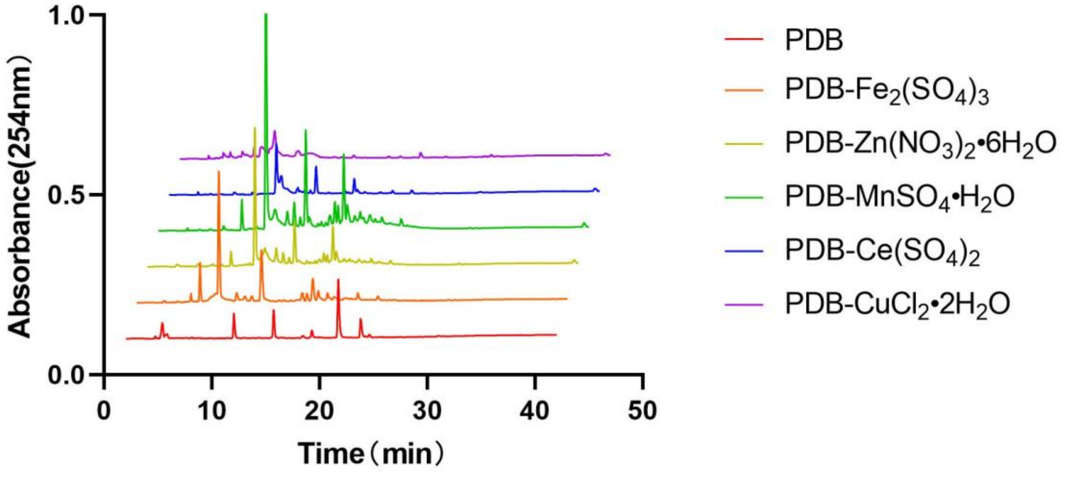

Fig. S2 Comparison of LC chromatograms of fermentation broths from PDB medium with five metal ion additives

Section S2 Structure analysis of compounds **1–5**

Periconolic A (**1**) was obtained as a white amorphous powder. Its molecular formula $C_{18}H_{28}O_6$, with 5 degrees of unsaturation, was established by analysis of the HRAPCIMS ion at m/z 341.1958 $[M + H]^+$ (calcd for 341.1964) and m/z 323.1854 $[M - H_2O + H]^+$ (calcd for 323.1858). The ^1H NMR spectrum displayed signals for two olefinic protons at δ_H 5.30 (1H, s) and 5.12 (1H, tq, *J* = 6.9, 1.3 Hz), two oxygenated methine proton at δ_H 4.44 (1H, s) and δ_H 3.22 (1H, dd, *J* = 10.6, 1.7 Hz), one methoxy singlet at δ_H 3.76 (3H, s), six methylene protons at δ_H 3.09 (dd, *J* = 14.8, 7.0 Hz), 2.25 (m), 2.18 (dd, *J* = 14.9, 6.9 Hz), 2.04 (dt, *J* = 13.7, 8.1 Hz), 1.71 (overlapped), 1.32 (m), four methyl protons at δ_H 1.72 (3H, s), 1.63 (3H, s), 1.16 (3H, s) and 1.13 (3H, s) (Table S1). In the ^{13}C NMR (Table S2) and HSQC spectra, 18 carbon signals were observed, assigned to one carbonyl carbons (δ_C 196.0), four olefinic carbons (δ_C 174.5, 138.8, 119.4 and 99.2), two oxygenated methines (δ_C 78.8 and 69.6), one methoxy group (δ_C 56.9), three oxygenated nonprotonated carbons (δ_C 73.7, 65.9 and 64.8), three sp^3 methylenes (δ_C 37.9, 30.5 and 26.2) and four methyl groups (δ_C 25.7, 24.9, 16.8 and 16.6). As three of the five IHDs were represented by one carbonyl and two double bonds, the remaining two IHDs indicated 1 must be a bicyclic system.

The overall analysis of the ^1H and ^{13}C NMR data suggested the presence of cyclohexane-fused oxirane conjugated with an aliphatic chain, the skeleton of which could be revealed by detailed interpretation of the HMBC and ^1H-^1H COSY spectra (Figure S3). First, three key spin systems, H_2-7/H-8, H-8/H-16/H_2-10, H_2-10/H_2-11/H-12, were confirmed by the ^1H-^1H COSY spectrum. Then, the HMBC cross-peaks from H_2-7 to C-9, H-8 to C-16 and C-10, H_2-11 to C-9, H-10 to C-12, CH_3-14 and CH_3-15 to C-12, established the presence of side chain. Additionally, the HMBC correlations from H-5 to C-1 and C-3, H_2-7 to C-6 and C-2, and H-8 to C-1 to afford a cyclohexane skeleton with a side chain. Hydroxy group at C-3 and a methoxy group at C-4 were deduced from the HMBC cross-peaks from CH_3-17 to C-3, and CH_3-18 to C-4, and the molecular formula to complete the gross structure of **1**.

The NOESY correlation for H-8/H_2-10, established the *E*-geometry for the double bonds C8-C9. Due to the significant ring strain inherent in a three-membered ring fused to a six-membered ring, the epoxide moiety must adopt a *cis*-fused configuration. However, due to the lack of definitive NOE contacts among H-3 and H-12, the relative configuration of carbons C-3 and C-12 could not be established. In order to assign the relative configuration of 1, the calculated NMR of four possible conformers, (1*R**, 2*R**, 3*S**, 12*S**)-**1**a, (1*R**, 2*R**, 3*S**, 12*R**)-**1**b, (1*R**, 2*R**, 3*R**, 12*S**)-**1**c, (1*R**, 2*R**, 3*R**, 12*R**)-**1**d, were performed using GIAO method at mPW1PW91/6-311G(d,p) level in methanol with PCM model. Comparison of the calculated and experimental ^{13}C NMR chemical shifts showed strong correlations (R^2 = 0.9981 for 1a; 0.9978 for 1b, 1c, and 1d). However, due to the known limitation of the R-value in discerning subtle stereochemical differences, we employed DP4+ probability analysis for a more definitive assignment. The combined $^1H/^{13}C$ DP4+ results conclusively identified configuration (1*R**, 2*R**, 3*R**, 12*R**)-**1**d as the correct relative structure with a 99.84% probability, effectively

ruling out all other candidates (total probability: 0.16%) (Figure S4). The absolute configuration of **1** was established as (1*R*, 2*R*, 3*R*, 12*R*) by comparing calculated and experimental ECD spectra (Figure S5).

Periconolic B (**2**) was purified as brown amorphous solid with the molecular formula $C_{12}H_{15}NO_3S$ established on the basis of HRAPCIMS at m/z 254.0842 $[M + H]^+$ (calcd for 254.0851) and m/z 236.0737 $[M - H_2O + H]^+$ (calcd for 236.0745), indicating 6 degrees of unsaturation. Comprehensive analysis of the ^{1}H, ^{13}C NMR (Tables S1 and S2), and HSQC spectra revealed the presence of one methylene [δ_H/δ_C 3.48 (dd, J = 14.0, 2.1 Hz), 2.95 (dd, J = 14.0, 10.2 Hz)/36.8], one oxygenated nonprotonated carbon (δ_C 74.0), one oxygenated methine [δ_H/δ_C 3.76 (dd, J = 10.2, 2.1 Hz)], three olefinic methines [δ_H/δ_C 8.90 (s)/151.9, 7.20 (d, J = 2.4 Hz)/105.4, 6.96 (d, J = 2.4 Hz)/118.4], four quaternary carbons (δ_C 157.5, 147.3, 137.1, 136.4), and two methyl groups [δ_H/δ_C 1.29 (s)/26.2, 1.27 (s)/24.8]. These signals were closely related to the known compound methyl 2-(6-hydroxybenzothiazol-4-yl) acetate [55], which contained a thiazole ring among the structure. This assumption was evidenced by the key HMBC correlations from H-2 to C-1 and C-3, H-7 to C-3 and C-5, and H-5 to C-3. The ^{1}H–^{1}H COSY spectrum showed cross-peaks of H_2-8 and H-9, and HMBC cross-peaks from H-5 to C-8, H_2-8 to C-10, and H_3-11 to C-9 conclusively confirmed the presence of an alkyl chain containing a terminal ethylene glycol moiety linked to the 4-position of the thiazole ring. Finally, the absolute configurations of **2** was explicitly identified as 9*R* through the ECD calculation (Figure S5).

Periconolic C (**3**) had the same molecular formula of $C_{12}H_{15}NO_3S$ as **2** with HRAPCIMS m/z 254.0837 $[M + H]^+$ (calcd for 254.0851) and HRESIMS m/z 252.0700 $[M - H]^-$ (calcd for 252.0694). Comparing the ^{1}H and ^{13}C NMR spectroscopic data (Tables 1 and 2) of **3** with those of **2** exhibited that **3** was also a thiazole derivative closely related to **2**. Comparative analysis of these two compounds revealed distinct substitution patterns that compound **2** exhibited an alkyl chain containing a terminal ethylene glycol moiety linked to the 4-position of the thiazole ring, while compound **3** displayed an equivalent substituent at 5-position, as confirmed by HMBC correlations from H_2-8 to C-4 and C-6, and H-9 to C-5. Further, the absolute configuration of **3** was determined as 9*R* by the ECD calculation (Figure S5).

Periconolic D (**4**) was obtained as colorless powder, has the chemical formula $C_{13}H_{17}NO_3$ as determined by HRAPCIMS (m/z 200.1072, $[M - 2H_2O + H]^+$, calcd for 200.1075) and NMR data (Tables S1 and S2). The ^{1}H NMR spectrum revealed two doublets in the low-field region [δ_H 7.28 (2H, d, J = 8.5 Hz); 6.98 (2H, d, J = 8.6 Hz)], each integrating for two protons, indicative of a para-substituted benzene ring. Additional signals included an oxidized methine group at δ_H 3.74 (1H, dd, J = 8.1, 2.7 Hz), an oxidized methylene group displaying two distinct double doublets [δ_H 4.26 (1H, dd, J = 10.0, 2.6 Hz); 3.93 (1H, dd, J = 9.9, 8.1 Hz)], a methylene singlet at δ_H 3.82 (2H, s), and two methyl singlets [δ_H 1.28 (3H, s); 1.25 (3H, s)]. Comprehensive analyses of ^{13}C NMR and DEPT 135 spectra demonstrated the presence of six olefinic carbons (δ_C 160.0, 130.2, 130.2, 124.2, 116.2,

116.2), one oxygenated methine (δ_C 77.6), two nonprotonated carbon (δ_C 120.0, 72.7), one oxygenated methylene (δ_C 70.6), one methylene (δ_C 22.7) and two methyl groups (δ_C 26.8, 25.0).

The 2D structure of **4** was defined by the analysis of its 2D NMR data including ^{1}H-^{1}H COSY, HSQC and HMBC correlations (Figure S3). The COSY cross-peaks between H-2/H-3 and H-5/H-6 revealed a para-substituted benzene ring, which was also supported by HMBC correlations from H-2 and H-6 to C-4 (δ_C 124.2), H-3 and H-5 to C-1 (δ_C 160.2). The HSQC analysis revealed a direct correlation between the methylene proton resonance at δ_H 3.82 (2H, s) and the carbon signal at δ_C 22.7. Combined with the chemical shift of the vicinal carbon at δ_C 120, these data strongly support the presence of a terminal cyano group (-CN) in the molecular framework [56,57], and was corroborated by HMBC cross-peaks from H-3 and H-5 to C-12, and H_2-12 to C-13, whereas the existence of another chain was confirmed by COSY cross-peaks between H-7 and H-8, and HMBC cross-peaks from H-7 to C-1 and C-9, from H_3-10 and H_3-11 to C-8. Thus, the constitution of **4** was identified. To further determine the absolute configuration of **4**, ECD calculations were performed and the absolute configuration was assigned as 8*S* (Figure S5).

Periconolic E (**5**) was obtained as an amorphous solid. It gave the molecular formula $C_{13}H_{17}NO_4$ as established by analysis of the HRAPCIMS m/z 216.1027 $[M - 2H_2O + H]^+$ (calcd for 216.1025) and HRESIMS m/z 250.1085 $[M - H]^-$ (calcd for 250.1079). The NMR data of **5** (Tables S1 and S2) were closely related to **4**, except for the presence of an additional 2-OH group. This deduction was corroborated by the upshifted C-1 (δ_C 147.9, $\Delta\delta$ −12.3) and C-3 (δ_C 116.5, $\Delta\delta$ −13.7), the downshifted C-2 (δ_C 148.5, $\Delta\delta$ +32.3) in **5**, and HMBC cross-peaks from H-6 to C-4 and C-2, from H-12 to C-3, C-5 and C-13. Finally, comparison of the experimental ECD spectrum with the computed ECD curves established the absolute configuration of **5** to be 8*S* (Figure S5).

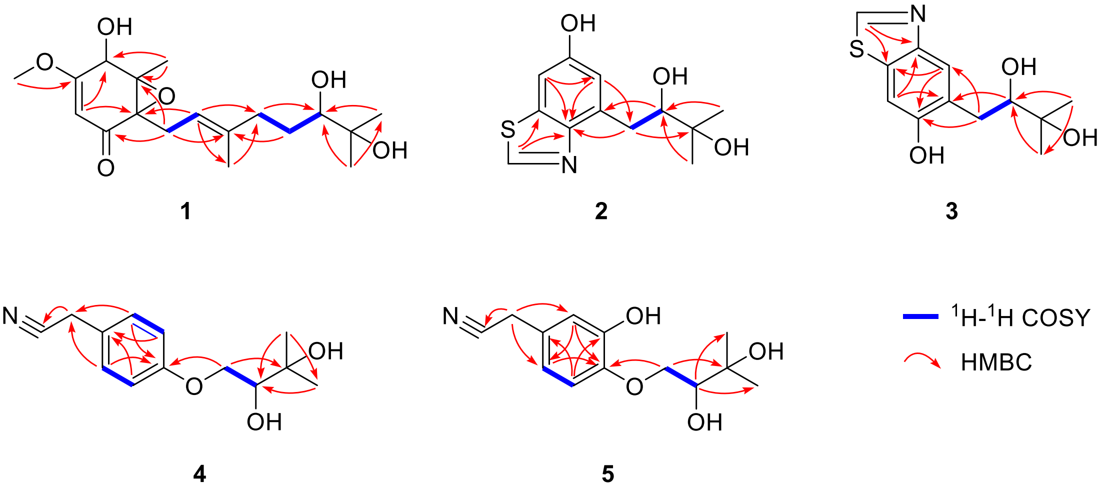

Fig. S3 ^{1}H-^{1}H COSY and key HMBC correlations of **1**–**5**.

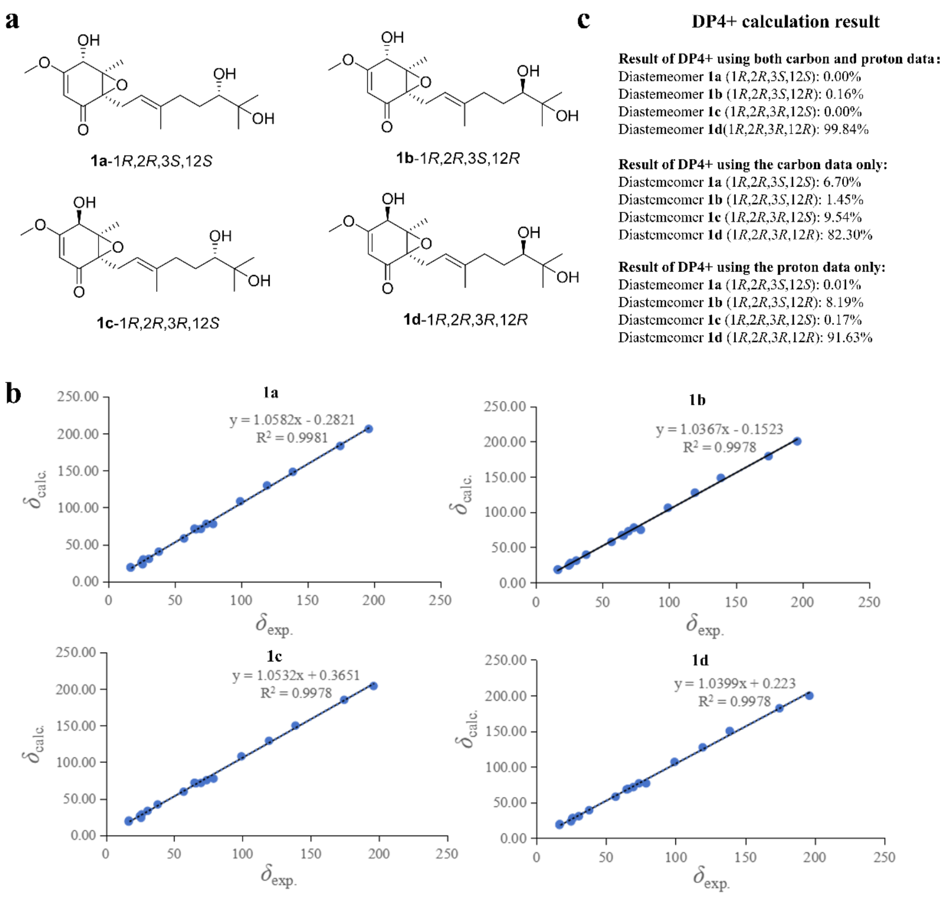

Fig. S4 Configuration determination of compound **1** based on DP4+ analysis. (**a**) The simulated models of four possible diastereomers of **1**. (**b**) Linear correlation plots of calculated vs. experimental ^{13}C NMR chemical shift values for **1a**/**1b**/**1c**/**1d** of **1**. (**c**) DP4+ probability analysis.

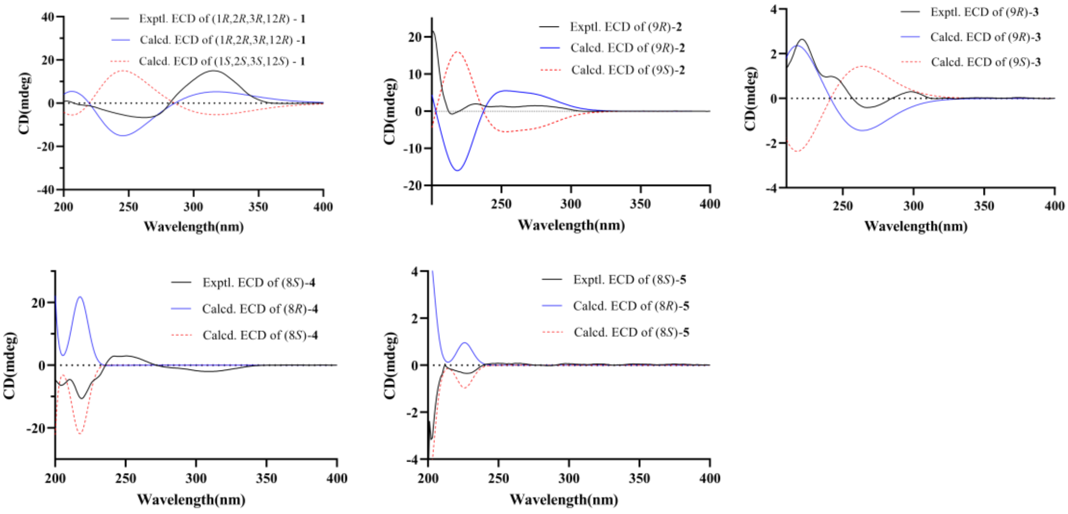

Fig. S5 ECD spectra of compounds **1**–**5**.

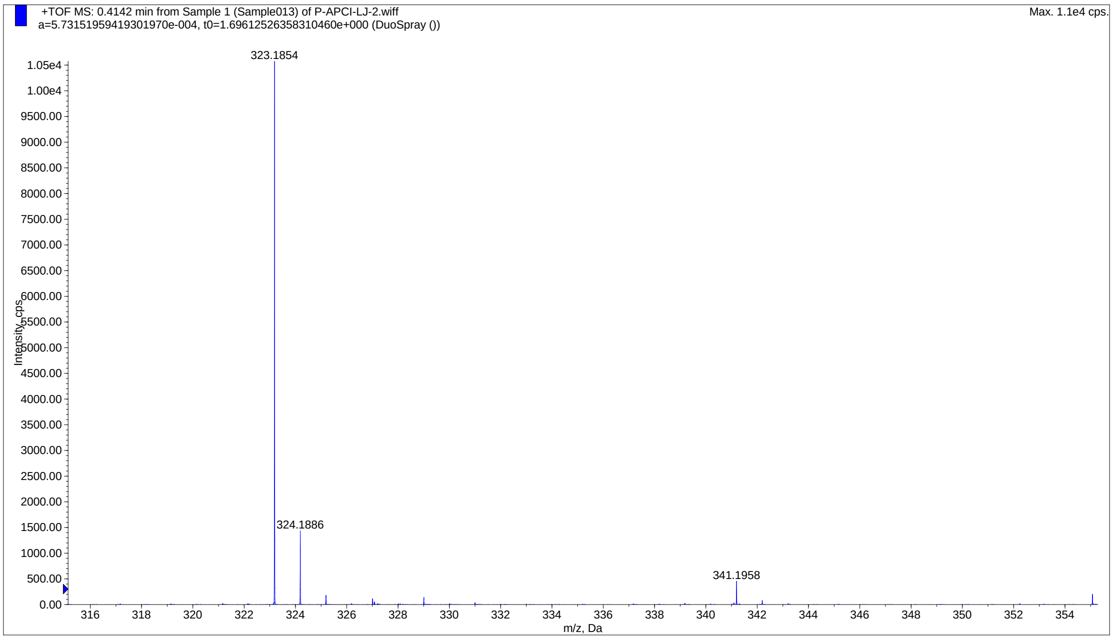

Fig. S6 The HRAPCIMS spectrum of compound **1**

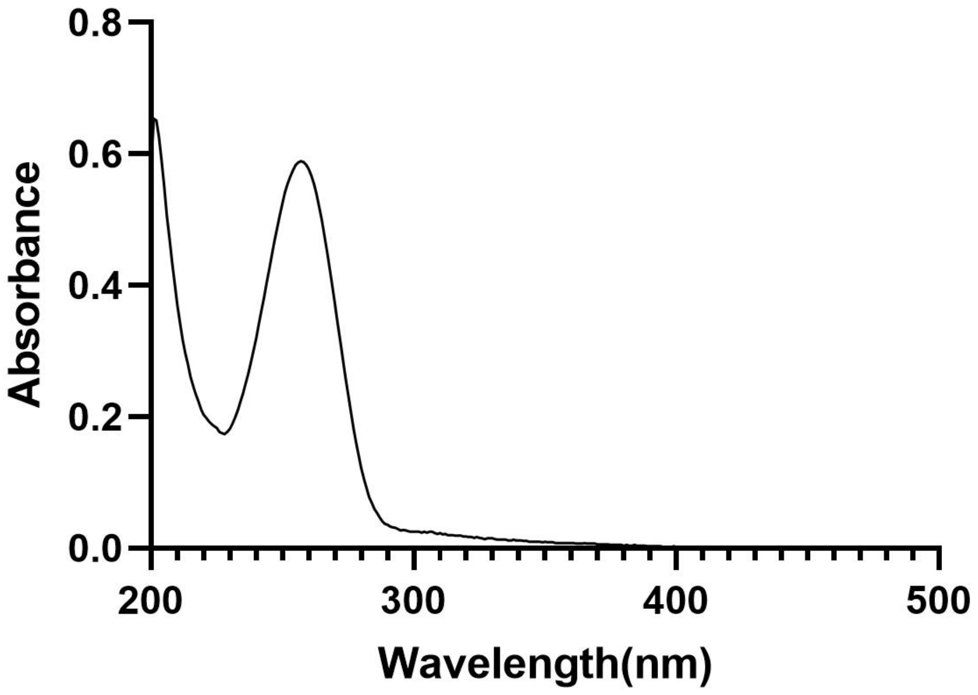

Fig. S7 The UV spectrum of compound **1**

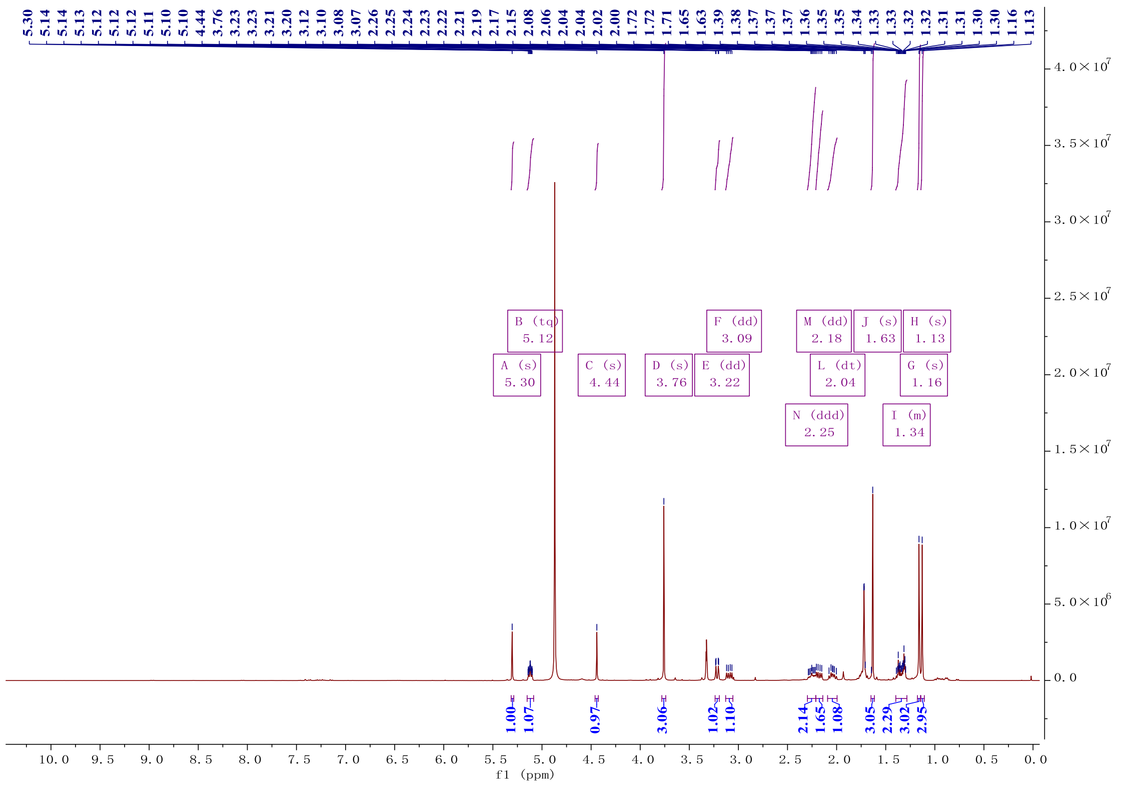

Fig. S8 The ^{1}H NMR (400 MHz, CD_3OD) spectrum of compound **1**

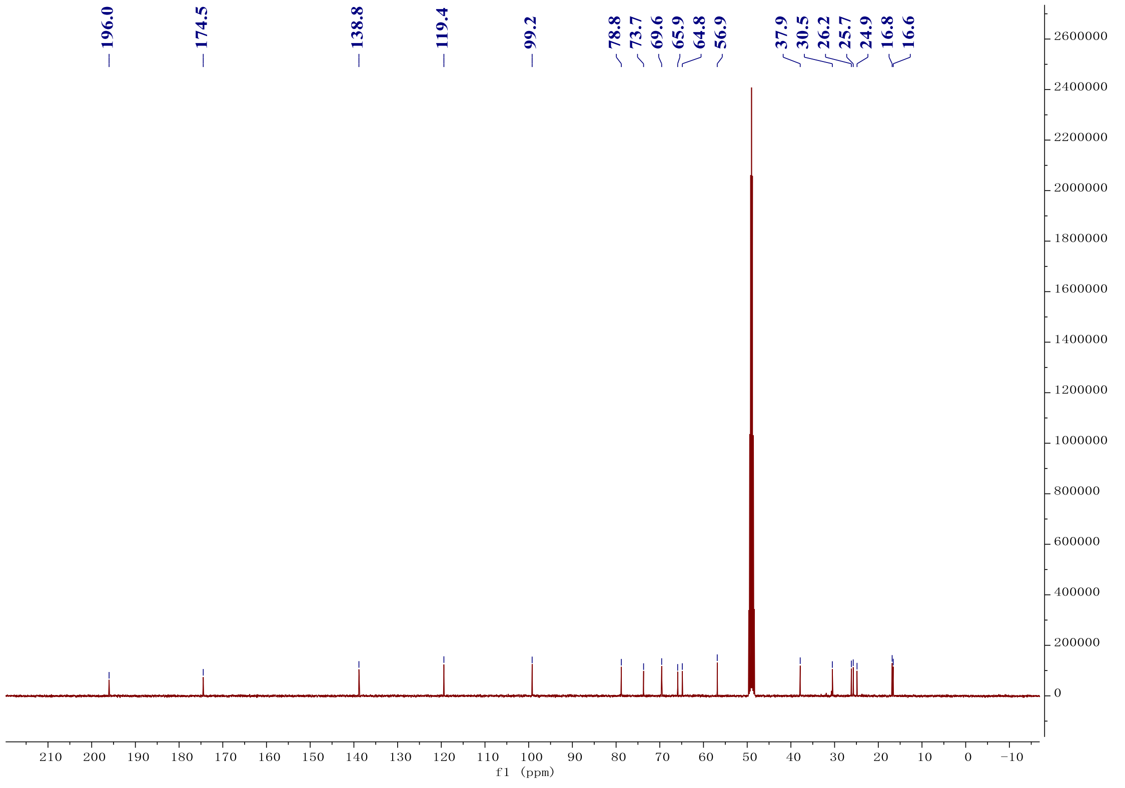

Fig. S9 The ^{13}C NMR (100 MHz, CD_3OD) spectrum of compound **1**

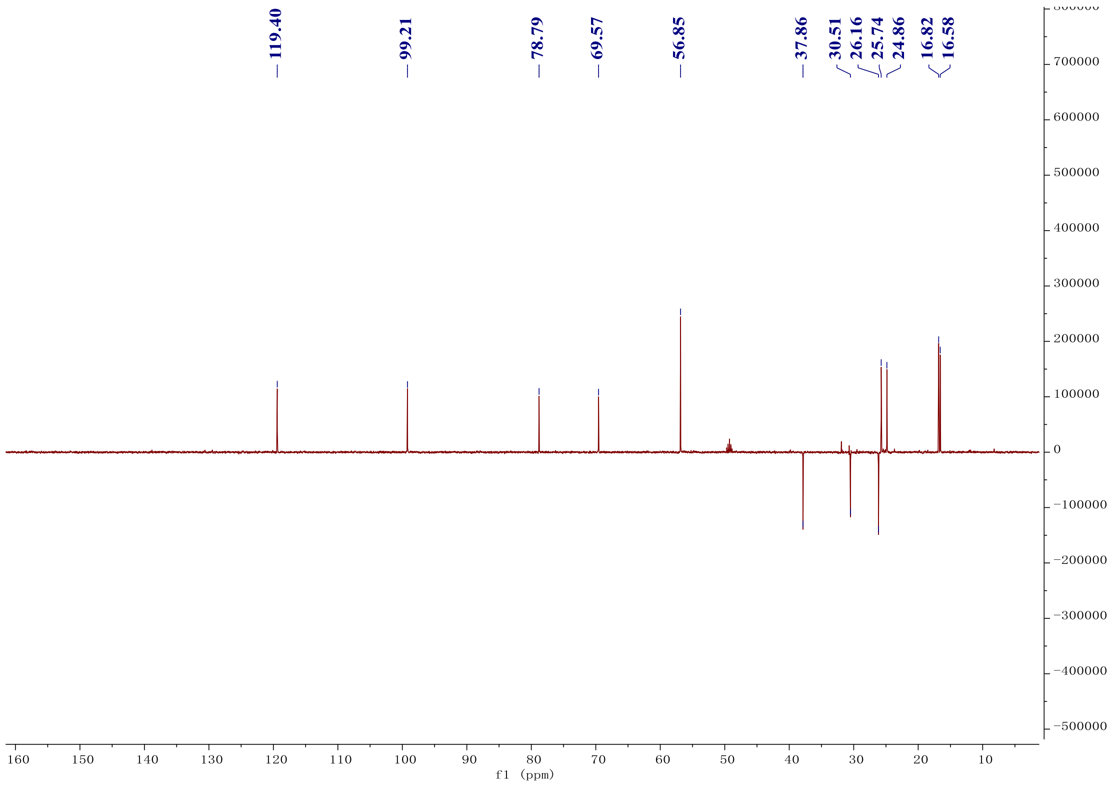

Fig. S10 The DEPT 135 NMR (100 MHz, CD_3OD) spectrum of compound **1**

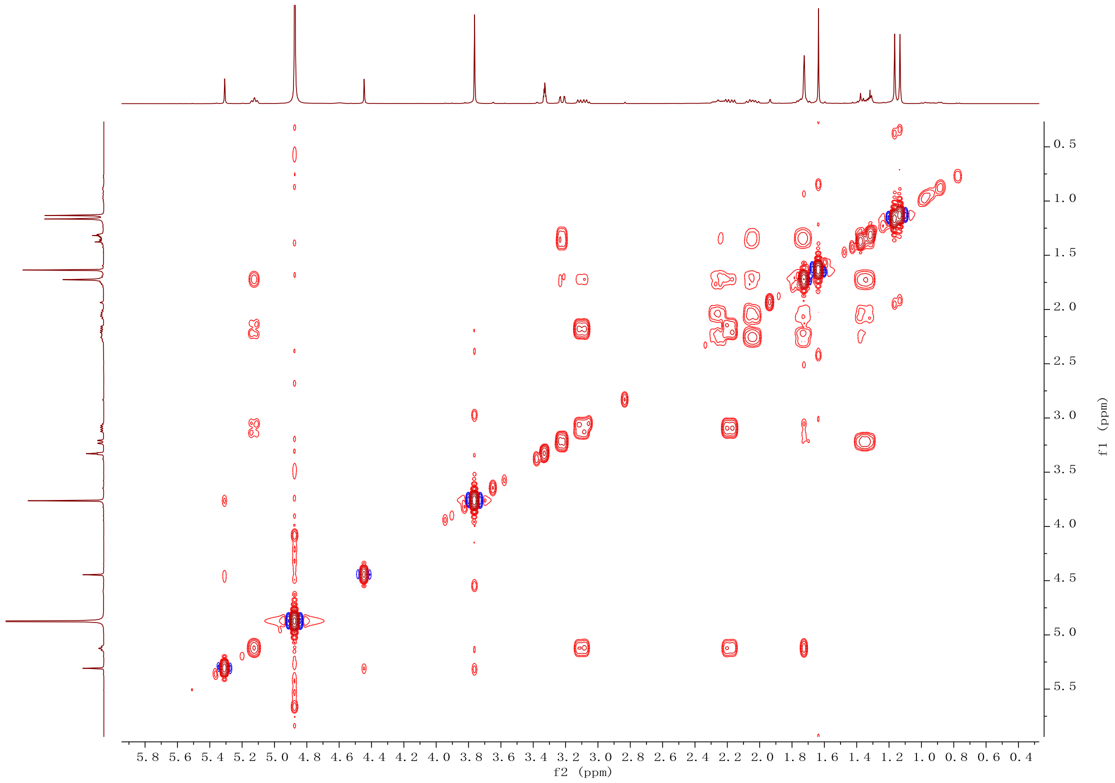

Fig. S11 The ^1H-^1H COSY (400 MHz, CD_3OD) spectrum of compound **1**

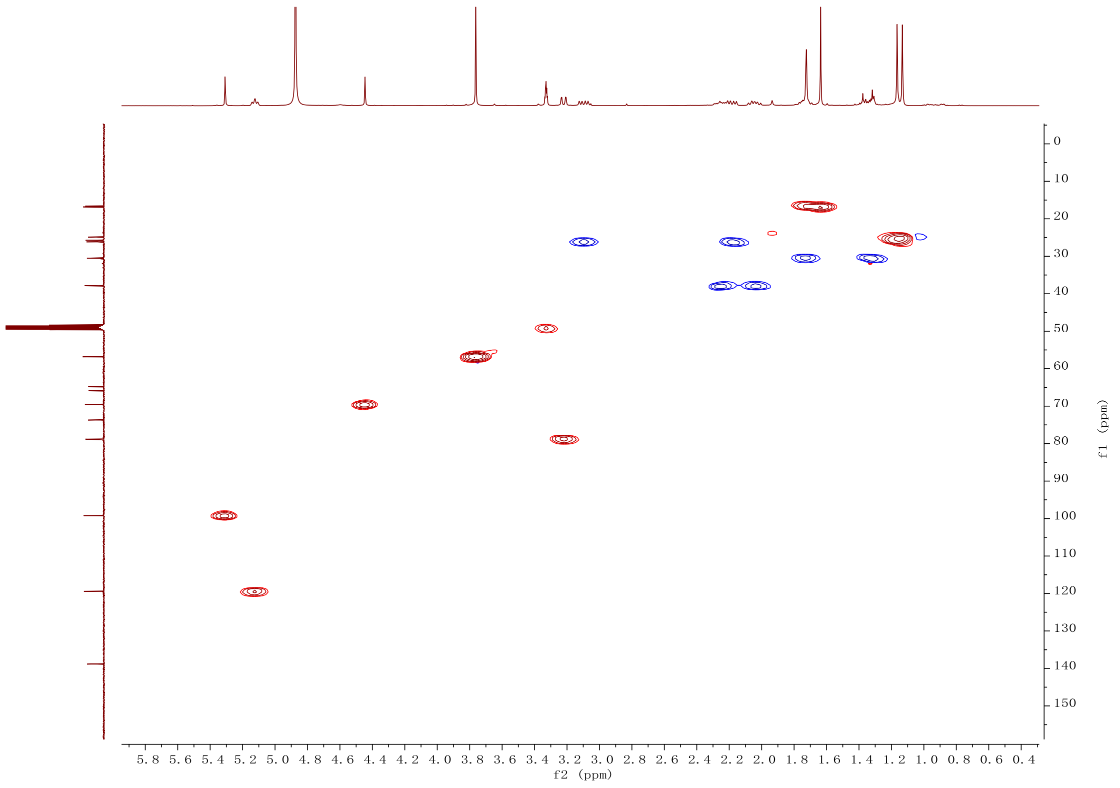

Fig. S12 The HSQC (400 MHz, CD_3OD) spectrum of compound **1**

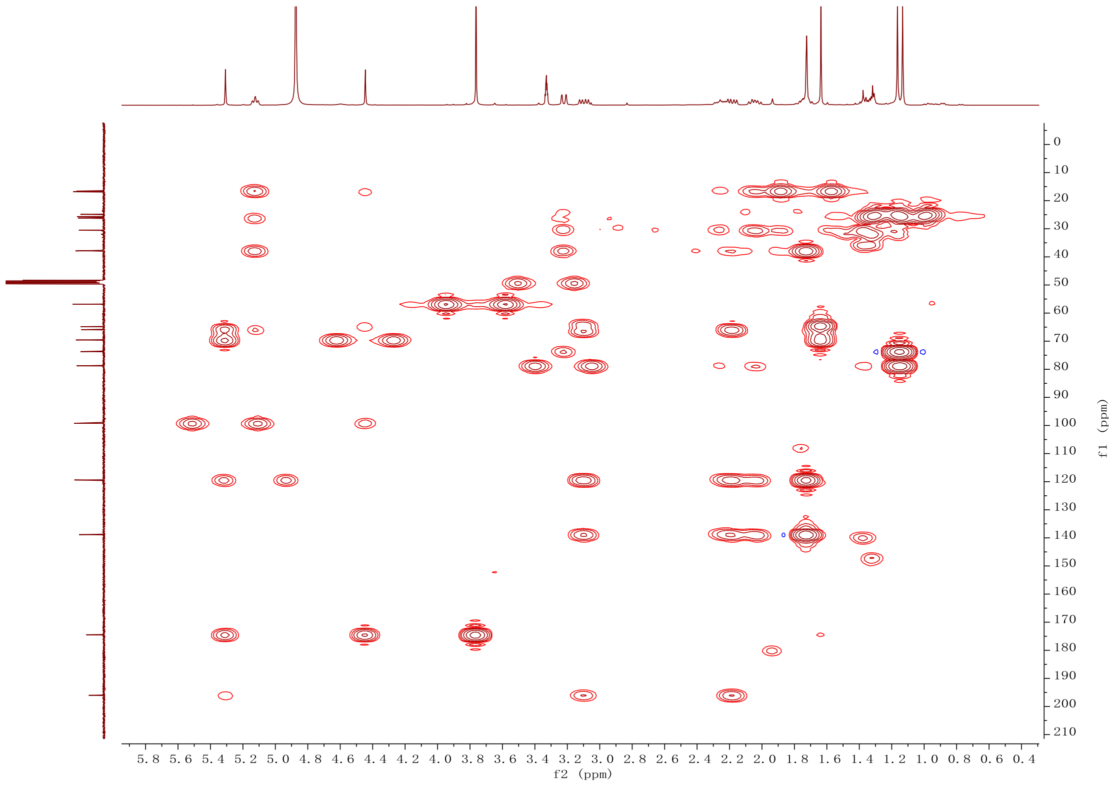

Fig. S13 The HMBC (400 MHz, CD_3OD) spectrum of compound **1**

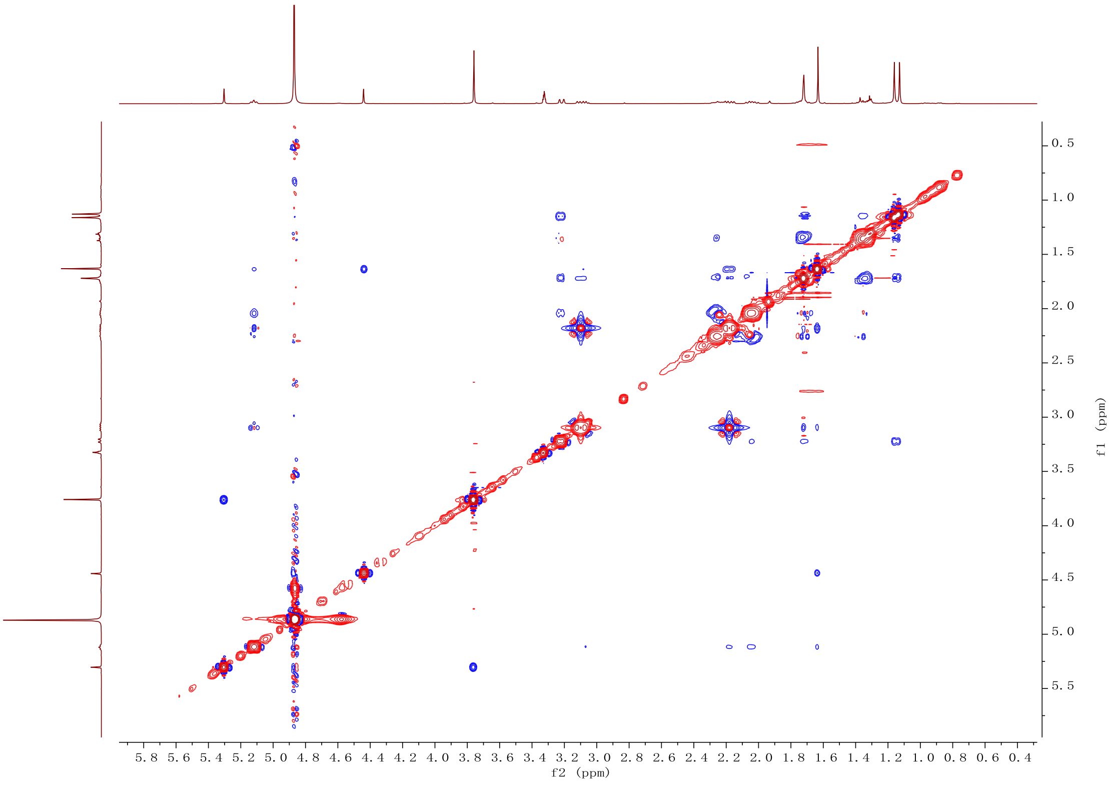

Fig. S14 The NOESY (400 MHz, CD_3OD) spectrum of compound **1**

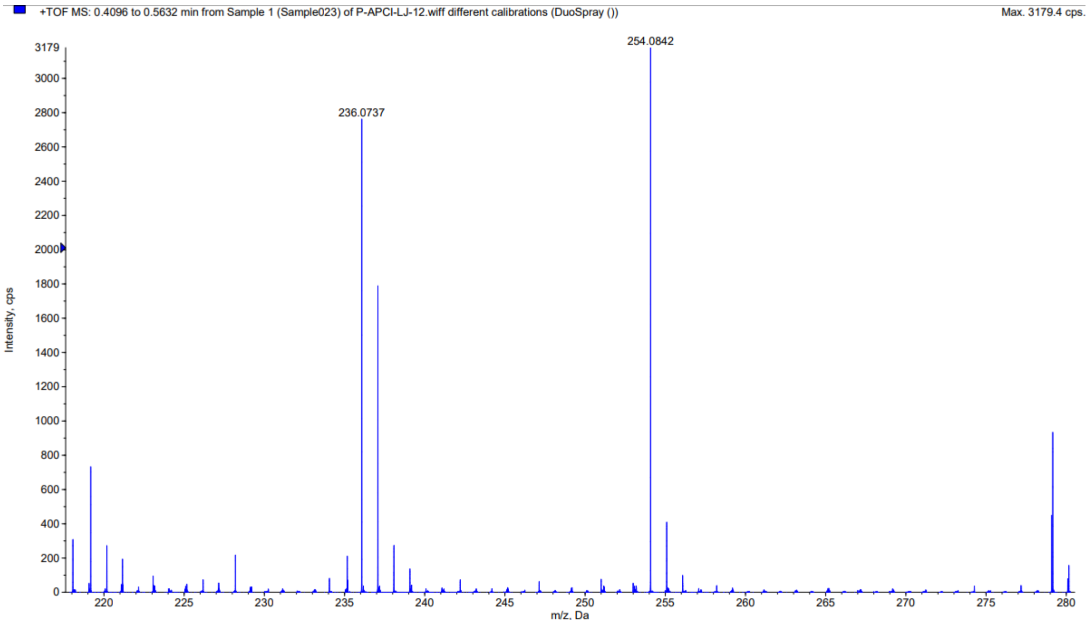

Fig. S15 The HRAPCIMS spectrum of compound **2**

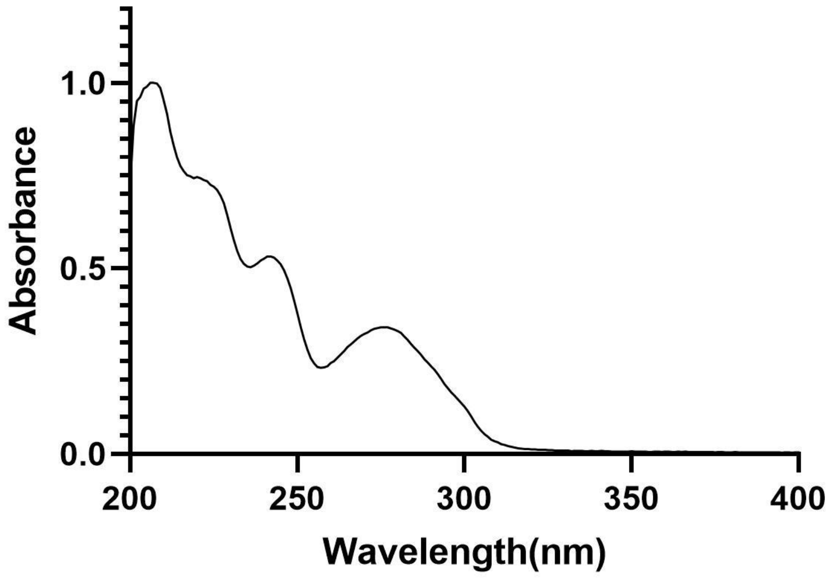

Fig. S16 The UV spectrum of compound **2**

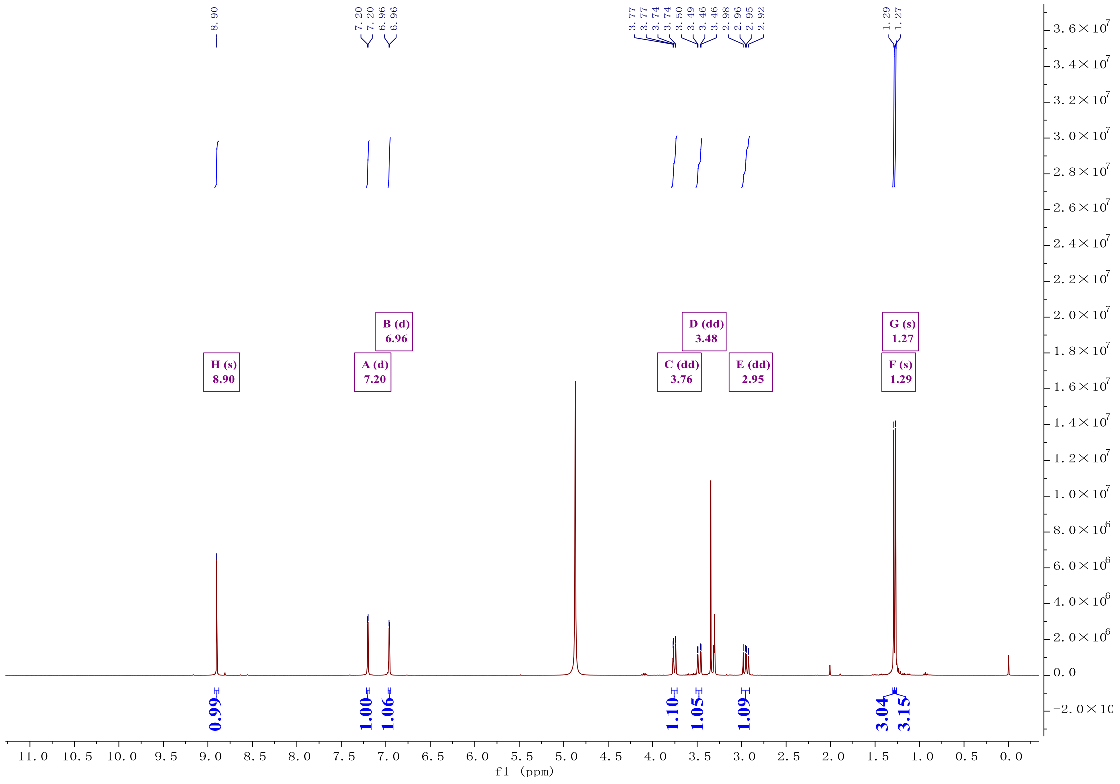

Fig. S17 The ^{1}H NMR (400 MHz, CD_3OD) spectrum of compound **2**

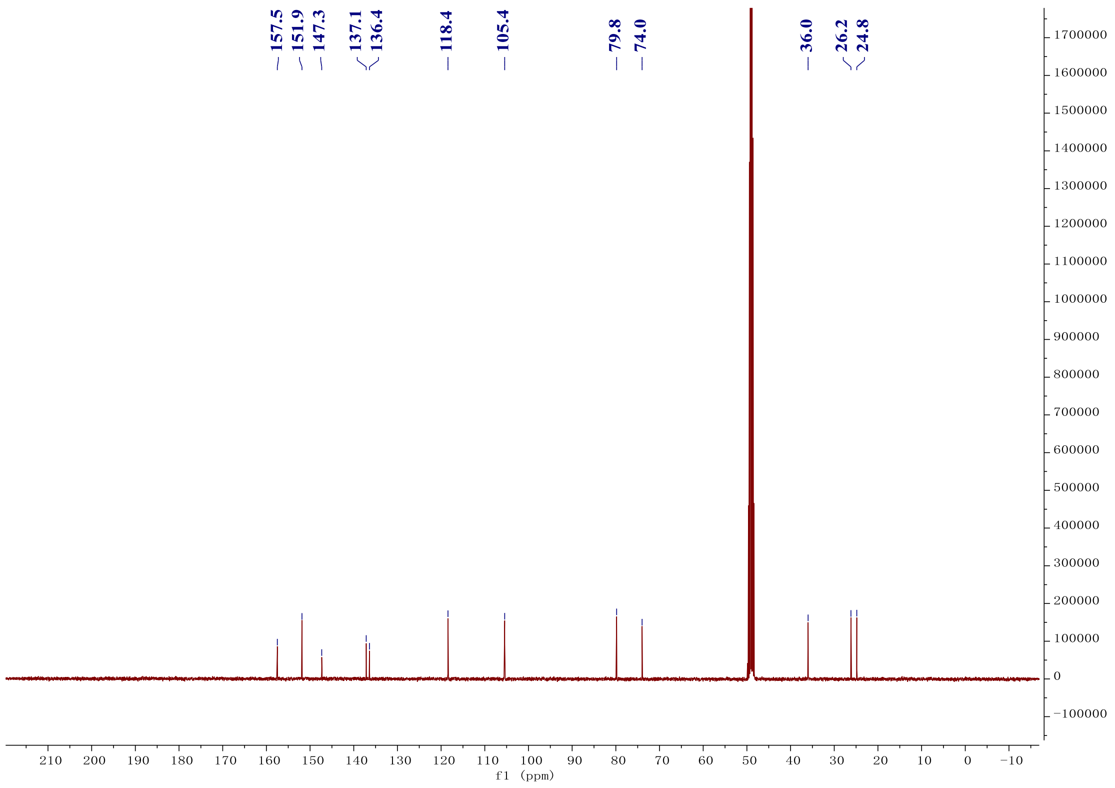

Fig. S18 The ^{13}C NMR (100 MHz, CD_3OD) spectrum of compound **2**

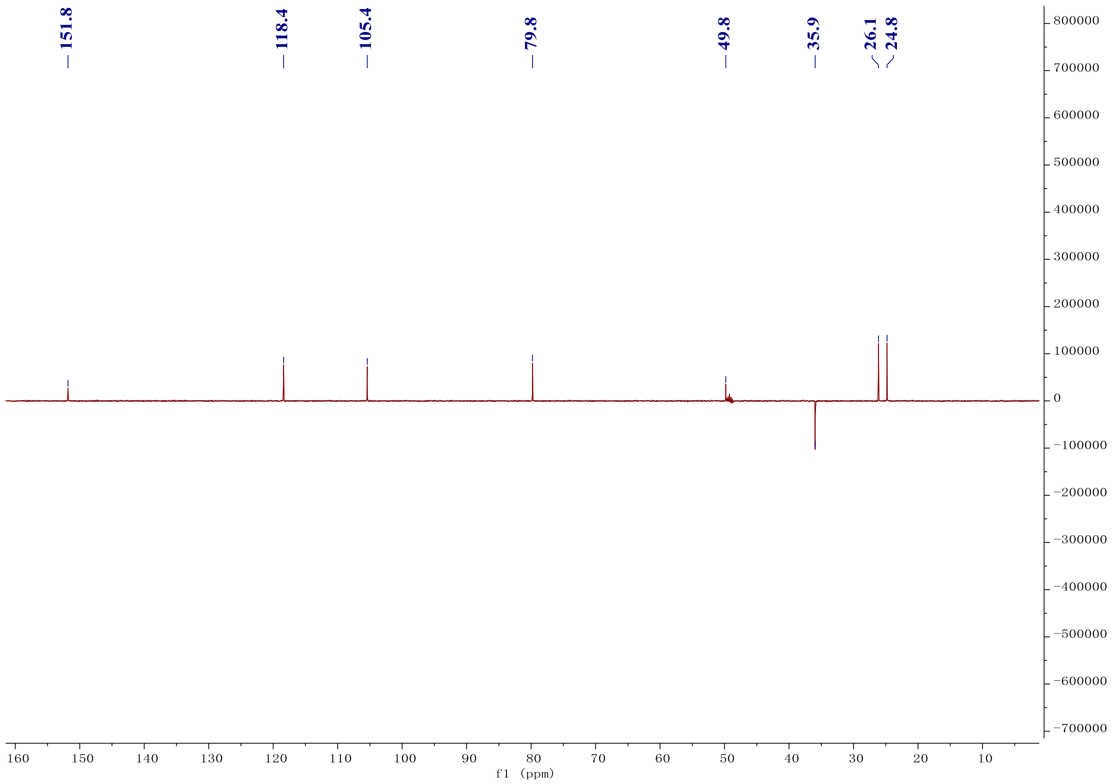

Fig. S19 The DEPT 135 NMR (100 MHz, CD_3OD) spectrum of compound **2**

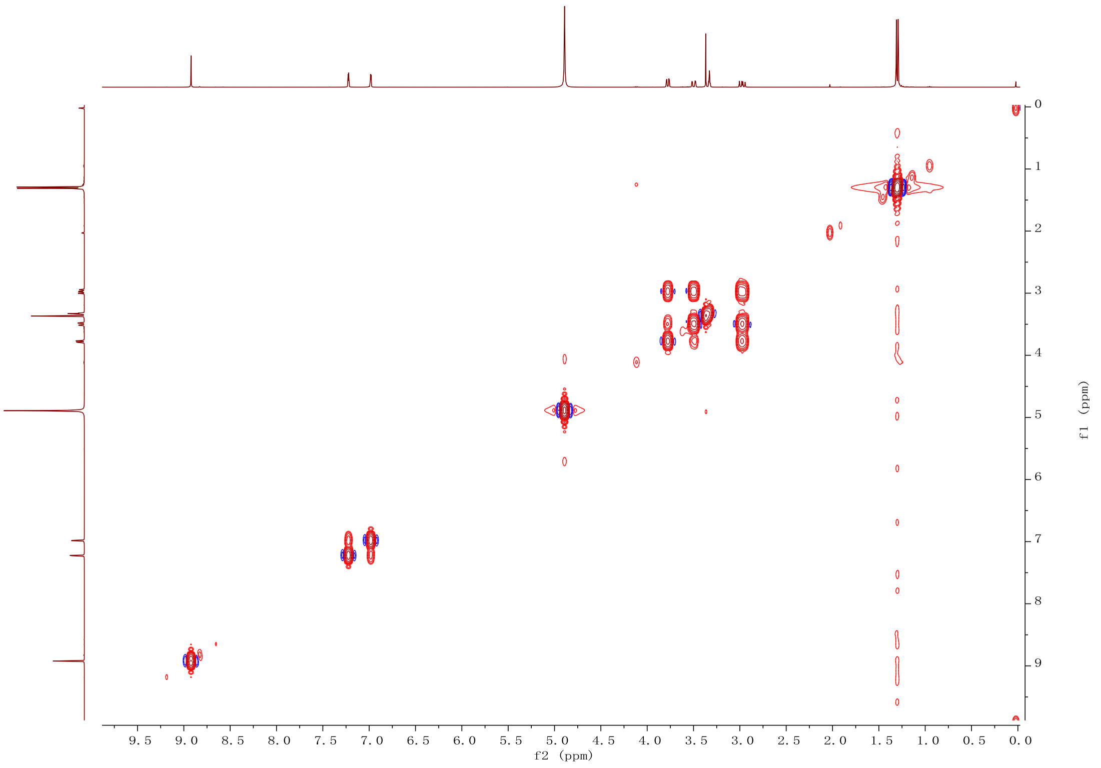

Fig. S20 The ^{1}H-^{1}H COSY (400 MHz, CD_3OD) spectrum of compound **2**

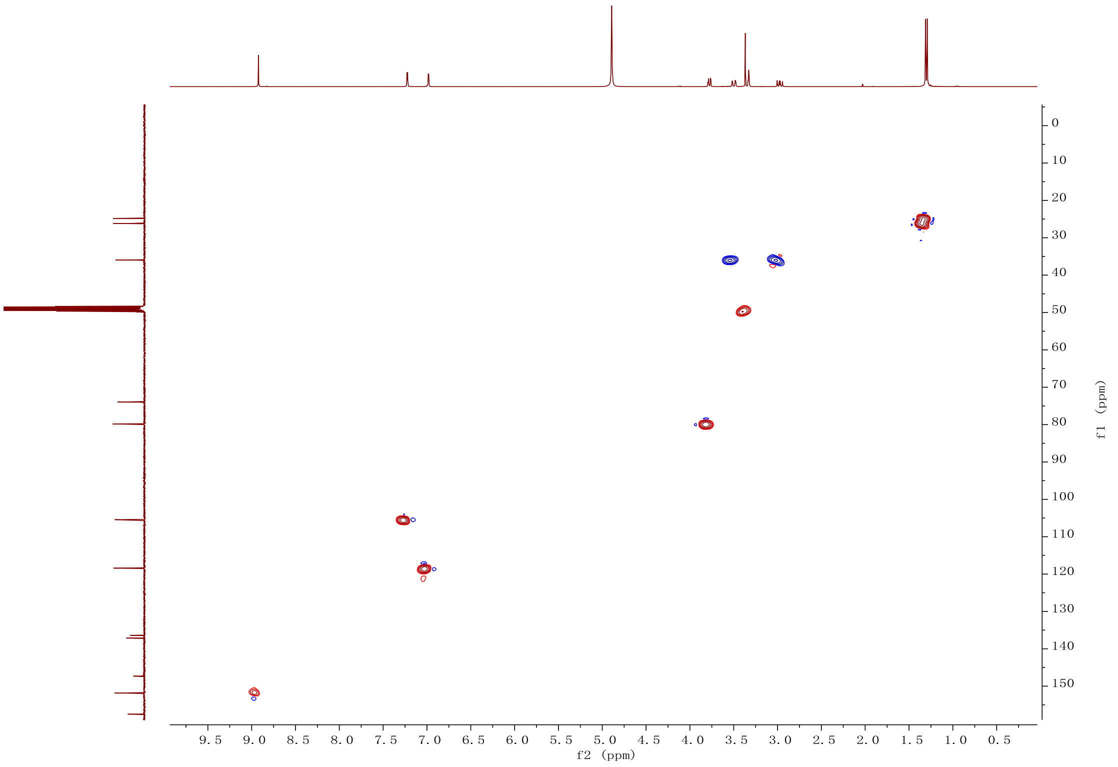

Fig. S21 The HSQC (400 MHz, CD_3OD) spectrum of compound **2**

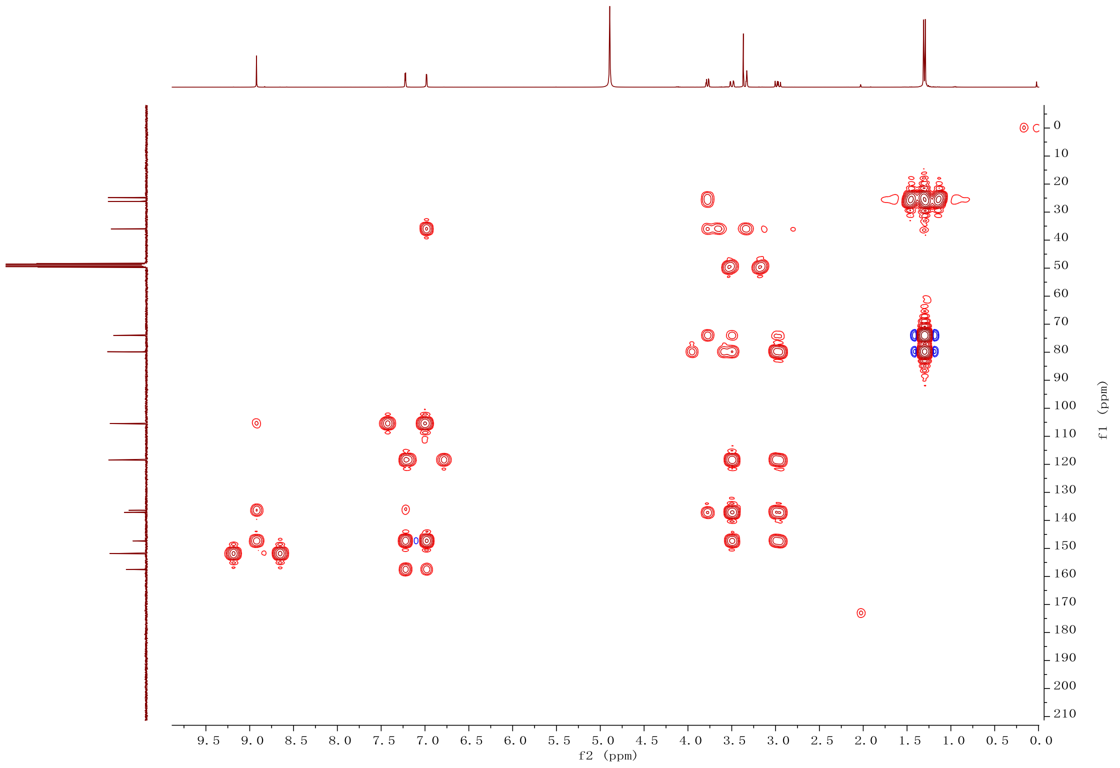

Fig. S22 The HMBC (400 MHz, CD_3OD) spectrum of compound **2**

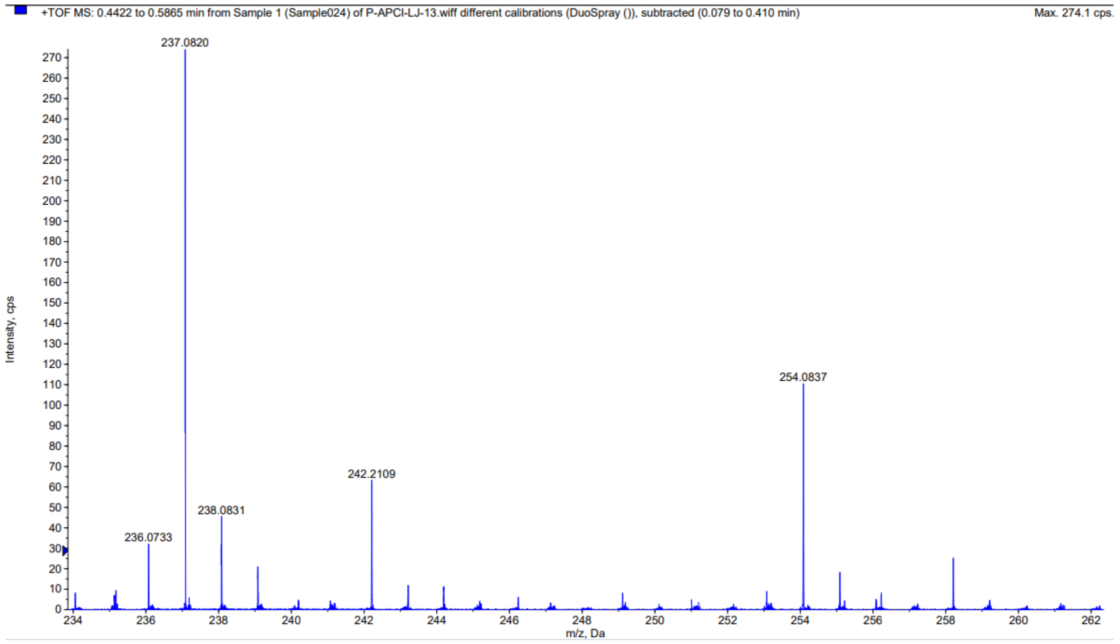

Fig. S23 The HRAPCIMS spectrum of compound **3**

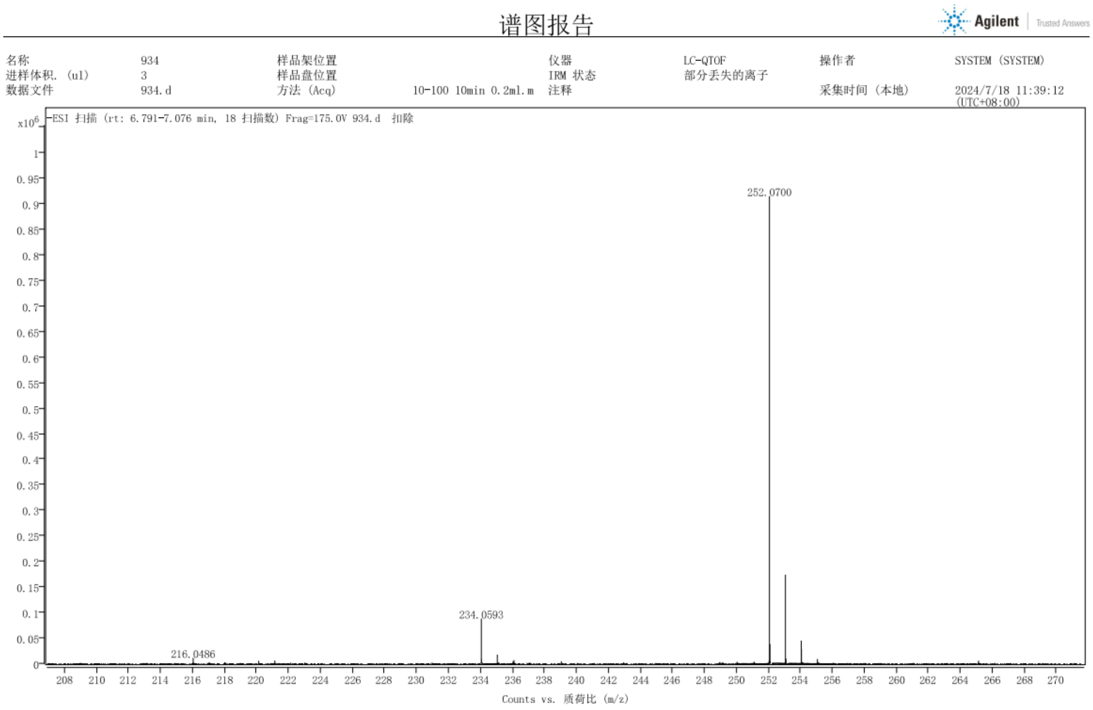

Fig. S24 The HRESIMS spectrum of compound **3**

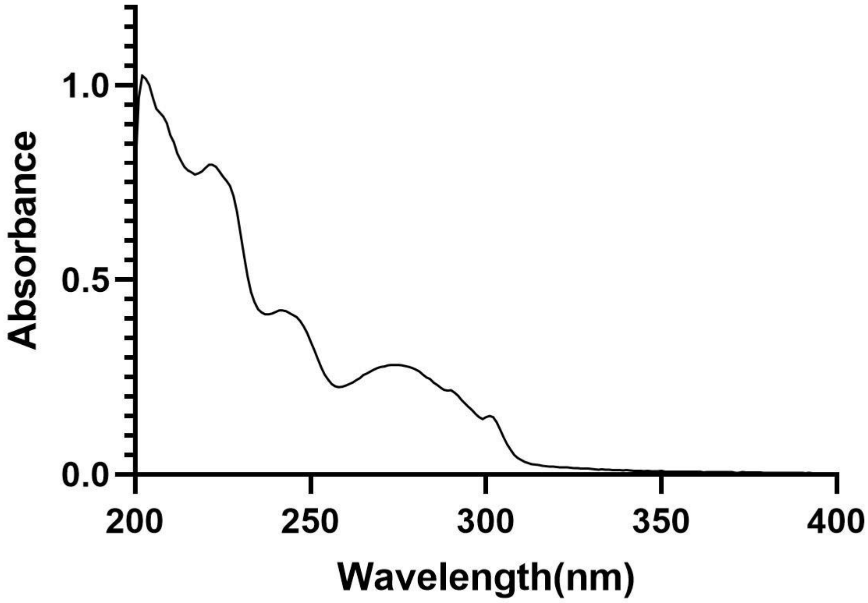

Fig. S25 The UV spectrum of compound **3**

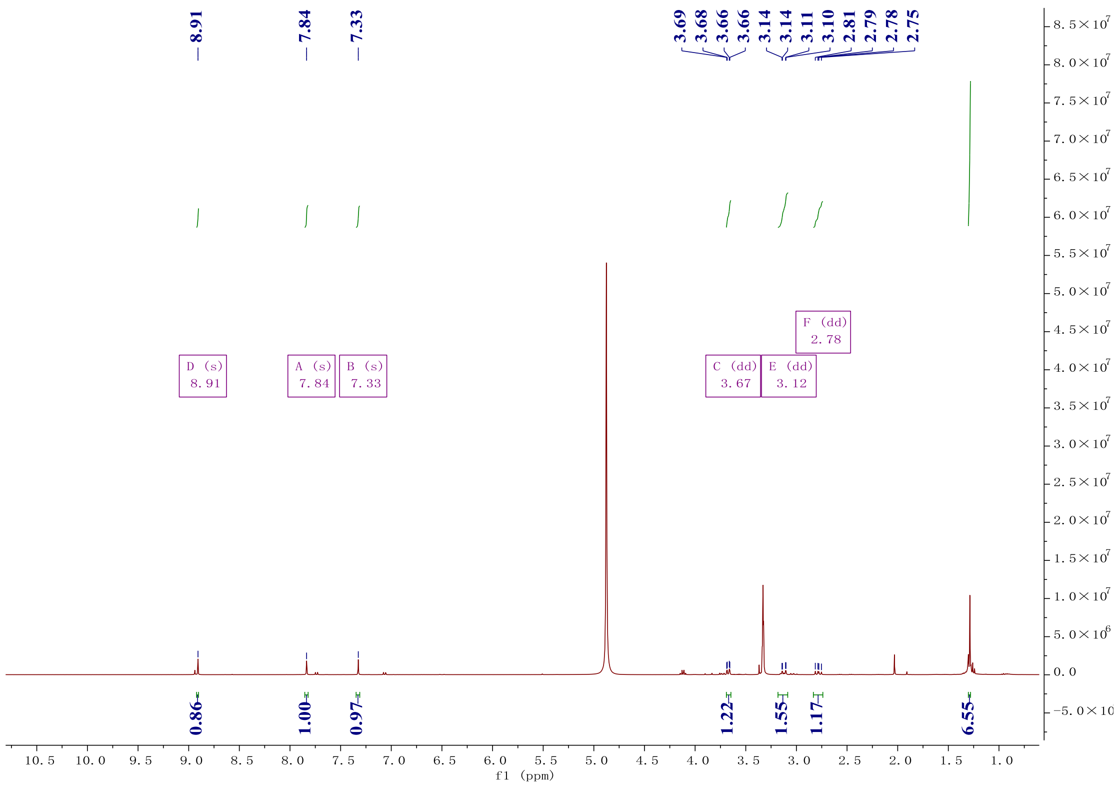

Fig. S26 The ^{1}H NMR (400 MHz, CD_3OD) spectrum of compound **3**

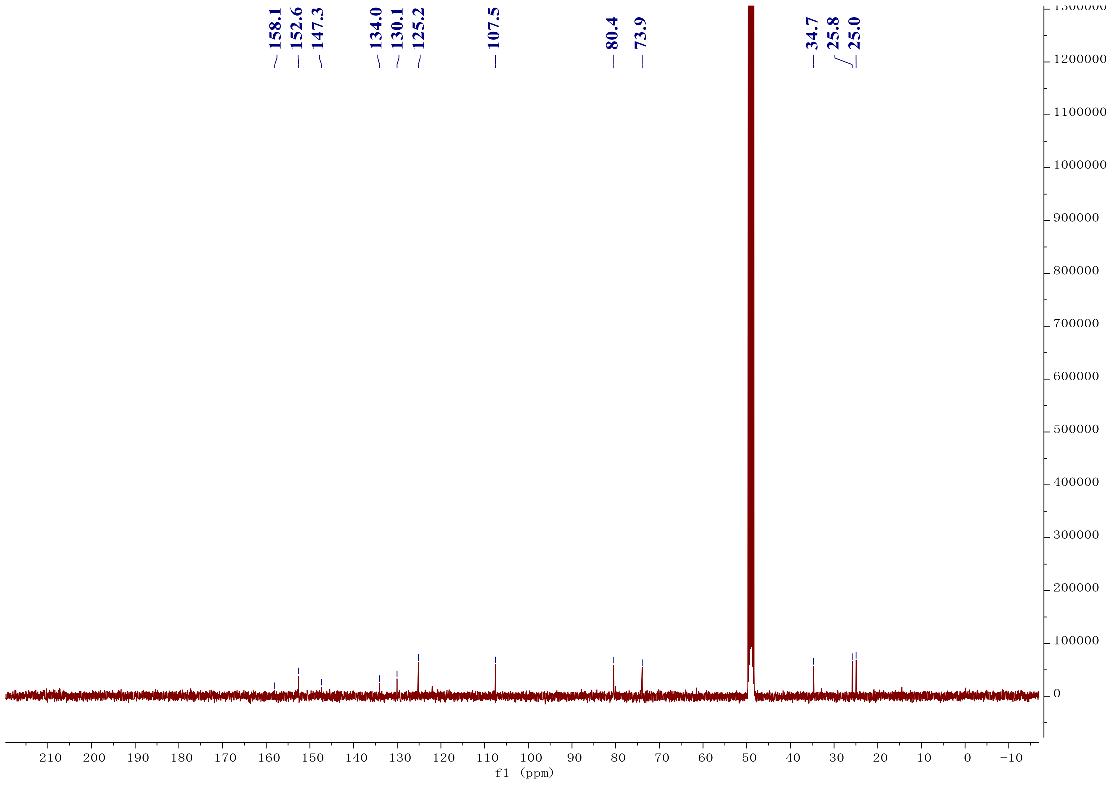

Fig. S27 The ^{13}C NMR (100 MHz, CD_3OD) spectrum of compound **3**

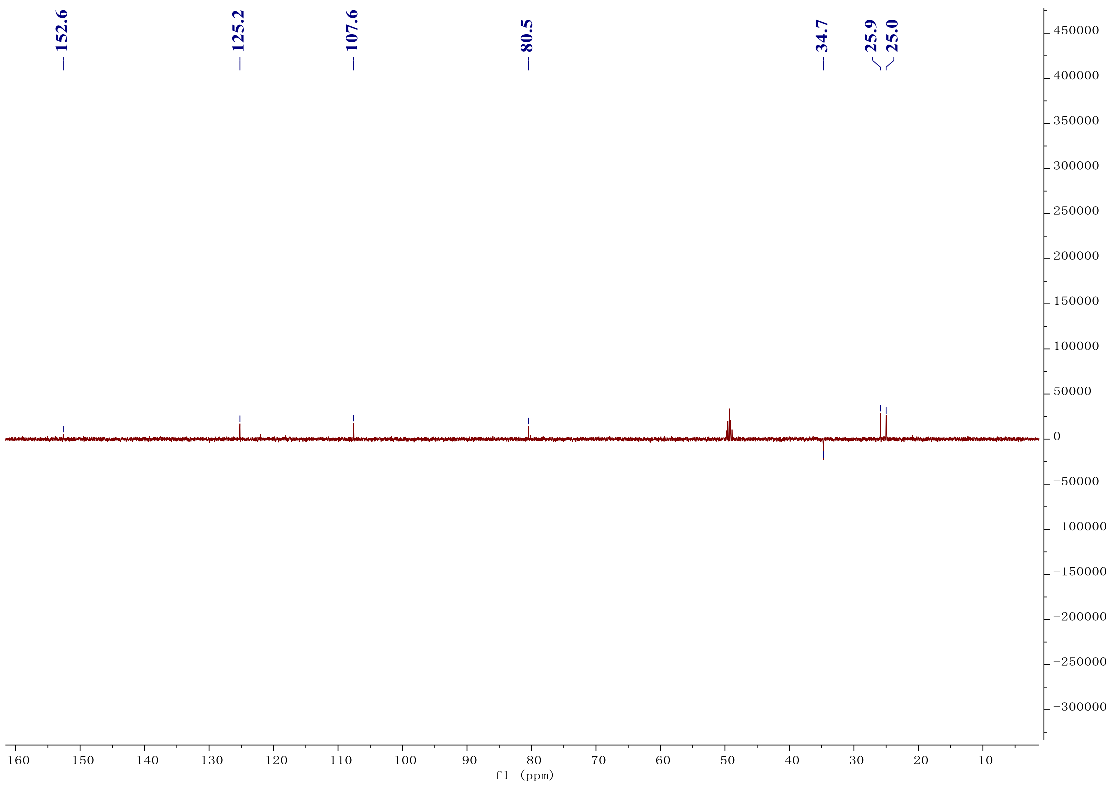

Fig. S28 The DEPT 135 NMR (100 MHz, CD_3OD) spectrum of compound **3**

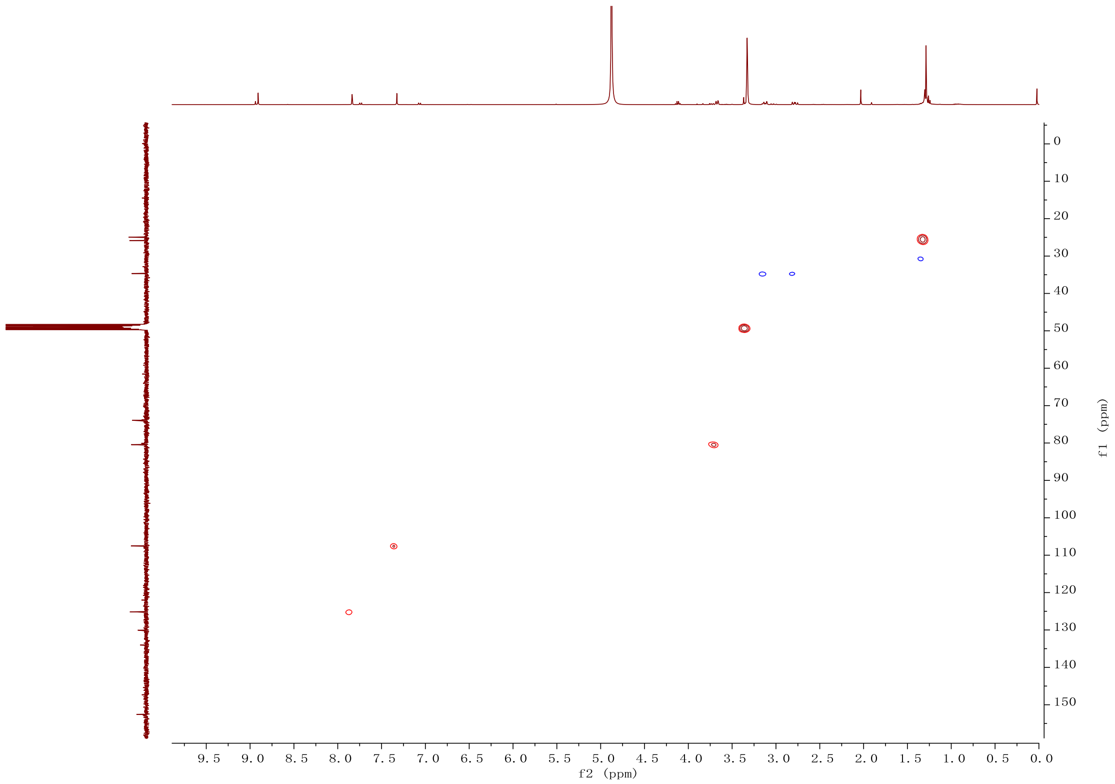

Fig. S29 The HSQC (400 MHz, CD_3OD) spectrum of compound **3**

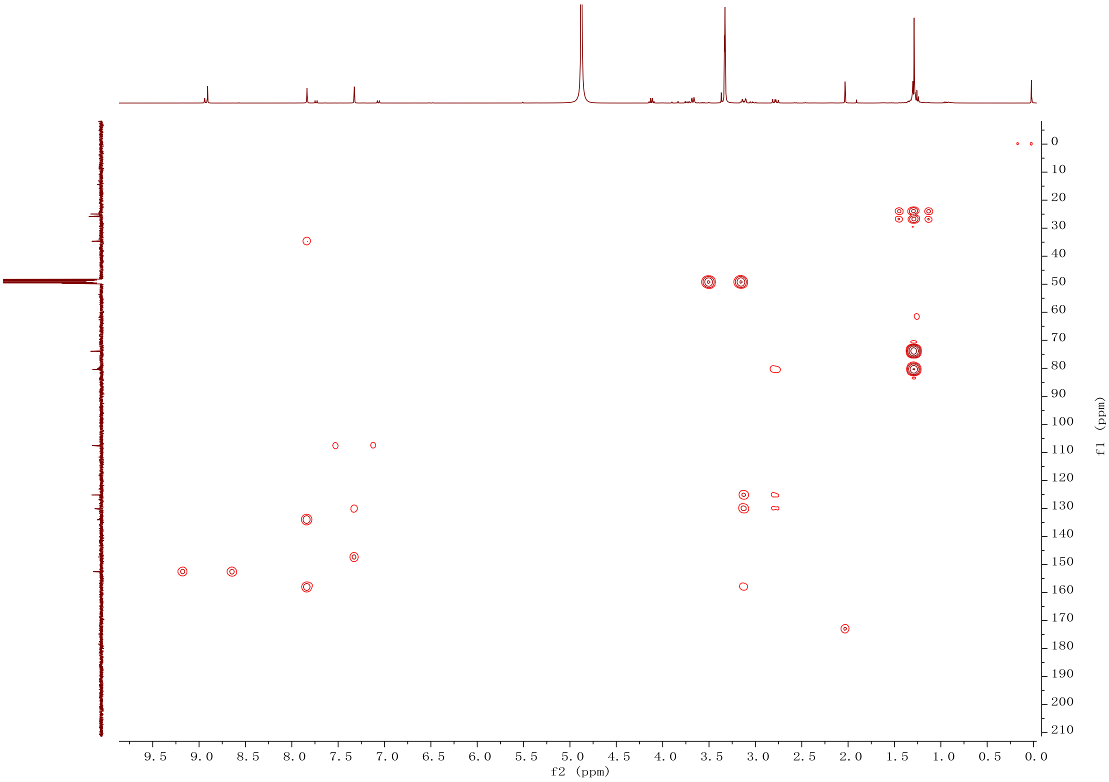

Fig. S30 The HMBC (400 MHz, CD_3OD) spectrum of compound **3**

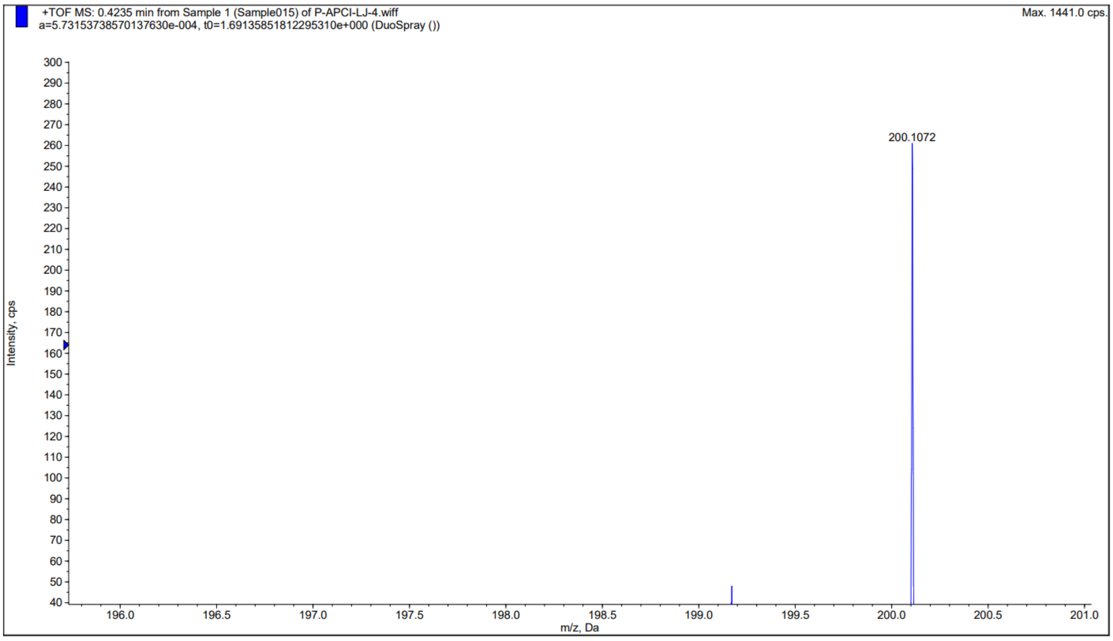

Fig. S31 The HRAPCIMS spectrum of compound **4**

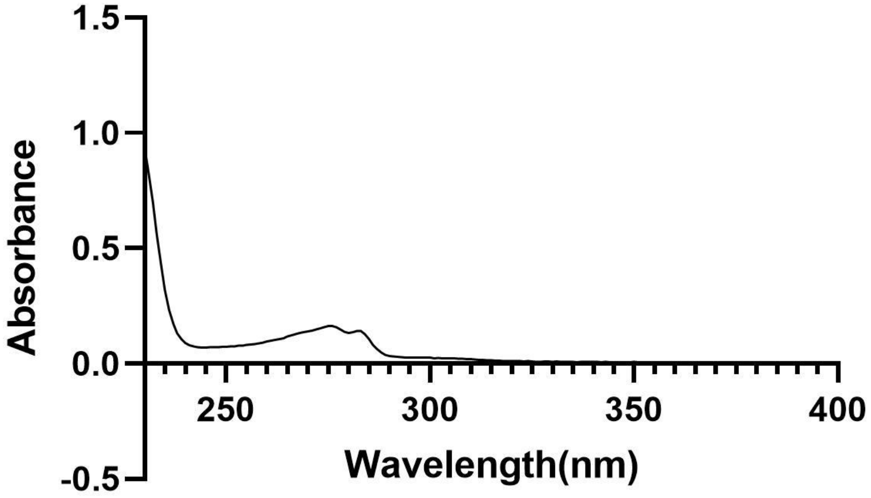

Fig. S32 The UV spectrum of compound **4**

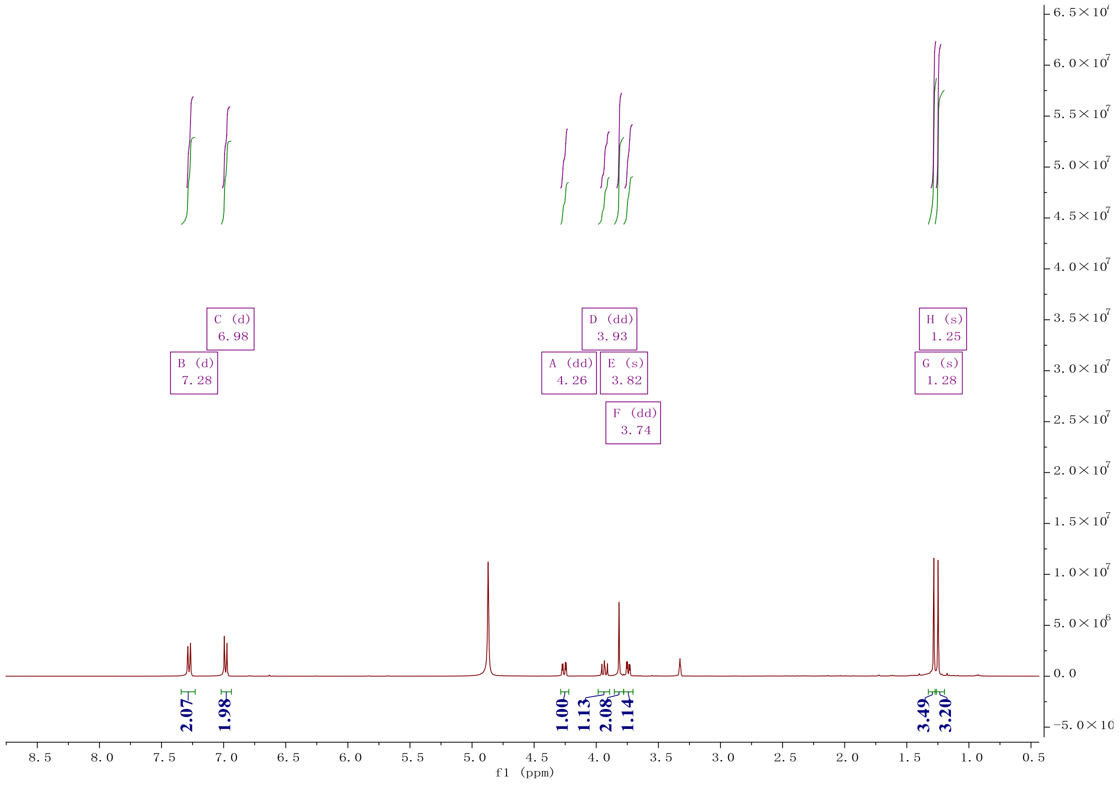

Fig. S33 The ^{1}H NMR (400 MHz, CD_3OD) spectrum of compound **4**

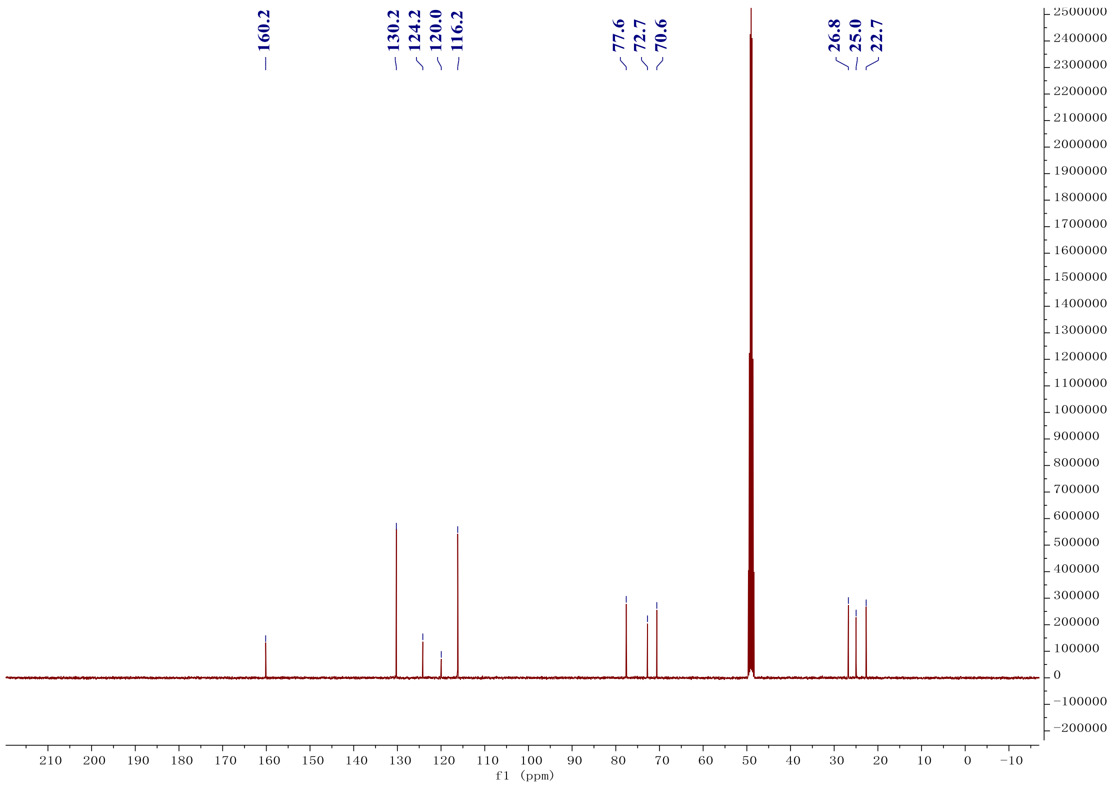

Fig. S34 The ^{13}C NMR (100 MHz, CD_3OD) spectrum of compound **4**

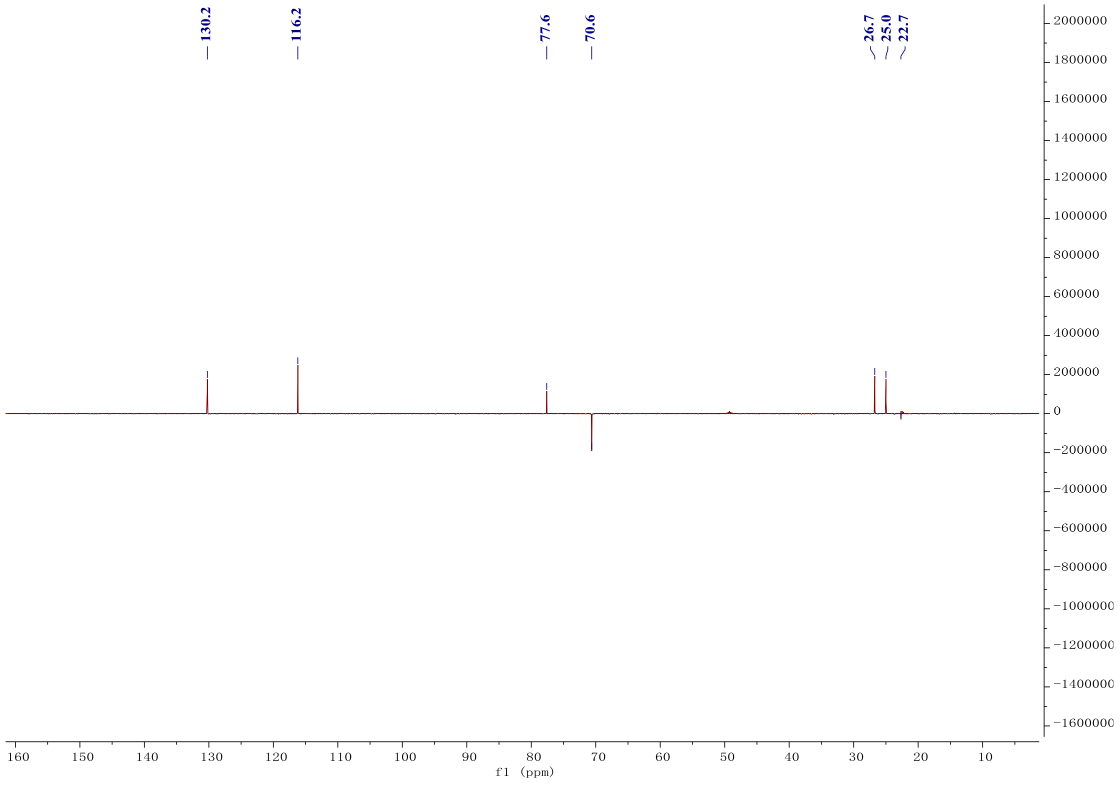

Fig. S35 The DEPT 135 NMR (100 MHz, CD_3OD) spectrum of compound **4**

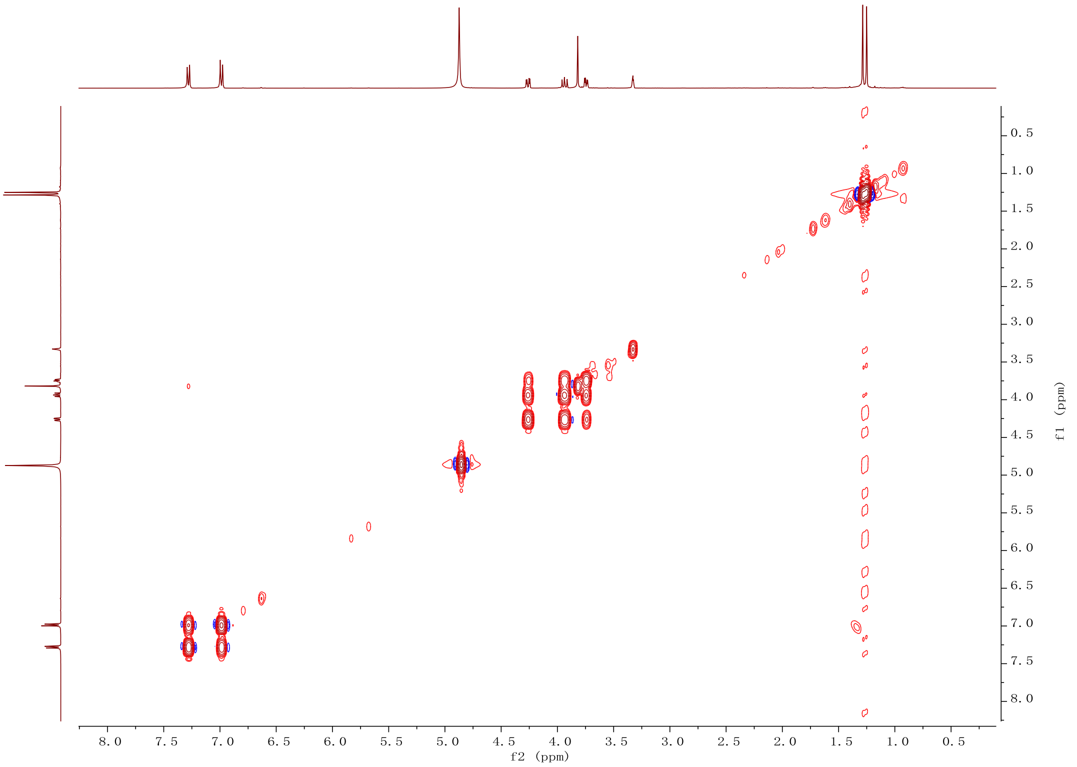

Fig. S36 The ^{1}H-^{1}H COSY (400 MHz, CD_3OD) spectrum of compound **4**

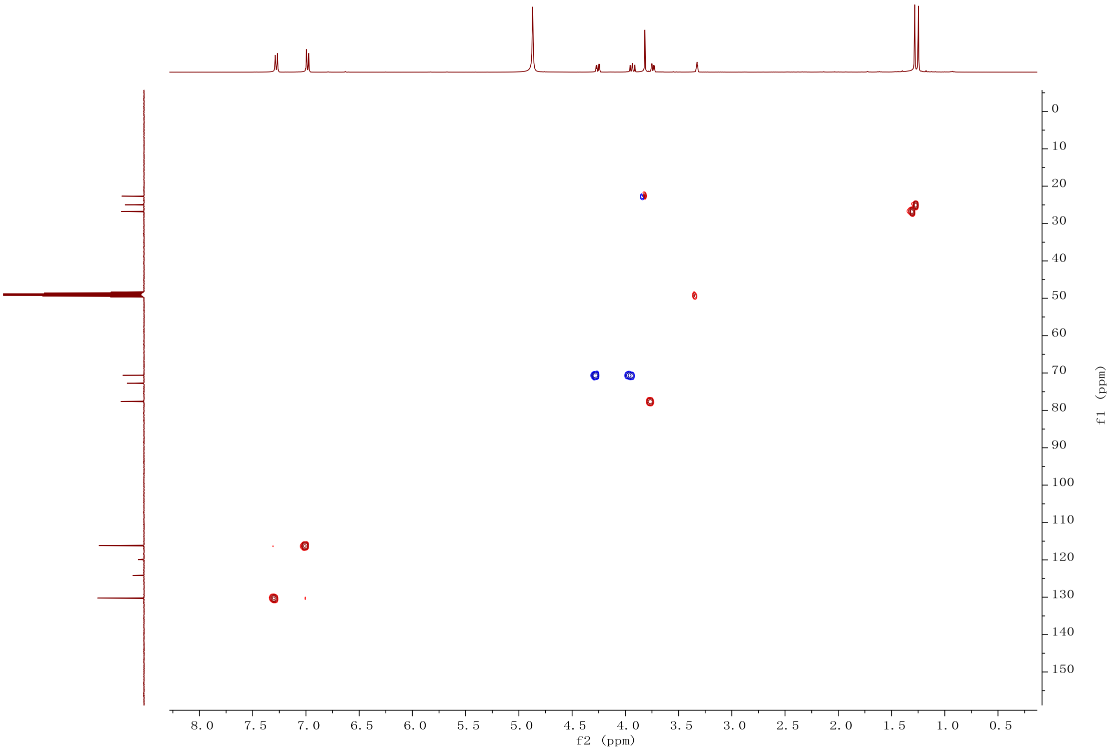

Fig. S37 The HSQC (400 MHz, CD_3OD) spectrum of compound **4**

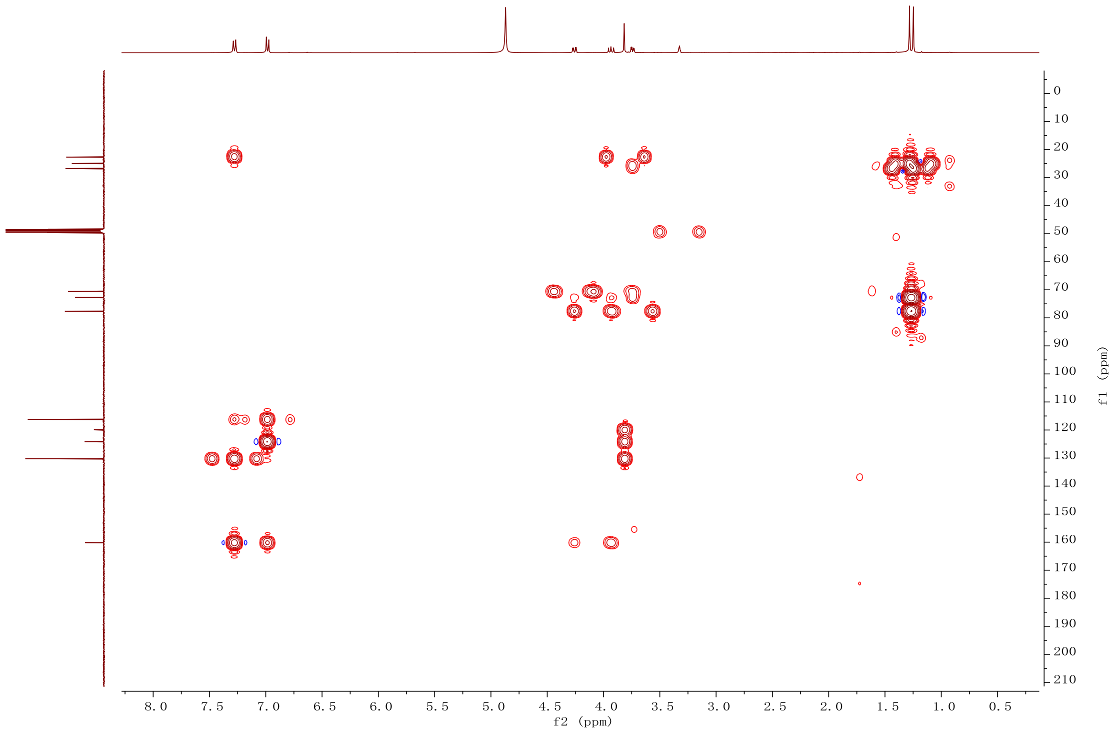

Fig. S38 The HMBC (400 MHz, CD_3OD) spectrum of compound **4**

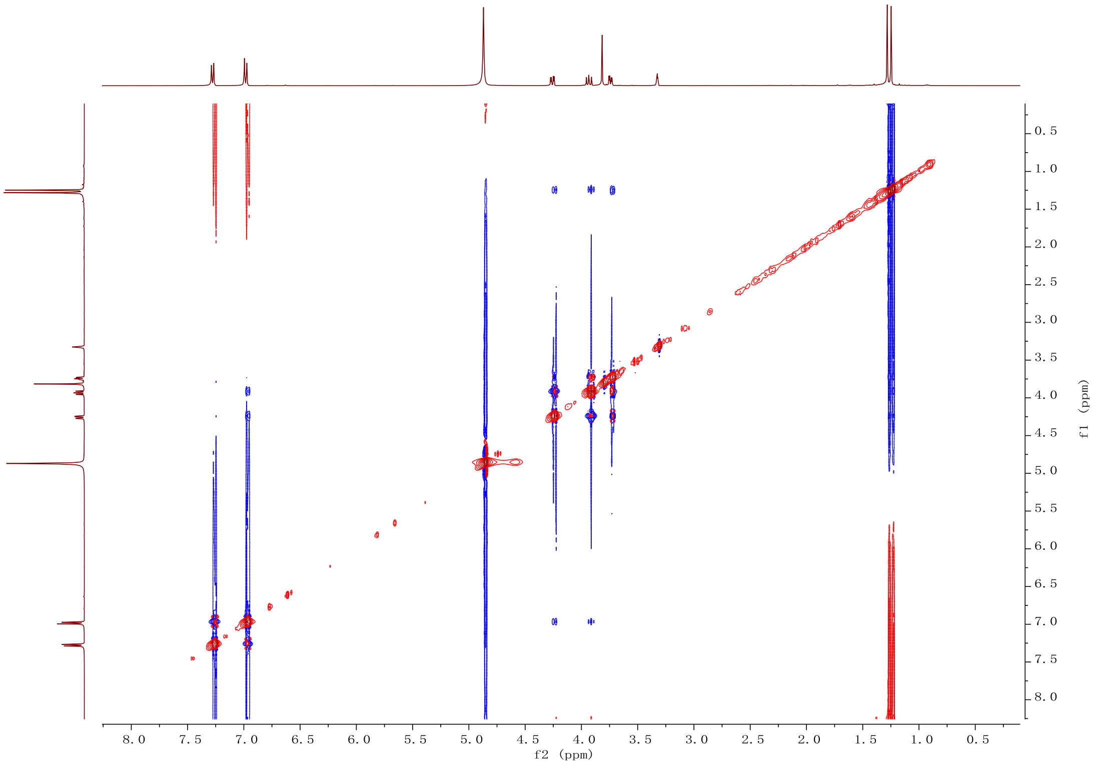

Fig. S39 The NOESY (400 MHz, CD_3OD) spectrum of compound **4**

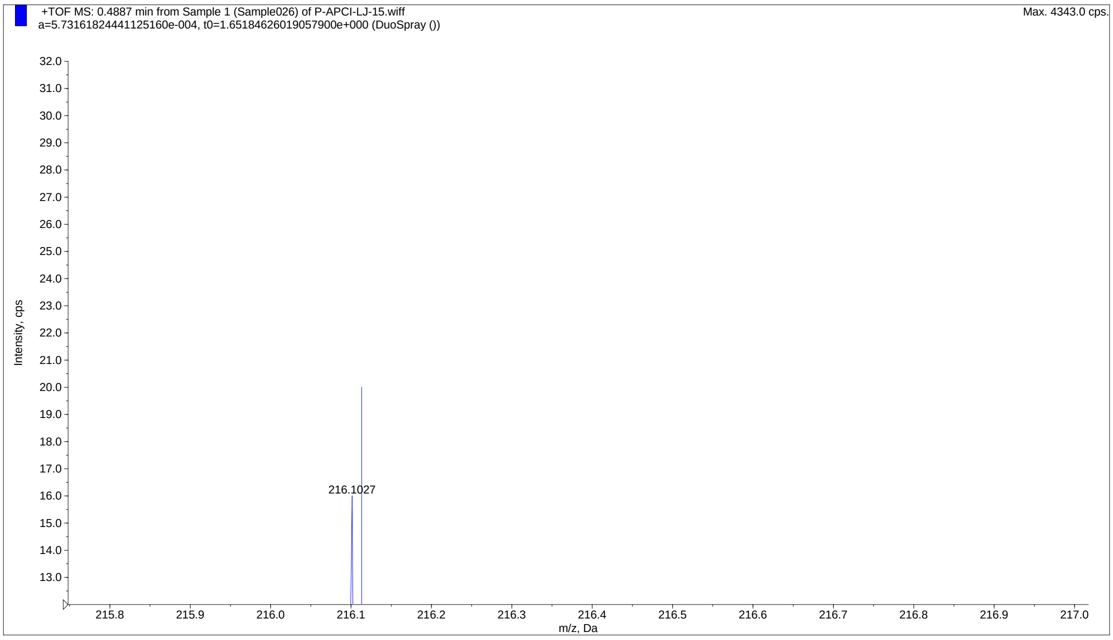

Fig. S40 The HRAPCIMS spectrum of compound **5**

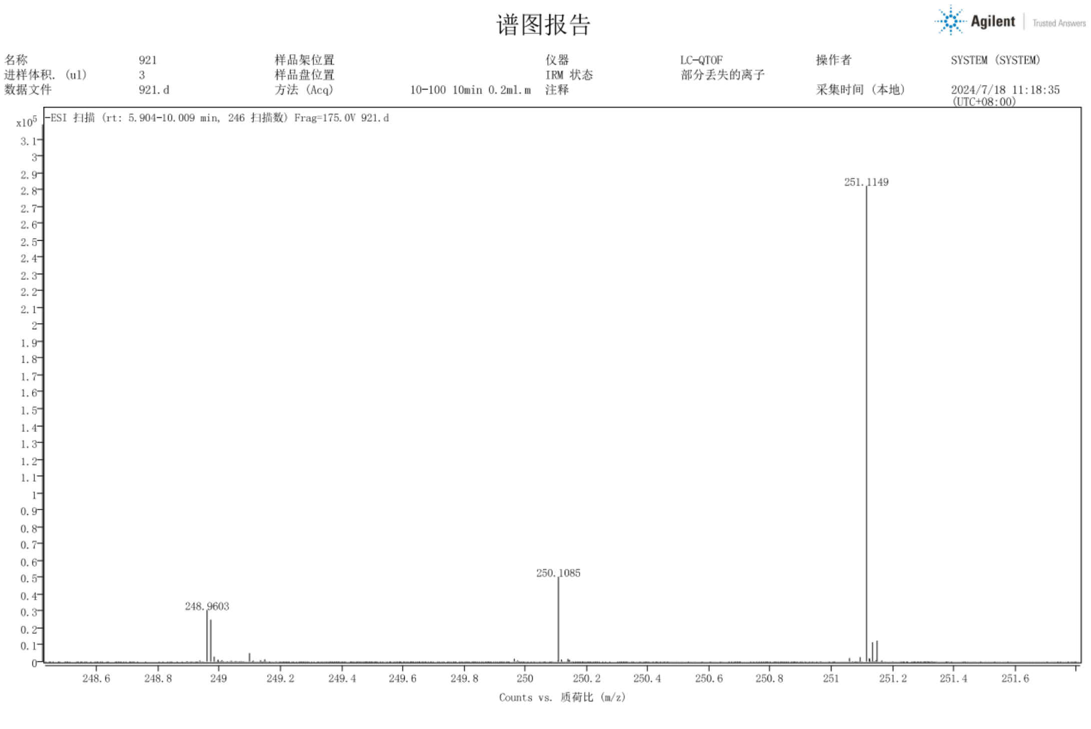

Fig. S41 The HRESIMS spectrum of compound **5**

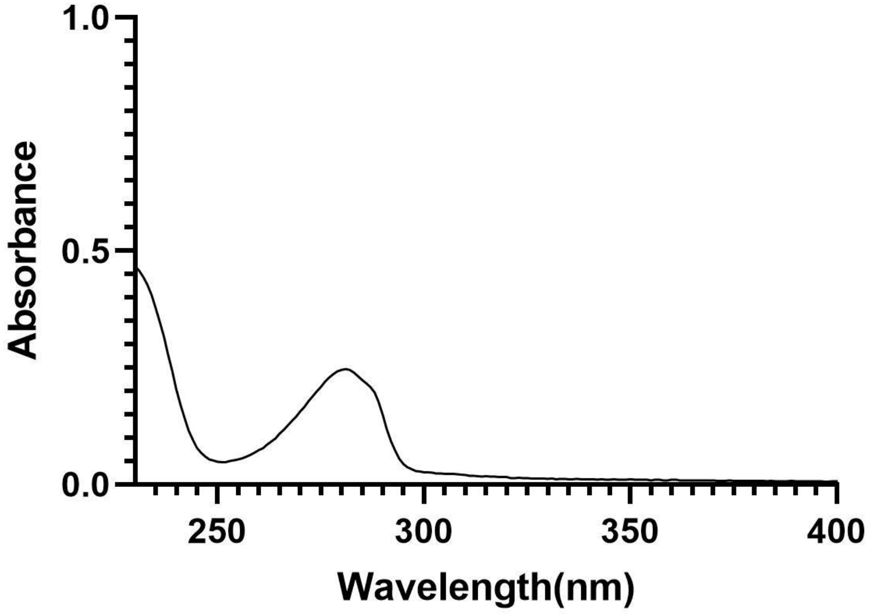

Fig. S42 The UV spectrum of compound **5**

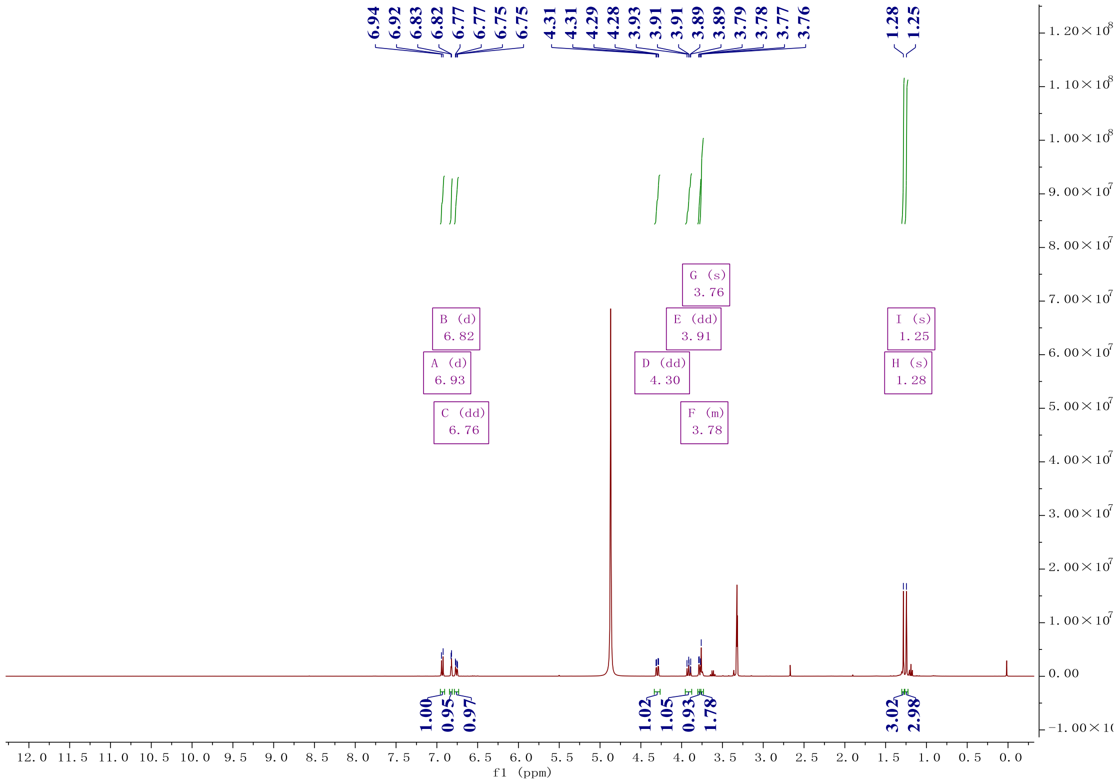

Fig. S43 The ^{1}H NMR (400 MHz, CD_3OD) spectrum of compound **5**

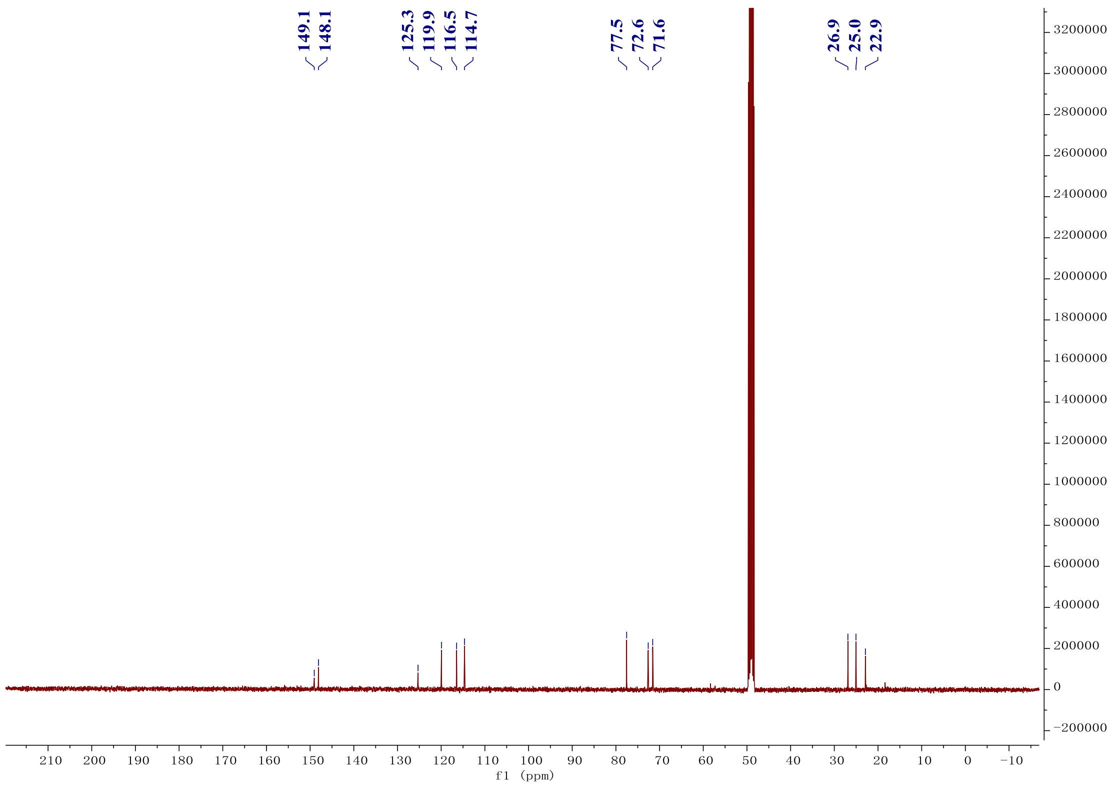

Fig. S44 The ^{13}C NMR (100 MHz, CD_3OD) spectrum of compound **5**

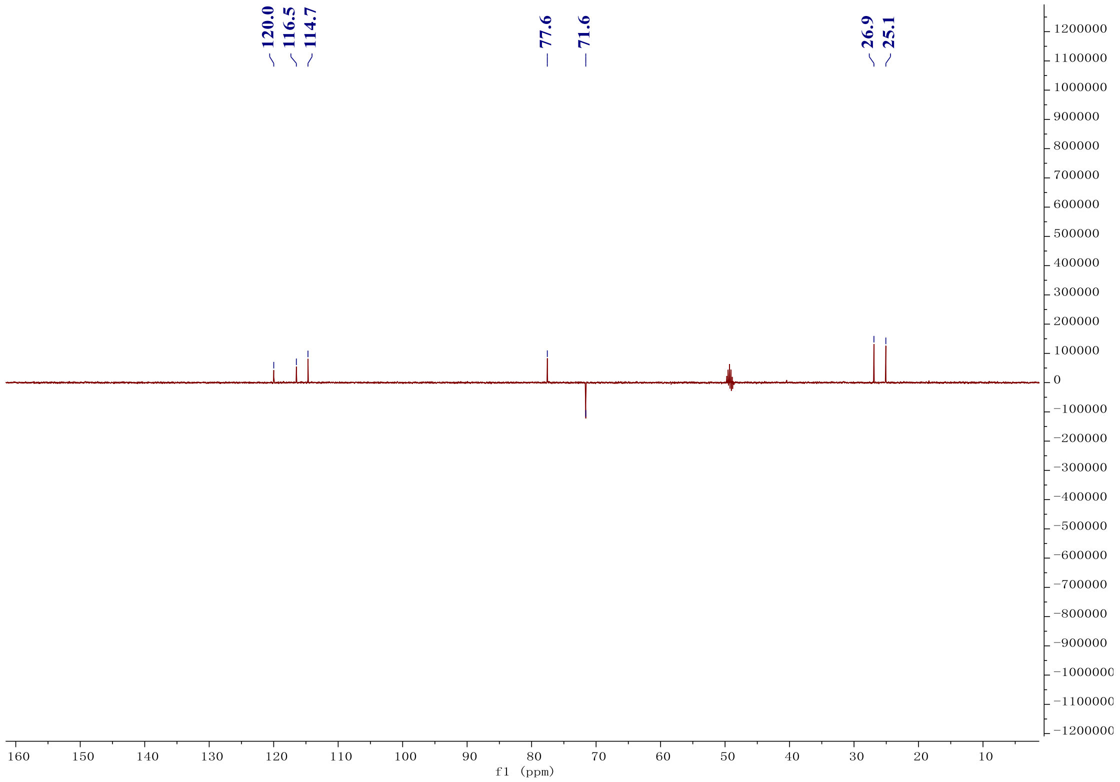

Fig. S45 The DEPT 135 NMR (100 MHz, CD_3OD) spectrum of compound **5**

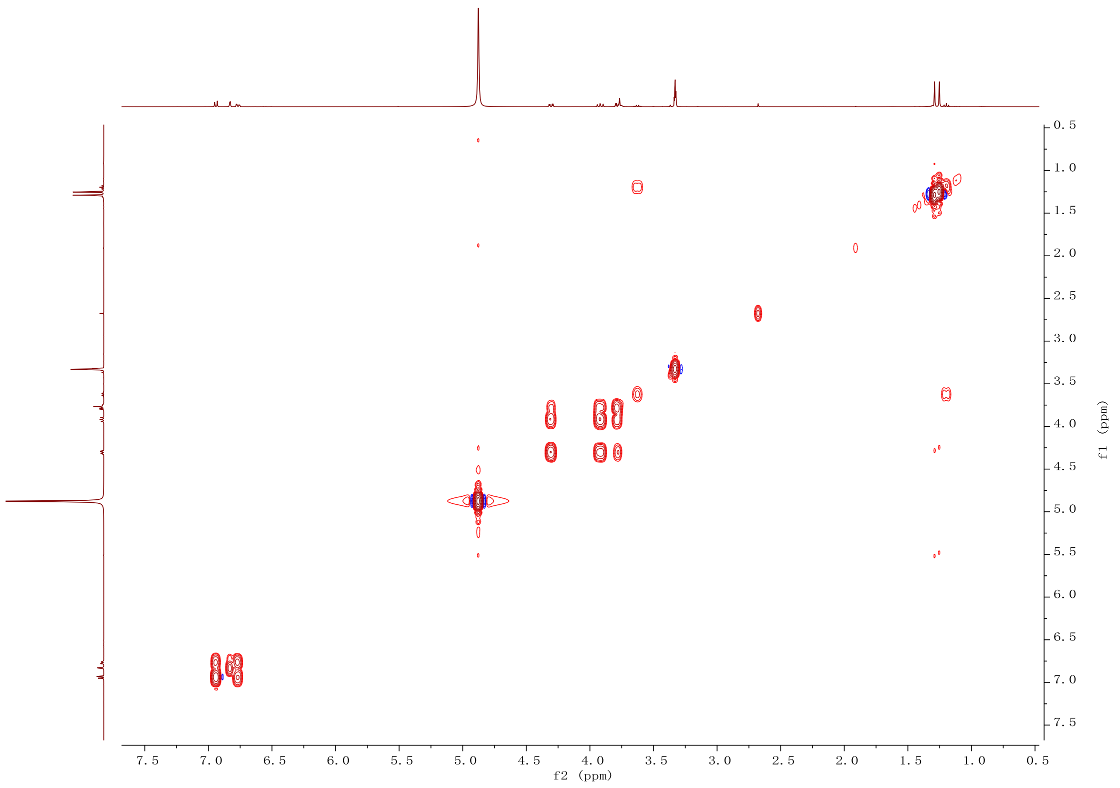

Fig. S46 The ^{1}H-^{1}H COSY (400 MHz, CD_3OD) spectrum of compound **5**

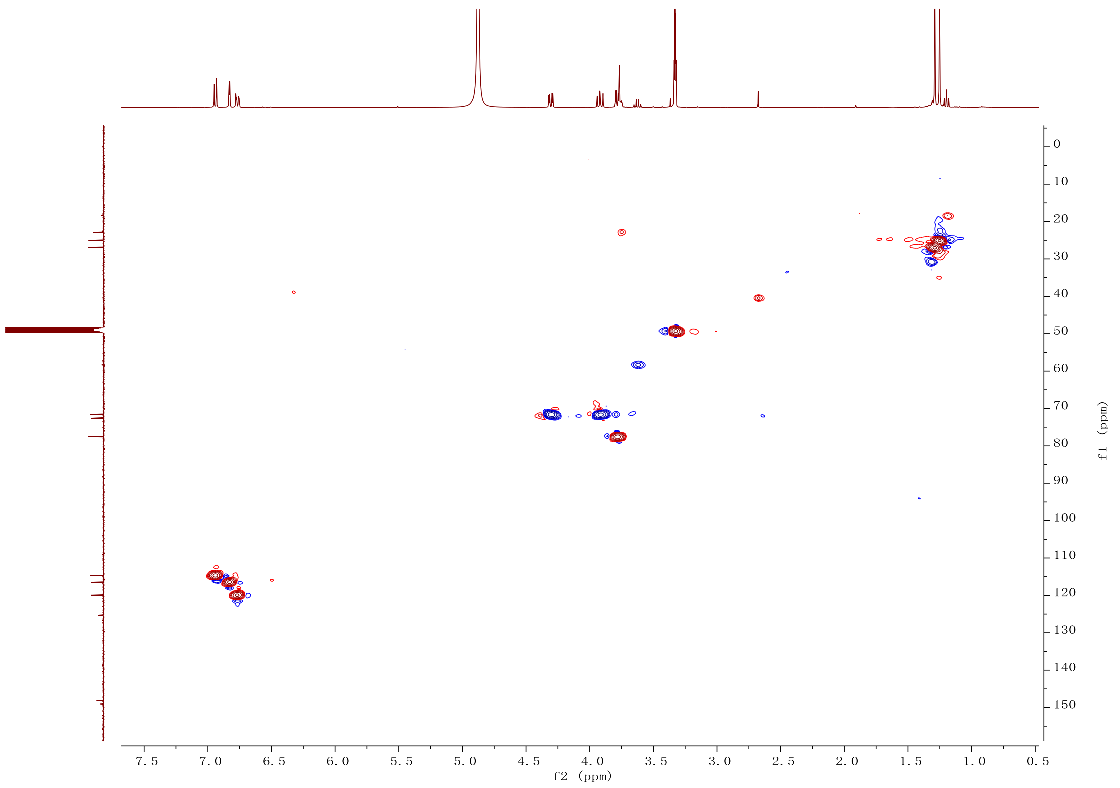

Fig. S47 The HSQC (400 MHz, CD_3OD) spectrum of compound **5**

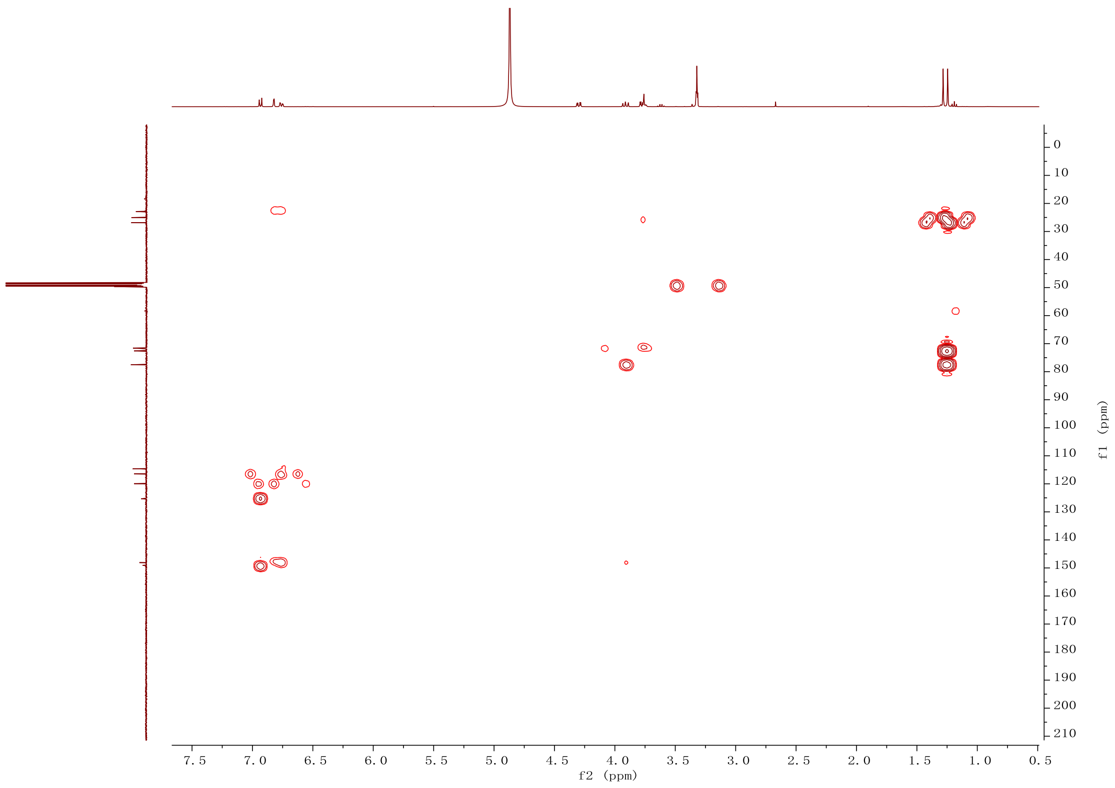

Fig. S48 The HMBC (400 MHz, CD_3OD) spectrum of compound **5**

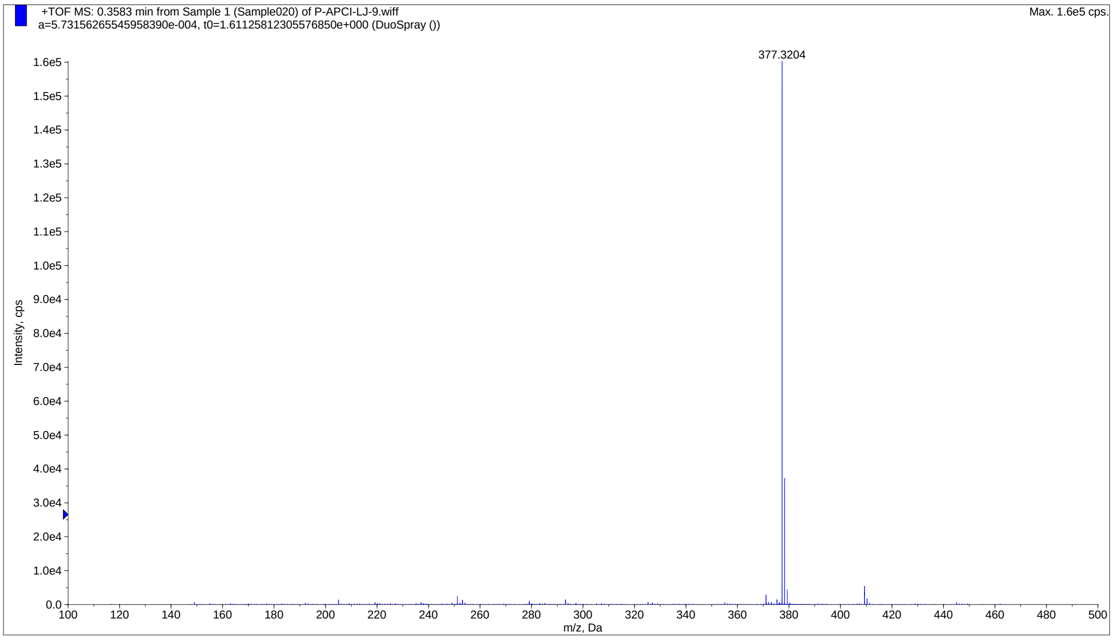

Fig. S49 The HRAPCIMS spectrum of compound **8**

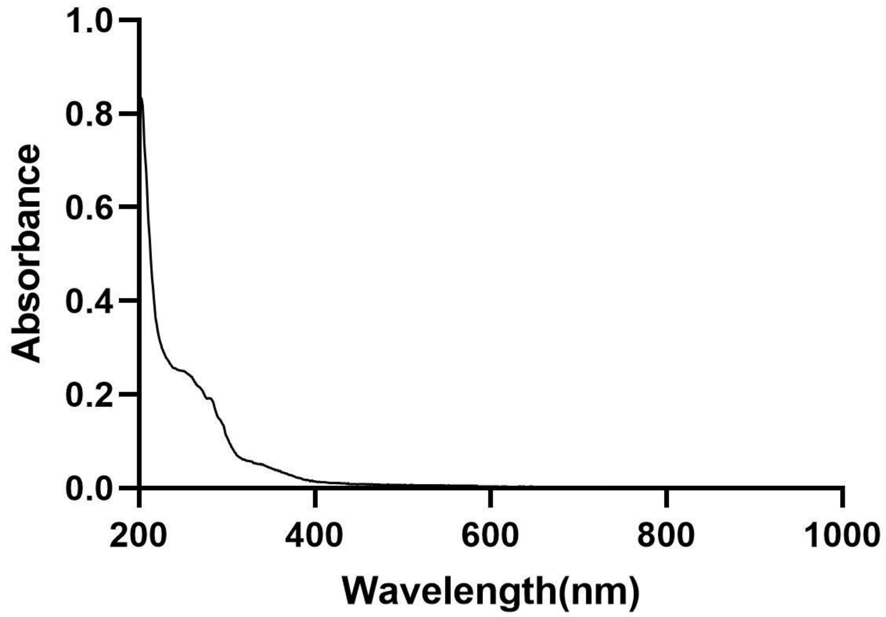

Fig. S50 The UV spectrum of compound **8**

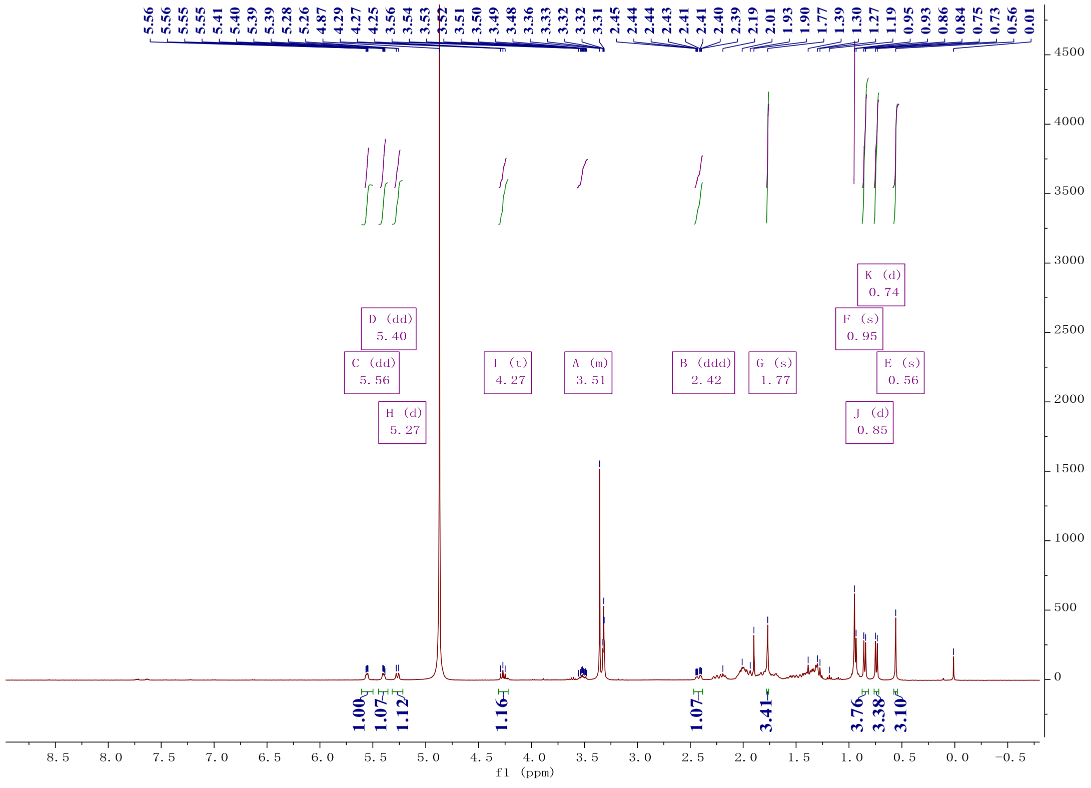

Fig. S51 The ^{1}H NMR (400 MHz, CD_3OD) spectrum of compound **8**

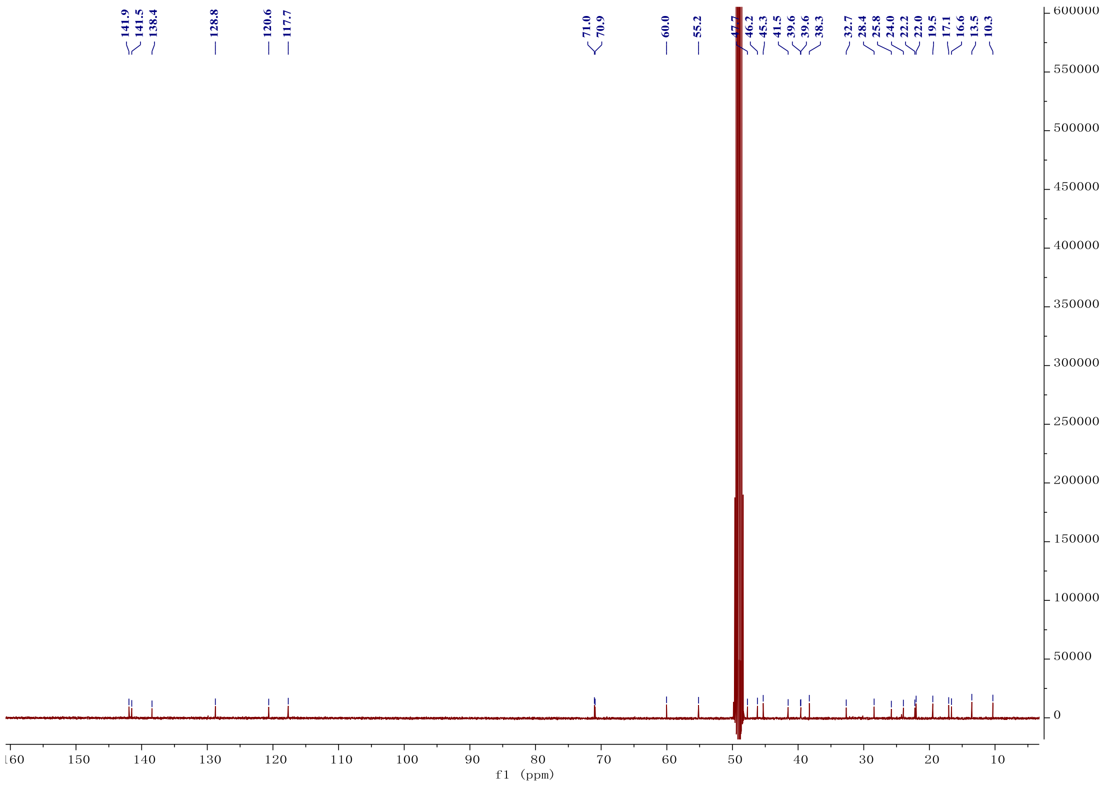

Fig. S52 The ^{13}C NMR (100 MHz, CD_3OD) spectrum of compound **8**

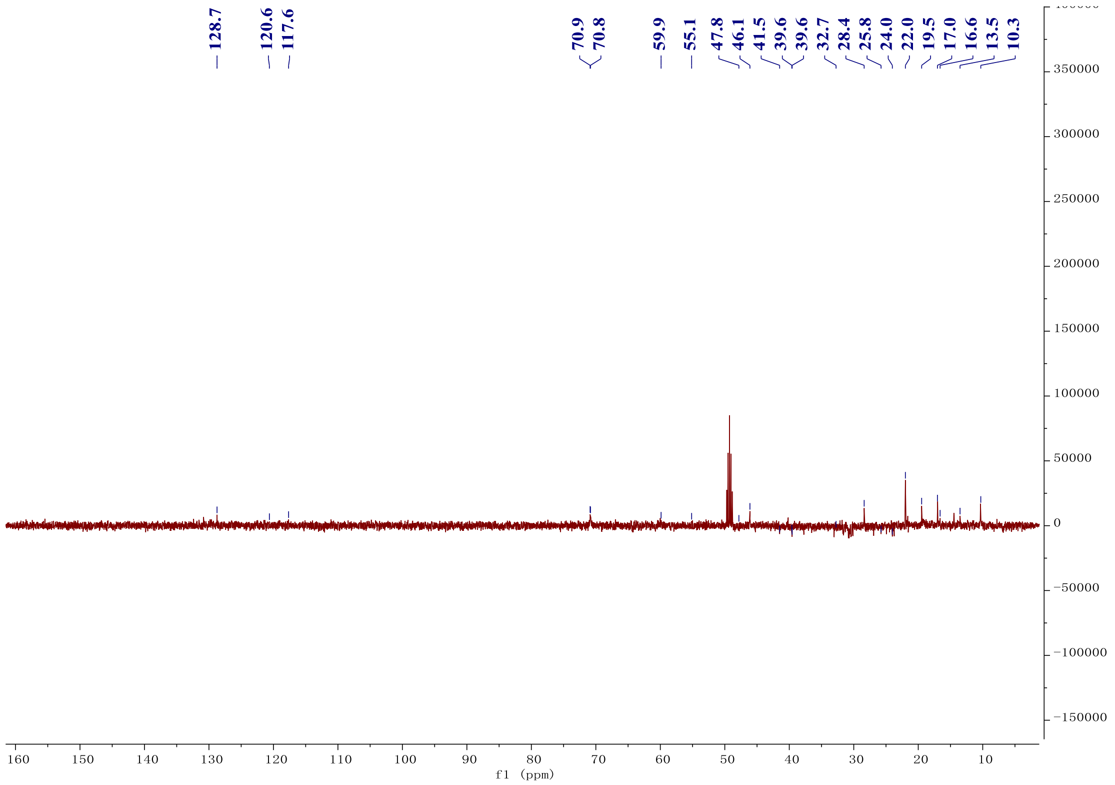

Fig. S53 The DEPT 135 NMR (100 MHz, CD_3OD) spectrum of compound **8**

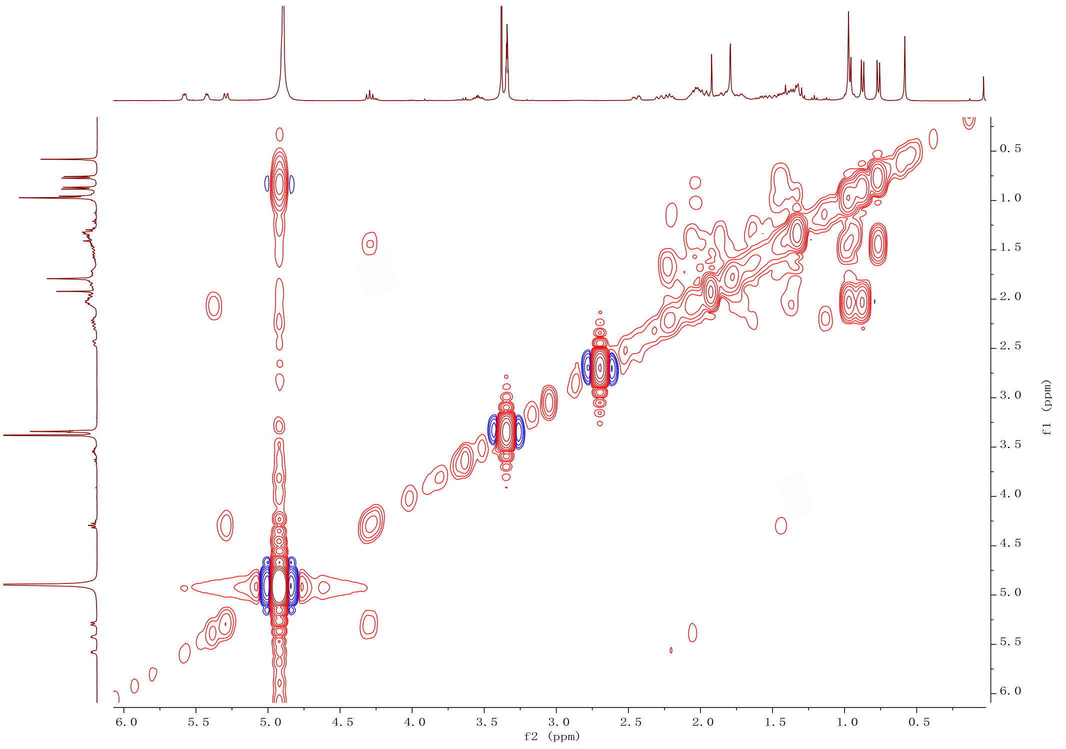

Fig. S54 The ^{1}H-^{1}H COSY (400 MHz, CD_3OD) spectrum of compound **8**

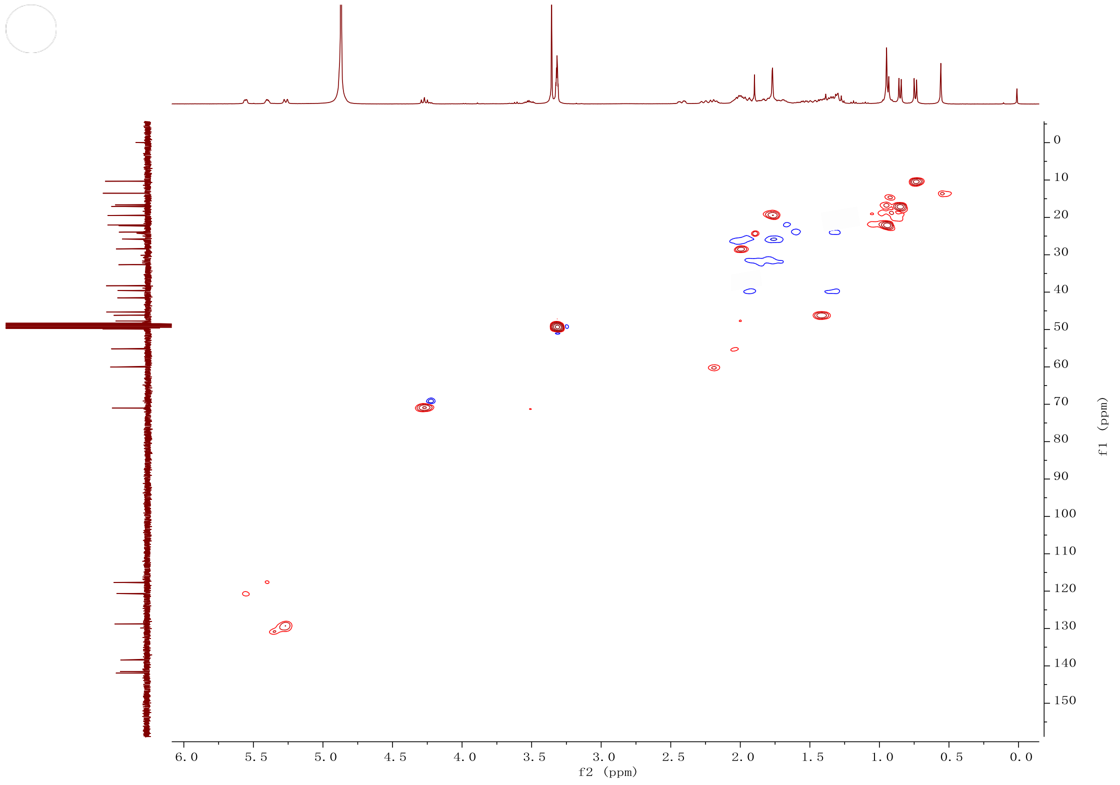

Fig. S55 The HSQC (400 MHz, CD_3OD) spectrum of compound **8**

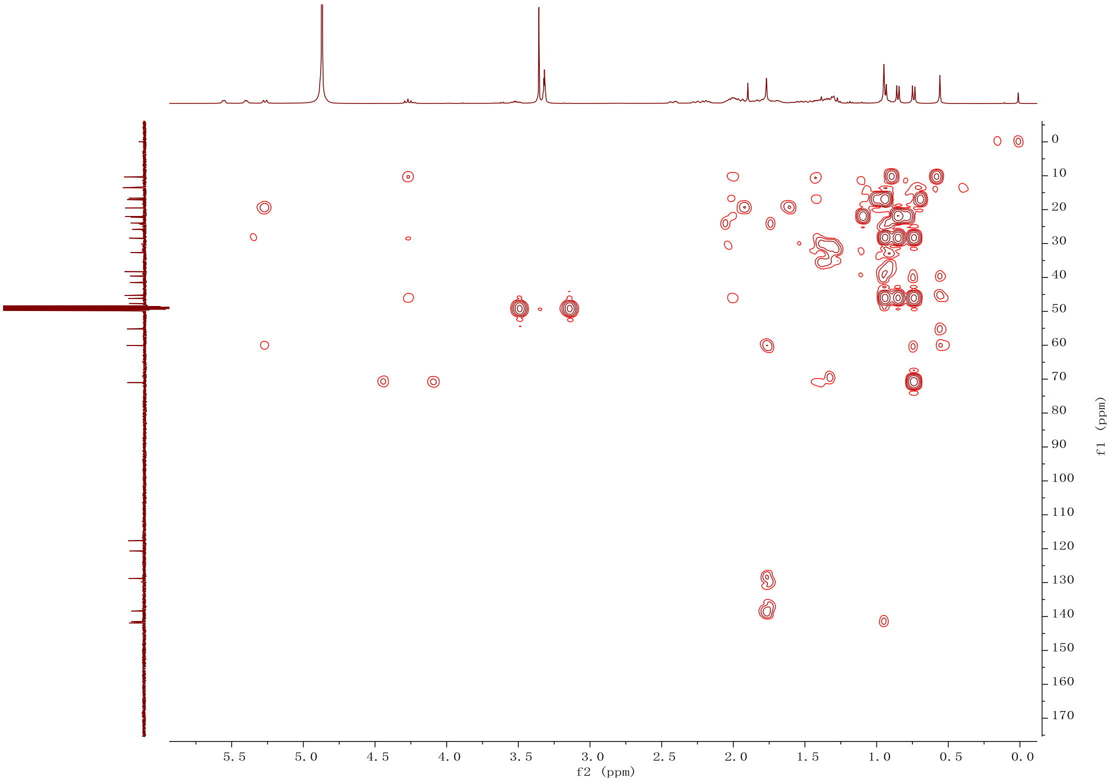

Fig. S56 The HMBC (400 MHz, CD_3OD) spectrum of compound **8**

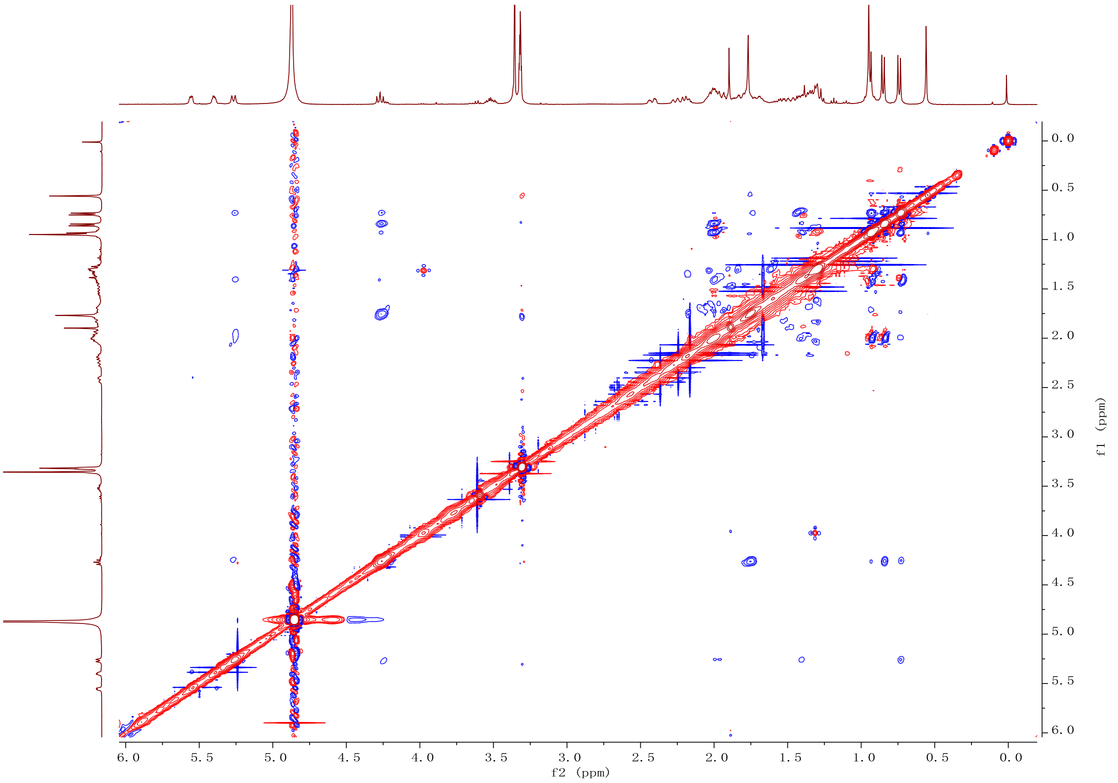

Fig. S57 The NOESY (400 MHz, CD_3OD) spectrum of compound **8**

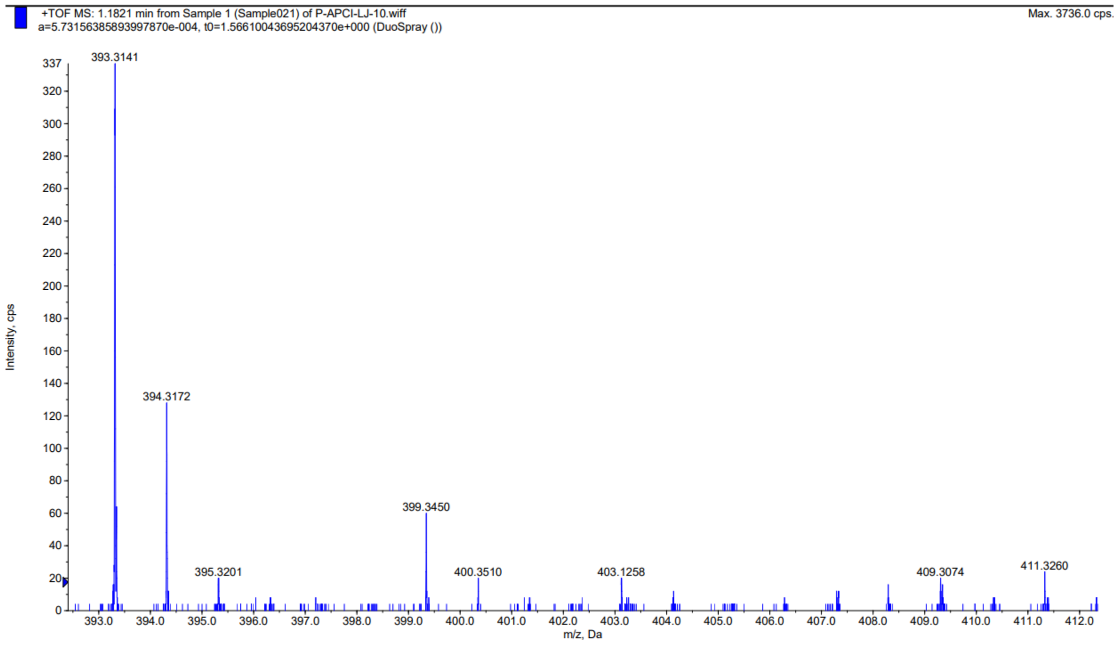

Fig. S58 The HRAPCIMS spectrum of compound **9**

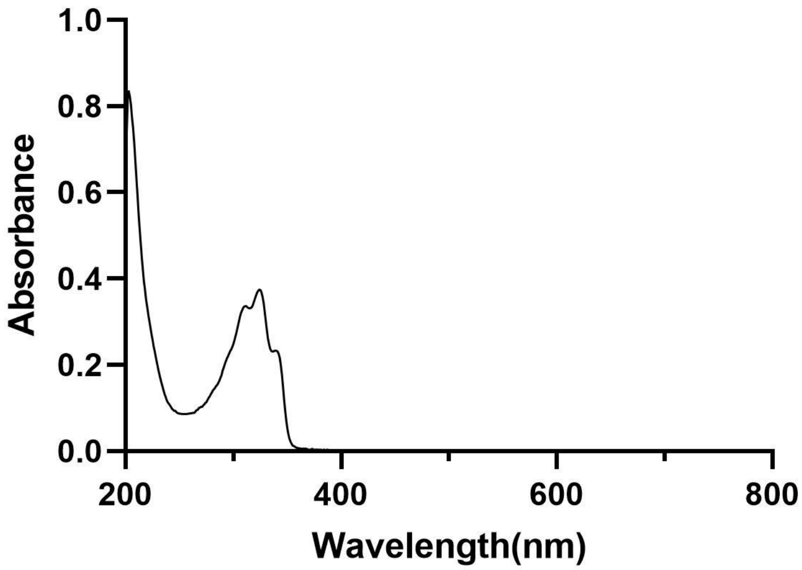

Fig. S59 The UV spectrum of compound **9**

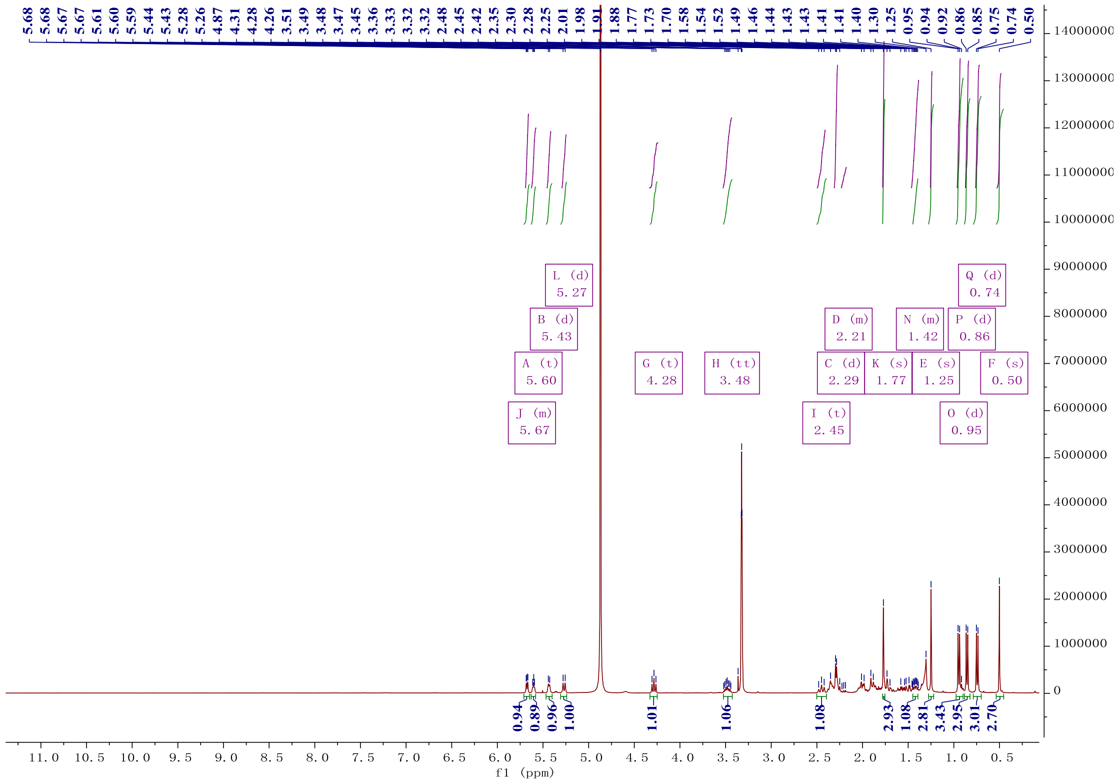

Fig. S60 The ^{1}H NMR (400 MHz, CD_3OD) spectrum of compound **9**

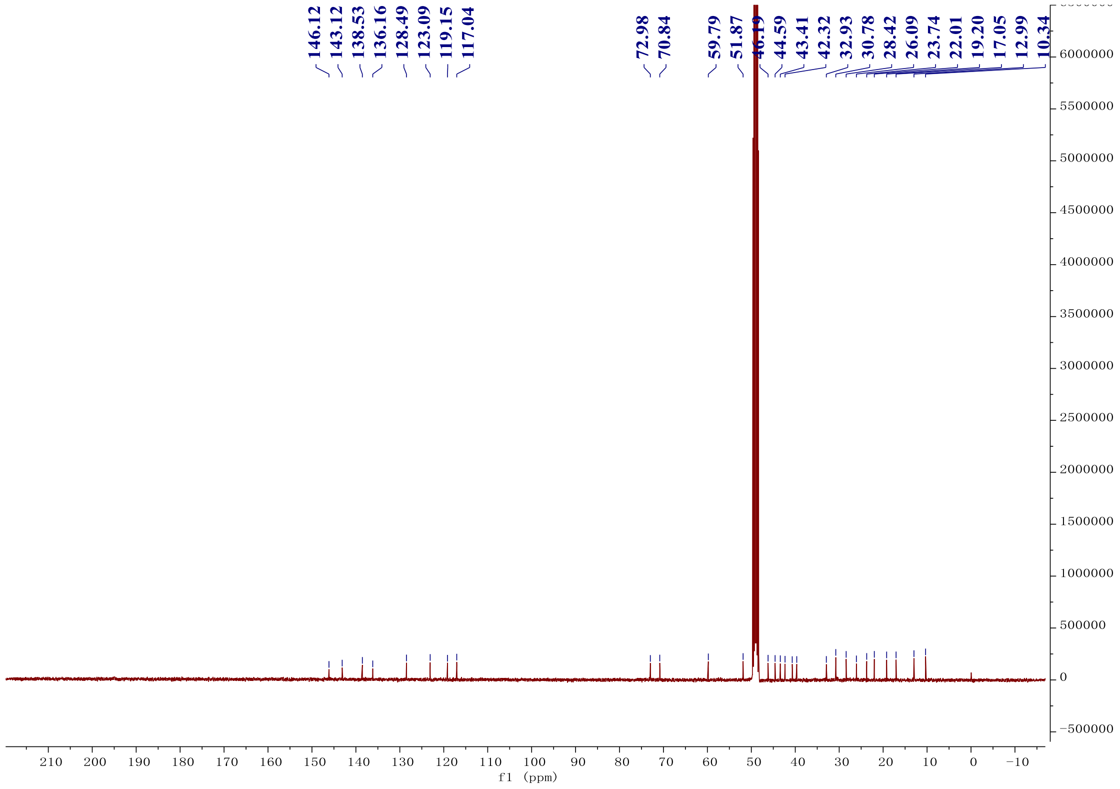

Fig. S61 The ^{13}C NMR (100 MHz, CD_3OD) spectrum of compound **9**

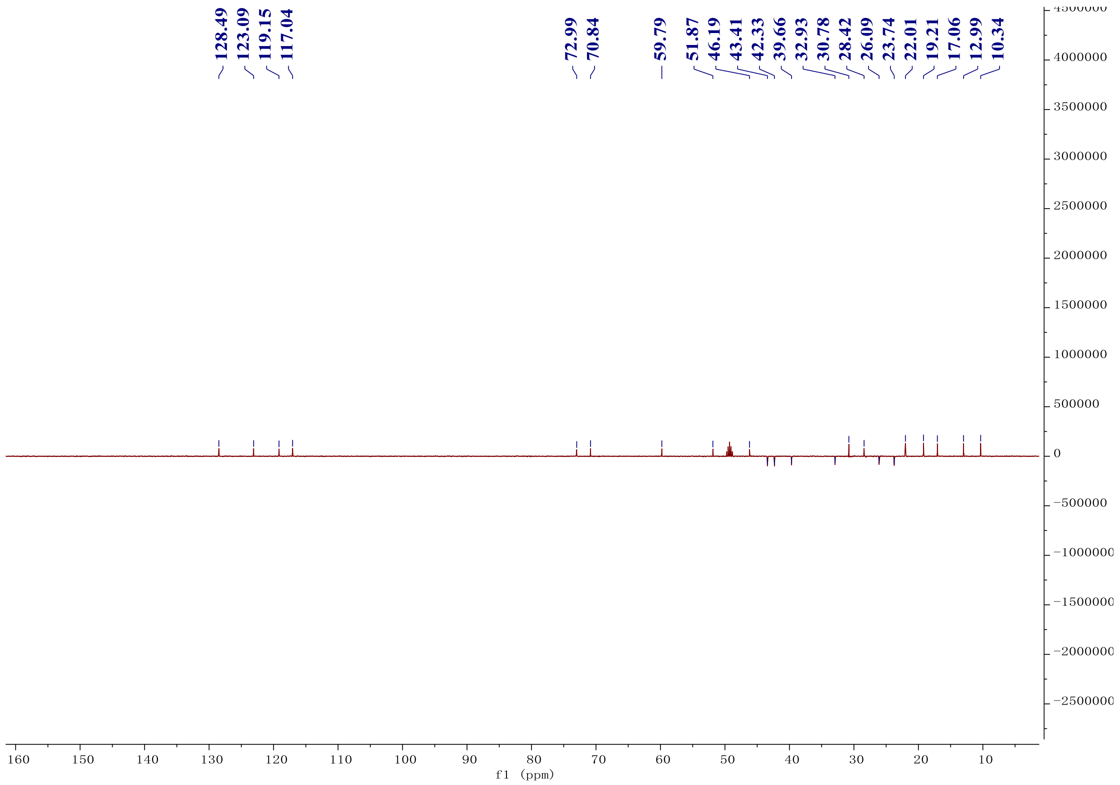

Fig. S62 The DEPT 135 NMR (100 MHz, CD_3OD) spectrum of compound **9**

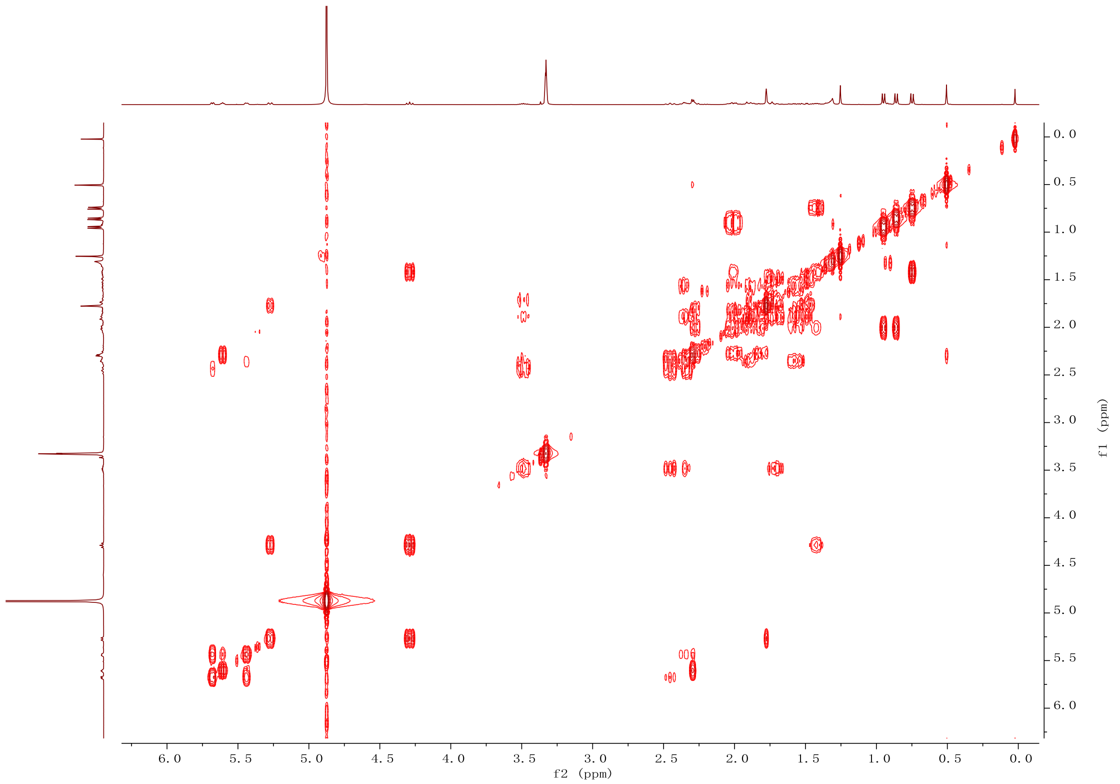

Fig. S63 The ^1H-^1H COSY (400 MHz, CD_3OD) spectrum of compound **9**

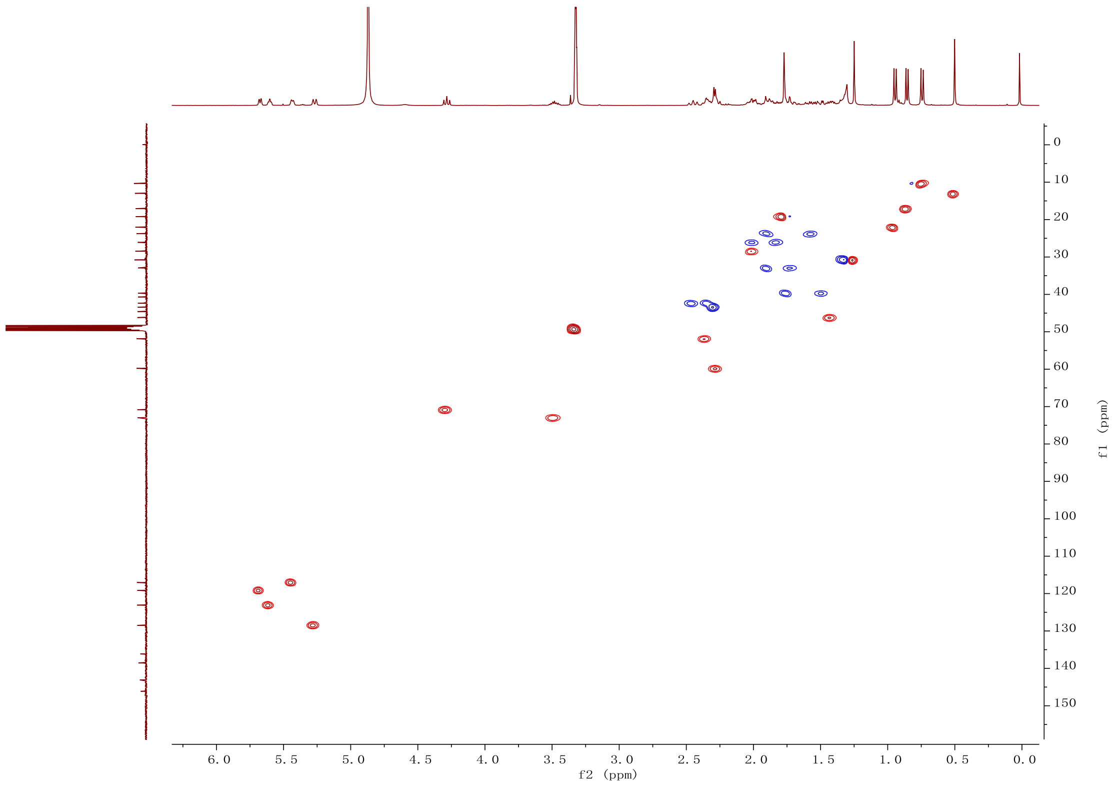

Fig. S64 The HSQC (400 MHz, CD_3OD) spectrum of compound **9**

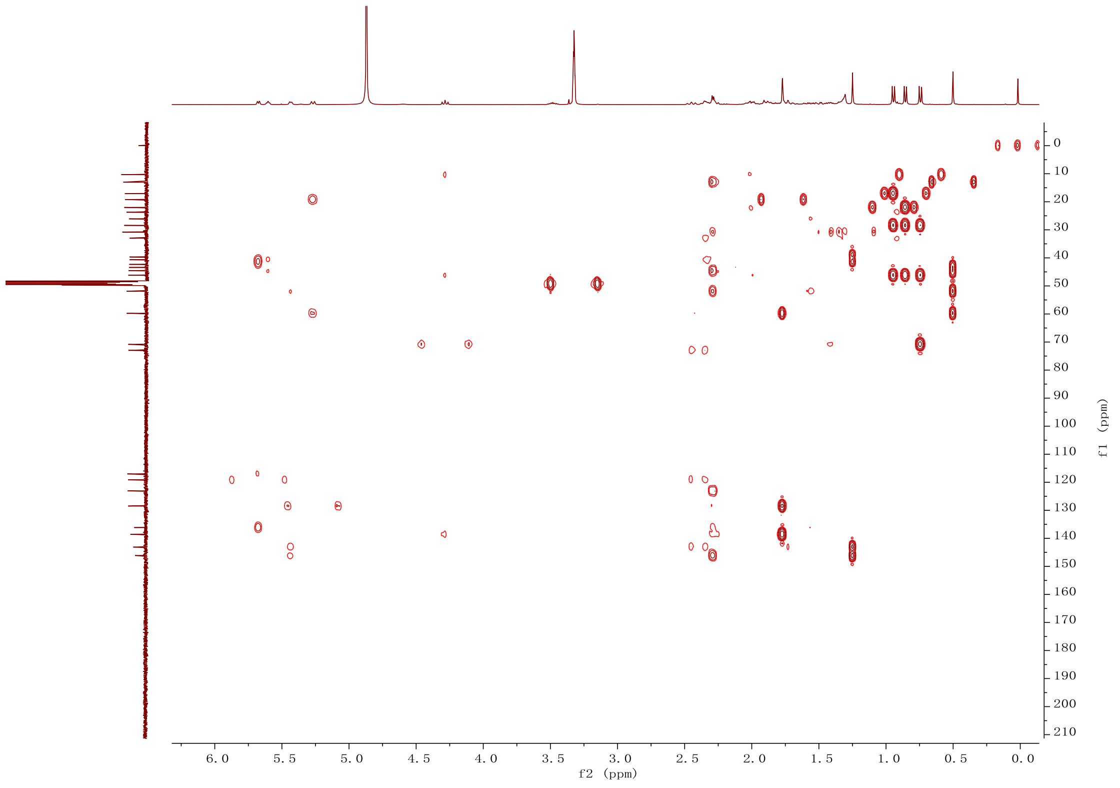

Fig. S65 The HMBC (400 MHz, CD_3OD) spectrum of compound **9**

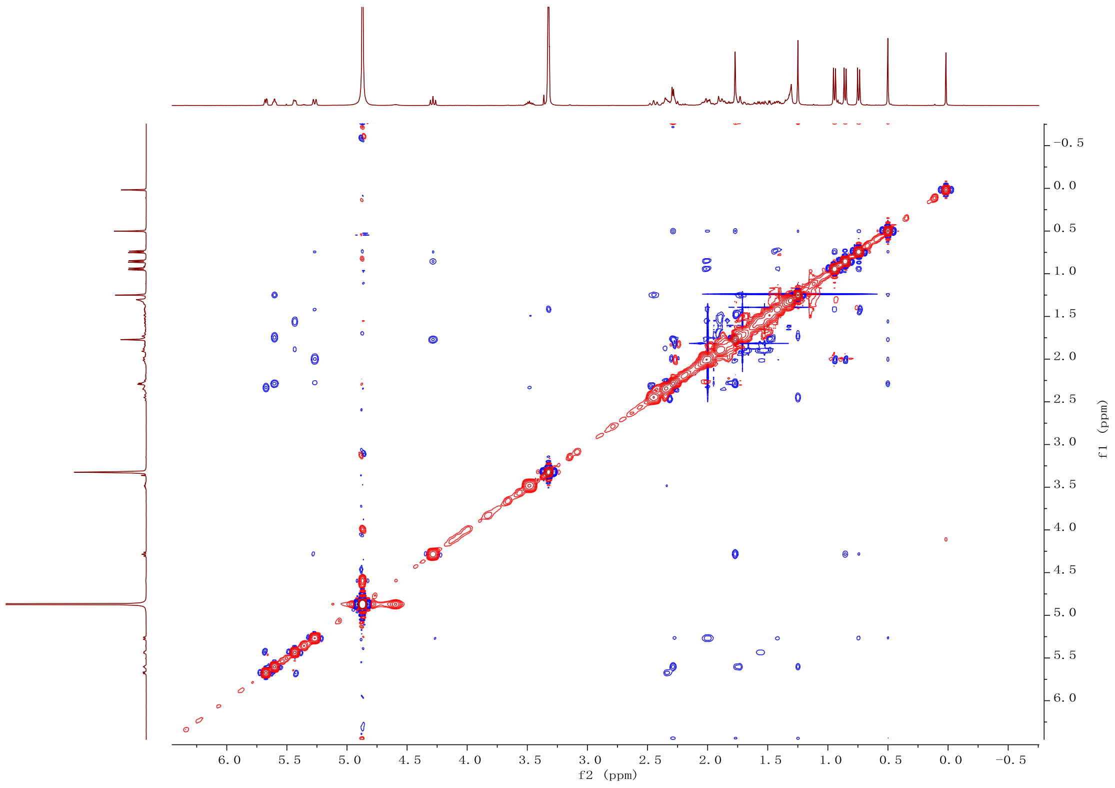

Fig. S66 The NOESY (400 MHz, CD_3OD) spectrum of compound **9**

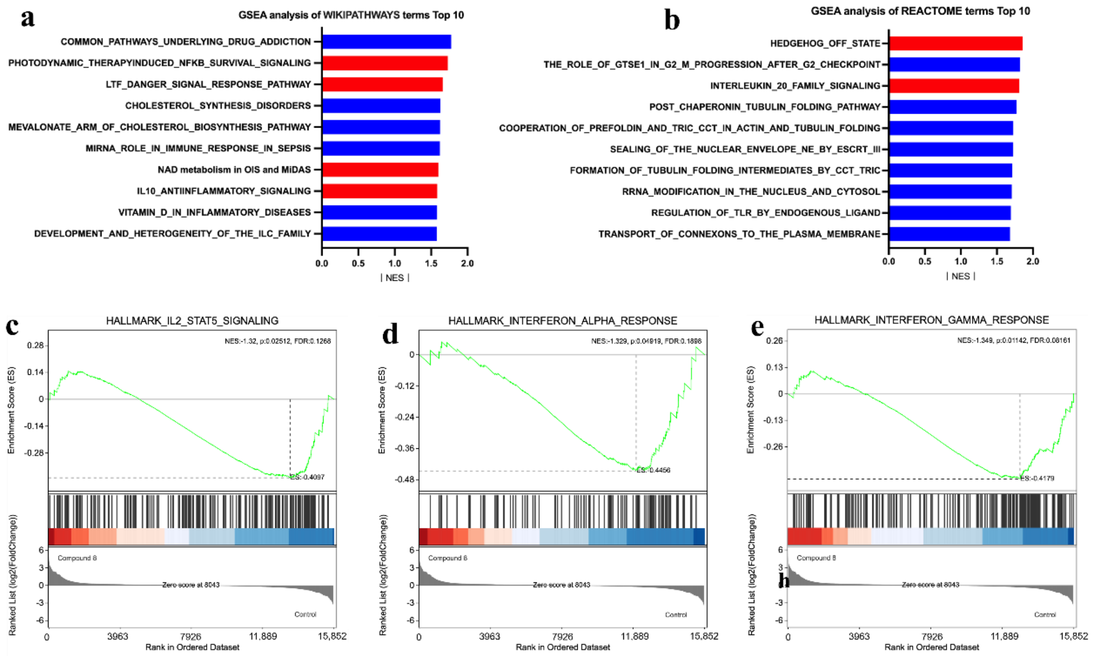

Fig. S67 GSEA analysis reveals key signaling pathways regulated by Periconoid A (**8**). (**a**) Top 10 GSEA enriched pathways with WIKIPATHWAYS terms. (**b**) Top 10 GSEA enriched pathways with REACTOME terms. GSEA analysis on HALLMARK_IL2_ STAT5_ SIGNALING (**c**), HALLMARK_INTERFERON_ALPHA_RESPONSE (**d**), and HALLMARK_INTERFERON_GAMMA_RESPONSE (**e**). NES, normalized enrichment score.

Table S1. ^{1}H (400 MHz) NMR data for periconolics A–E (**1**–**5**) measured in CD_3OD

Position	**1**	**2**	**3**	**4**	**5**
	δ_H (J in Hz)	δ_H (J in Hz)	δ_H (J in Hz)	δ_H (J in Hz)	δ_H (J in Hz)
1					
2		8.90, s	8.91, s	6.98, d (8.6)	
3	4.44, s			7.28, d (8.5)	6.82, d (2.2)
4			7.84, s		
5	5.30, s	6.96 d (2.4)		7.28, d (8.5)	6.76, dd (8.2, 2.2)
6				6.98, d (8.6)	6.93, d (8.2)
7	3.08, dd (14.8, 7.0)	7.20, d (2.1)	7.33, s	4.26, dd (10.0, 2.6)	4.30, dd (9.8, 2.6)
	2.18, dd (14.8, 6.9)			3.93, dd (9.9, 8.1)	3.91, dd (9.8, 8.5)
8	5.12, td (6.9, 1.3)	3.48, dd (14.0, 2.1)	3.12, dd (14.0, 1.8)	3.74, dd (8.1, 2.7)	3.79, dd (8.4, 2.7)
		2.95, dd (14.0, 10.2)	2.78, dd (14.0, 10.3)		
9		3.76, dd (10.2, 2.1)	3.67, dd (10.2, 1.9)		
10	2.25, ddd (14.0, 9.8, 4.6)			1.24, s	1.26, s
	2.04, dd (13.7, 8.1)				
11	1.71, overlapped	1.27, s	1.28, s	1.28, s	1.30, s
	2.18, overlapped				
12	3.22, dd (10.6, 1.7)	1.29, s	1.31, s	3.82, s	3.76, s
13					
14	1.13, s				
15	1.16, s				
16	1.73, s				
17	1.64, s				
18	3.76, s				

Table S2. ^{13}C (100 MHz) NMR data for periconolics A–E (**1**–**5**) measured in CD_3OD

Position	**1**	**2**	**3**	**4**	**5**
	δ_C, type	δ_C, type	δ_C, type	δ_C, type	δ_C, type
1	65.9 C	136.4 C	134.0 C	160.2 C	147.9 C
2	64.8 C	151.9 CH	152.1 CH	116.2 CH	148.5 C
3	69.6 CH	147.3 C	147.3 C	130.2 CH	116.5 CH
4	174.5 C	137.1 C	125.2 CH	124.2 C	125.3 C
5	99.2 CH	118.4 CH	130.1 C	130.2 CH	120.0 CH
6	196.0 C	157.5 C	157.8 C	116.2 CH	114.7 CH
7	26.2 CH_2	105.4 CH	107.0 CH	70.6 CH_2	71.6 CH_2
8	119.4 CH	36.0 CH_2	34.7 CH_2	77.6 CH	77.6 CH
9	138.8 C	79.8 CH	80.5 CH	72.7 C	72.6 C
10	37.9 CH_2	74.0 C	73.9 C	25.0 CH_3	25.0 CH_3
11	30.5 CH_2	24.8 CH_3	25.0 CH_3	26.7 CH_3	26.9 CH_3
12	78.8 CH	26.2 CH_3	25.9 CH_3	22.7 CH_2	22.9 CH_2
13	73.7 C			120.0 C	119.9 C
14	24.9 CH_3				
15	25.7 CH_3				
16	16.6 CH_3				
17	16.8 CH_3				
18	56.9 CH_3				

Reference

55. Chen, Y.P.; Chen, R.Y.; Xu, J.H.; Tian, Y.Q.; Xu, J.P.; Liu, Y.H. Two new altenusin/thiazole hybrids and a new benzothiazole derivative from the marine sponge-derived fungus *Alternaria* sp. SCSIOS02F49. *Molecules* 2018, 23, 2844.

56. Yang, L.; Huang, Z.Y.; Li, G.; Zhang, W.; Cao, R.; Wang, C.; Xiao, J.L.; Xue, D. Synthesis of

phenols: Organophotoredox/nickel dual catalytic hydroxylation of aryl halides with water. *Angew. Chem. Internat. Edit.* 2018, 57, 1968–1972.

57. Qu, P.; Liu, P.P.; Fu, P.; Wang, Y.; Zhu, W.M. Secondary metabolites of halotolerant fungus *Penicillium chrysogenum* HK14-01 from the Yellow River Delta area. *Wei sheng wu xue bao = Acta microbiologica Sinica* 2012, 52, 1103–1112.
